# Biomarkers for myalgic encephalomyelitis/chronic fatigue syndrome (ME/CFS): a systematic review

**DOI:** 10.1186/s12916-023-02893-9

**Published:** 2023-05-24

**Authors:** Rebekah Maksoud, Chandi Magawa, Natalie Eaton-Fitch, Kiran Thapaliya, Sonya Marshall-Gradisnik

**Affiliations:** 1 National Centre for Neuroimmunology and Emerging Diseases (NCNED), Menzies Health Institute Queensland, Griffith University, Gold Coast, Australia; 2 Consortium Health International for Myalgic Encephalomyelitis, Griffith University, Gold Coast, Australia; 3 School of Pharmacy and Medical Science, Griffith University, Gold Coast, Australia

**Keywords:** Myalgic encephalomyelitis, Chronic fatigue syndrome, Biomarker, Diagnostic test

## Abstract

**Background:**

Myalgic encephalomyelitis/chronic fatigue syndrome (ME/CFS) is a multifaceted condition that affects most body systems. There is currently no known diagnostic biomarker; instead, diagnosis is dependent on application of symptom-based case criteria following exclusion of any other potential medical conditions. While there are some studies that report potential biomarkers for ME/CFS, their efficacy has not been validated. The aim of this systematic review is to collate and appraise literature pertaining to a potential biomarker(s) which may effectively differentiate ME/CFS patients from healthy controls.

**Methods:**

This systematic review was conducted according to the Preferred Reporting Items for Systematic Reviews and Meta-Analyses and Cochrane review guidelines. PubMed, Embase and Scopus were systematically searched for articles containing “biomarker” and “ME/CFS” keywords in the abstract or title and if they included the following criteria: (1) were observational studies published between December 1994 and April 2022; (2) involved adult human participants; (3) full text is available in English (4) original research; (5) diagnosis of ME/CFS patients made according to the Fukuda criteria (1994), Canadian Consensus Criteria (2003), International Consensus Criteria (2011) or Institute of Medicine Criteria (2015); (6) study investigated potential biomarkers of ME/CFS compared to healthy controls. Quality and Bias were assessed using the Joanna Briggs Institute Critical Appraisal Checklist for Case Control Studies.

**Results:**

A total of 101 publications were included in this systematic review. Potential biomarkers ranged from genetic/epigenetic (19.8%), immunological (29.7%), metabolomics/mitochondrial/microbiome (14.85%), endovascular/circulatory (17.82%), neurological (7.92%), ion channel (8.91%) and physical dysfunction biomarkers (8.91%). Most of the potential biomarkers reported were blood-based (79.2%). Use of lymphocytes as a model to investigate ME/CFS pathology was prominent among immune-based biomarkers. Most biomarkers had secondary (43.56%) or tertiary (54.47%) selectivity, which is the ability for the biomarker to identify a disease-causing agent, and a moderate (59.40%) to complex (39.60%) ease-of-detection, including the requirement of specialised equipment.

**Conclusions:**

All potential ME/CFS biomarkers differed in efficiency, quality, and translatability as a diagnostic marker. Reproducibility of findings between the included publications were limited, however, several studies validated the involvement of immune dysfunction in the pathology of ME/CFS and the use of lymphocytes as a model to investigate the pathomechanism of illness. The heterogeneity shown across many of the included studies highlights the need for multidisciplinary research and uniform protocols in ME/CFS biomarker research.

**Supplementary Information:**

The online version contains supplementary material available at 10.1186/s12916-023-02893-9.

## Background

Myalgic encephalomyelitis/chronic fatigue syndrome (ME/CFS) is a debilitating condition that may affect between 17 and 24 million people worldwide [1]. The clinical presentation of this illness is complex. Patients experience a heterogeneous array of symptoms that fluctuate over time and affect multiple body systems including, but not limited to, immunological, neurological, endocrinological, and cardiovascular manifestations [2]. Severity of symptoms range between patients from mild to severe where some patients are wheelchair or bedbound [2].

There is currently no definitive diagnostic test; however, there is a significant body of biological evidence for this condition. Currently, ME/CFS is diagnosed using self-report case criteria following exclusion of any other potential medical explanation [3]. A systematic review by Brurberg et al. identified 20 different definitions used for ME/CFS [3]. The most frequent definitions commonly used in research and clinical practice include the following: the Fukuda criteria (FC), the Canadian Consensus Criteria (CCC), and the International Consensus Criteria (ICC) [2, 4, 5]. The Institute of Medicine (IOMC) also released a definition of ME/CFS in 2015 that is sometimes used in research [6]. One of the major differentiating features between the later definitions is the cardinal symptom “post-exertional neuroimmune exhaustion” (PENE). PENE occurs upon exertion that results in severe neuroimmune responses [2, 5]. Lack of definitive biomarkers complicates diagnosis and management of ME/CFS resulting in a significant number of people that remain undiagnosed. Furthermore, the reliance on exclusion of other causes of symptoms also results in delays in diagnosis as well as increased costs due to resource consumption [7].

A biomarker is defined as biological entity that can effectively and objectively differentiate one condition from healthy controls (HC) or from another condition [8]. A biomarker can range from, radiological, physical- or laboratory-based [9]. An optimal biomarker should have a clinical endpoint that is measurable over a short period of time with minimal variability and have a sufficiently higher signal-to-noise ratio [9]. Byrnes and Weigl described key features for optimal selection of biomarkers including trade-offs between complexity of detection, sensitivity/specificity and selectivity its ability to identify disease-causing agents [10].

Despite no definitive consensus on a molecular biomarker multiple research studies have investigated the prognostic potential of some markers for ME/CFS. This systematic review aims to collate evidence on suggested biomarkers for ME/CFS, critically examine their methodology, and evaluate their efficacy as well as their  suitability as a biomarker for ME/CFS.

## Methods

### Literature search

This systematic review was conducted according to the Preferred Reporting Items for Systematic Reviews and Meta-analyses (PRISMA) and Cochrane review guidelines [11, 12]. Prior to commencement, our protocol was compared with published listings on the PROSPERO (National Institute for Health Research) for duplication and prospectively registered on the database (ID: CRD42022293059). The following databases PubMed, Embase and Scopus were systematically searched for literature with biomarker key words in conjunction with fatigue syndrome, chronic (a full comprehensive search code can be found in Additional File 1). The Boolean operator “AND” was used to combine the terms. [abstract/title] filters were applied to confine search to key terms existing only in the abstract and title. The search was expanded to include all Medical Subject Headings (MeSH) terms. Reference list checking and a citation search was conducted but no additional publications were retrieved. All included articles underwent peer review. Unpublished literature or pre-print databases such as medRxiv or BioRxiv were not searched. The literature search was conducted on 30th May 2022.

### Inclusion criteria

Studies were selected if they complied with the following inclusion criteria: (1) observational study published after 1994; (2) study involved human participants aged 18 years or older; (3) full text available in English; (4) original research; (5) diagnosis of ME/CFS patients were made according to the FC (1994), CCC (2003), ICC (2011), or IOMC (2015); (6) study investigated potential biomarker/s of ME/CFS compared to HC.

### Exclusion criteria

Studies were excluded if they met any of the following exclusion criteria: (1) was an interventional study; (2) written prior to the establishment of the  FC in December 15th 1994; (3) not available as full text or written in English; (4) was not an original study type including reviews, duplicate studies, or case reports; (5) use of other diagnostic criteria instead of FC, CCC, ICC, IOMC; (6) studies that are not within the scope of this review or did not compare to HC.

### Selection of studies

Duplicates were removed using Endnote version 20 (Endnote, Clarivate™, Philadelphia, USA) through the automated duplicate removal tool. The references were scanned for any remaining duplicates which were manually removed. The reference management tool Rayyan was used to sort and store the remaining publications [13]. Abstracts were first screened for suitability, followed by a full-text screening. These processes were conducted independently by RM and CM and any discrepancies between the two authors were compared and resolved through discussion. The final articles that were included were reviewed and validated by all listed authors.

### Data extraction

Following the selection of papers, relevant data were manually extracted including (1) study type; (2) criteria; (3) sample size (4) age; (5) sex; (6) body mass index (BMI); (7) illness duration; (8) biomarker; (9) classification (10) selectivity; (11) ease of detection; and (12) receiver operator characteristic (ROC) area under the curve (AUC)/sensitivity/specificity/accuracy; (13) findings. Studies were sorted according to type of biomarker from the following: genetic, immunological, metabolomics/mitochondrial/microbiome, endovascular/circulatory, neurological, ion channel and physical biomarkers. Due to lack of homogeneity between studies, a meta-analysis could not be conducted. Selectivity and ease of detection were included based on Byrnes and Weigl’s analytical biomarker for diagnostic application framework [10]. Selectivity refers to the ability of the biomarker to identify a disease-causing agent ranging from primary (direct) to tertiary (indirect) and ease of detection refers to the complexity of biomarker identification ranging from easy to complex.

### Quality analysis

All publications were assessed for quality and bias using the Joanna Briggs Institute Critical Appraisal Checklist for Case Control Studies (JBI CACCCS). JBI CACCCS was selected for quality analysis as the checklist is validated and internationally recognised [14]. This checklist has also been updated to reflect advancements in bias risk assessment and adheres with requirements for the current PRISMA guidelines [14]. Quality analysis was conducted independently by two authors (RM and CM). Each checklist item assesses the following: (1) group matching; (2) source population; (3) criteria; (4) method of exposure; (5) assessment of exposure; (6) identification of confounding variables; (7) management of confounding variables; (8) measurement of outcomes; (9) exposure period selection; (10) statistical analysis. Items 4, 5, and 9 were excluded in selected papers as they did not have an exposure. JBI CACCCS is a qualitative checklist, and there is no incorporation of a scoring system; hence, no studies were omitted based on quality [14].

## Results

**Fig. 1:**
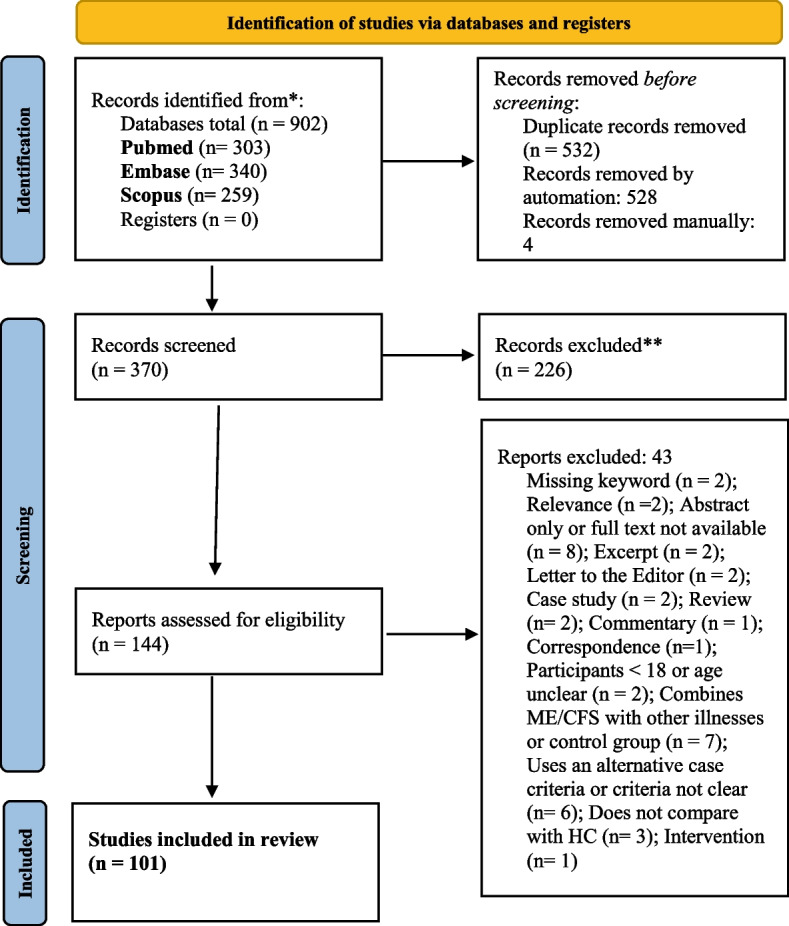
PRISMA flow diagram of systematic search for biomarker systematic review

PubMed (303), Embase (340) and Scopus (259) retrieved a total of 902 articles. Following removal of duplicates and application of inclusion and exclusion criteria, the total number of articles were refined to 101. The systematic process as conducted by PRISMA guidelines is outlined in Fig. 1.

### Overview of articles

The study and participant characteristics as well as findings of each article is summarised in Additional File 2. In total, 98 out of 101 included publications were observational case–control studies [15–112].Two of these studies also included non-ME/CFS affected blood relatives in addition to HC [102, 109]. One publication was an observational twin study [113], and the remaining two publications were prospective longitudinal linked panel studies [114, 115].

### Participant and study characteristics

The average sample size of ME/CFS patients and HC included across all the studies was *n* = 43.83 and *n* = 38.34, respectively. The average age of ME/CFS patients was 45.40 ± 8.76 years old and the average age of HC was 43.43 ± 9.15. Females were more prominent whereby on average 76% of ME/CFS patients and 72% of HC were female. Mean illness duration for ME/CFS patients ranged between 4.5 and 22 years with the average duration being 10.30 years.

In total, 56 of the studies recruited patients that met the FC [15, 19, 21, 22, 24, 28, 31, 35–42, 44, 45, 49, 50, 52, 53, 59, 61–69, 71, 78, 79, 83, 84, 86–89, 92–95, 98, 99, 101, 102, 105, 108, 111–115]. Fourteen studies recruited patients that met the CCC [17, 23, 32, 46, 47, 57, 58, 73, 82, 91, 100, 103, 104, 107]. One study recruited patients diagnosed with the ICC [25], and another study recruited patients diagnosed with IOMC [34]. A combination of FC and CCC was used to diagnose patients in 19 studies [16, 18, 26, 27, 30, 48, 51, 54, 56, 60, 70, 72, 74–77, 85, 90, 97], a combination of FC and ICC was used to select patients in three studies [55, 106, 109], and a combination of CCC and ICC was used to select patients in one study [29]. A combination of the FC and IOMC was used in one study [41]. Kitami et al. recruited patients that met either the FC, ICC or IOMC [55]. The remaining studies recruited a combination of patients diagnosed with the FC, CCC and ICC definitions [80, 81].

In total, 76 of the biomarkers were blood-based (75.25%). Most of the biomarkers had either secondary (43.56%) or tertiary (53.47%) selectivity [15–47, 49–90, 92–96, 98–115] and had moderate (59.40%) to complex (39.60%) ease-of-detection including the involvement of specialised equipment [15–37, 39–115]. AUC, sensitivity, specificity, and accuracy were reported in only some of the studies (*n* = 35, 34.65%). Due to the limited number of studies that included these values, an average was not calculated.

The studies have been grouped according to type of biomarker in Additional File 2 including genetic (Table S1), immunological (Table S2), metabolomics/mitochondrial/microbiome (Table S3), endovascular/circulatory (Table S4), neurological (Table S5), ion channel (Table S6) and physical biomarkers (Table S7).

### Literature reporting on genetic/epigenetic biomarkers

There were 20 studies that discussed genetic/epigenetic biomarkers in ME/CFS patients compared with HC (additional File 2, table S1) [16, 18, 20, 21, 25, 27, 43, 52, 59, 60, 67, 68, 71, 82, 87, 88, 92, 103, 108, 111]. Two of the included publications investigated deoxyribonucleic acid (DNA) changes through single-nucleotide polymorphisms (SNPs) [67, 68]. Eleven SNPs in natural killer (NK) cells from ME/CFS patients were identified by Marshall-Gradisnik et al., and SNPs in five of these SNPs were associated with transient receptor potential melastatin 3 (TRPM3). There were 14 SNPs that were associated with nicotinic and muscarinic genes. From these SNPs, 16 genotypes were identified [67]. In ME/CFS B cells, 78 SNPs were found where 35 of these changes occurred in muscarinic acetylcholine receptor M3 (mAChM3) [68]. Other SNPs that were identified included nicotinic acetylcholine receptor delta, nicotinic acetylcholine receptor alpha 9, TRPV2, TRPM3, TRPM4, mAChRM3 and cholinergic receptor muscarinic 5 [68]. 

Six studies reported altered miRNA in ME/CFS patients compared with HC [18, 20–22, 82, 87]. Three studies investigated levels of miRNA-21 (miR-21) [16, 18, 21]. The levels of miR-21 were variable across the studies [16, 18, 21]. Blauensteiner et al. reported higher levels of miR-21, miR‑34a, miR‑92a, miR‑126 and miR‑200c in plasma of ME/CFS patients compared with HC [18]. Brenu et al. reported a significantly lowermiR-21 in CD8^+^ T cells and NK cells in ME/CFS patients compared with HC [21]. In extracellular vesicles (EV), differences in miR-21 between ME/CFS and HC did not reach significance [16]. Almenar-Pérez et al. found that there were 17 miRNAs that were differentially expressed in peripheral blood mononuclear cells (PBMC) [16].

Brenu et al. reported that hsa-miR-127-3p, hsa-miR-142-5p, and hsa-miR-143-3p were significantly upregulated in ME/CFS patients using a combination of high- throughput sequencing and real-time quantitative polymerase chain reaction analysis [20]. Nepotchatykh et al. assessed circulating miRNA signatures following induction of post-exertional malaise (PEM) [82]. This study comprised of a discovery phase to identify potential differentially expressed miRNA [82]. Seventeen miRNAs were identified and following individual detection of miRNA, machine learning methods verified 11 of these miRNA as significant [82]. At baseline, hsa-miR-28-5p, hsa-miR-127-3p, hsa-miR-140-5p, hsa-miR-374b-5p, hsa-miR4433a-5p, and hsa-miR-6819-3p were significantly higher in ME/CFS patients compared with HC. Expression levels of hsa-miR-150-5p, hsa-miR-486-5p, and hsa-miR-3620-3p were significantly elevated following PEM induced after 90 min of stimulation [82]. Petty et al. identified 34 significantly upregulated miRNA markers in ME/CFS patients compared with HC. The most profound differences occurred in NK cells [87].

Eight studies investigated changes in mRNA. All studies described significantly differentially expressed mRNA [25, 43, 59, 60, 71, 88, 92, 111]. One study detected up to 366 differentially expressed genes in ME/CFS patients compared with HC [43]. Chacko et al. reported that there were 92 differentially expressed protein kinase genes: 37 genes that were significantly upregulated and 55 genes that were significantly downregulated in severe ME/CFS patients compared to HC [25]. White et al. found that mRNA in P2X4, TRPV1 (vanilloid), CD14 and adrenergic receptors was elevated in ME/CFS patients compared with HC post-exercise [111]. ME/CFS patients with comorbid fibromyalgia also showed higher levels of mRNA in acid-sensing ion channel 3 (ASIC3) and P2X5. Elevated mRNA in P2X4, P2X5 and ASIC3 was also described by Light et al. in ME/CFS patients following exercise [59]. This increase was transient and occurred between 0.5 and 48 h post-exercise. In the same study, alpha-2A, beta-1, beta-2, catechol-O-methyltransferase, interleukin (IL)-10 and toll-like receptor 4 were also higher in ME/CFS patients [60]. An increase in mRNA in most sensory and adrenergic receptors post-exercise for 48 h was described in 71% of patients with ME/CFS in a later study by Light et al. [59]. The increase in mRNA post-exercise was associated with pain and fatigue. In the remaining 29% of ME/CFS patients, adrenergic α-2A mRNA was lower compared with HC and this was more common in patients with orthostatic intolerance [59]. Iacob et al. reported that greater gene expression of purinergic and cellular modulators and nociception and stress mediators were positively associated with a diagnosis of ME/CFS [52].

Two studies investigated epigenetic modification in ME/CFS patients compared with HC [27, 71]. De Vega et al. reported 12,608 differentially methylated sites in ME/CFS patients compared with HC [27]. Metselaar et al. found that there were 48 CpGs that were predictive of ME/CFS [71]. Metselaar et al. utilised data from De Vega et al. in combination with other published datasets in their analysis [27, 71].

### Literature reporting on immunological biomarkers

Thirty studies investigated immune cell function changes in ME/CFS patients compared with HC (Additional File 2, Table S2) [22, 26, 33, 36, 37, 45, 46, 48–51, 54, 56, 61, 62, 64, 65, 72, 84, 91, 97, 99, 102, 104, 107, 109, 110, 114, 115]. Five of the studies reported reduced NKCC in ME/CFS patients compared with HC [37, 65, 67, 102, 114]. Other NK cell-specific features include lower expression levels of DPPIV/C26 in ME/CFS [37]. Brenu et al. described higher levels of the lytic protein perforin, but lower levels of Granzyme A and Granzyme K in NK cells of ME/CFS patients compared with HC [22]. One study, Cliff et al., reported no significant differences in NK cell numbers, subtype proportions and cell cytotoxicity in frozen biobanked samples [26]. Theorell et al. found that there were no significant changes in cytotoxic lymphocytes including NK cell and T cell population phenotype and function in ME/CFS compared with HC [107]. Brenu et al. assessed NKCC longitudinally over a 12-month period. Differences in NKCC were significantly different across the three timepoints, and NKCC was consistently lower in ME/CFS patients compared with HC across the different timepoints [114]. Other NK cell subsets such as NKCD69 and NKCD56 were reported to be higher in ME/CFS patients [91]. Hardcastle et al. found that there were significantly reduced CD56^dim^CD16^−^ NK cell CD2+ and CD18^+^ CD2^+^. Severe ME/CFS patients had significantly increased CD18^+^ CD11c^−^ in the CD56^dim^CD16^−^ NK cell phenotype and significantly reduced NKp46 in CD56^bright^CD16^dim^NK cells [50]. Hanevik et al. reported that post-giardiasis ME/CFS patients had significantly lower NK cell levels compared to HC [49]. The complement pathway post-exercise was investigated in two studies [84, 99]. Sorensen et al. found that complement split product C4a was significantly higher in ME/CFS patients compared with HC six hours post-exercise [99]. Conversely, Nijs et al. reported no difference in complement split product levels as well as elastase activity or IL-1β [84].

Higher non-classical monocytes were found in ME/CFS patients compared with HC [109]. There was no significant difference, however, between patients and non-affected family members [109]. Sung et al. also examined differences between ME/CFS patients, related family members and HC [102]. Both ME/CFS patients and related family members showed lower antibody-dependent cell-mediated cytotoxicity (ADCC) compared with HC [102]. Autoantibodies were measured in four studies [48, 62, 104, 110]. Vernon et al. reported no significant autoantibodies across the whole ME/CFS group compared with HC [110]. Szklarski et al. found that there was an association with lower levels of sCD26 and increased levels of autoantibodies against adrenergic receptors (AR) and mAChR3 [104]. Maes et al. identified that plasma peroxide and oxidised low-density lipoprotein antibody concentrations were significantly higher in ME/CFS patients compared to HC [62]. There was low association with these antibodies and the fibromyalgia and chronic fatigue syndrome rating scale [62]. In a study conducted by Halpin et al., ME/CFS patients had higher levels of anti-EBV-dUTPase antibodies and anti-human dUTPase antibodies compared to HC [48]. ME/CFS patients with coexisting irritable bowel syndrome (IBS) showed an association with immunoglobulin lambda constant region 7 and ME/CFS patients without IBS showed association with immunoglobulin kappa variable region 3–11[72]. In ME/CFS with post-infectious onset, sCD26 levels were positively associated with activated T cells, liver enzymes, creatine kinase and lactate dehydrogenase. Rivas et al. reported significantly lower values of T regulatory cells in ME/CFS patients compared with HC [91]. Two hundred and fifty six peptide signatures were detected that could significantly differentiate between ME/CFS patients and HC based on AUC values [46]. Humoral immunity profiling identified 25 peptides that effectively differentiate between ME/CFS patients and HC [97]. An increase in activation of antigens on CD8^+^ T lymphocytes including CD3^+^, CD8^+^ , CD8^+ ^CD38^+^, and CD8^+^ HLA-DR + were described by Maes et al. [61]. Espinosa et al. found that there was a significant decrease in CD57 molecule expressed per T cell as well as percentage of cells expressing CD57 in ME/CFS patients compared to HC [33]. Hardcastle et al. reported that moderate ME/CFS patients had significantly increased CD8^+^CD45RA effector memory T cells, signalling lymphocytic activation molecule expression on NK cells, killer cell immunoglobulin-like receptor 2DL5A on CD4^+^ T cells, and BTLA4^+^ on CD4^+^ T central memory cells [50]. In a longitudinal study also conducted by Hardcastle et al., iNKT CD62L increased in expression over time in moderate ME/CFS patients. At six months, naïve CD8^+^ T cells, CD8^−^ CD4^−^ and CD56^−^CD16^−^ iNKT phenotypes, γδ2T cells and effector memory subsets were significantly increased in severe ME/CFS patients. Severe ME/CFS also had significantly reduced CD56^bright^CD16^dim^ NKG2D, CD56^dim^CD16^−^ KIR2DL2/DL3, CD94^−^CD11a−γδ1T cells, and CD62L^+ ^CD11a^−^ γδ1T cells at six months [115].

Nine studies investigated cytokines as a potential biomarker for ME/CFS [22, 28, 36, 45, 51, 53, 56, 64, 114]. The repertoire of cytokines being measured mostly varied between studies. The following cytokines were reported to be significantly higher in ME/CFS patients compared with HC: IL-1, TNF-a, IL-10, IL-13, IL-16, INF-g, and IL-17A [22, 53, 54, 56, 64]. In a study conducted by Fletcher et al., the following cytokines were elevated in ME/CFS patients compared to HC: LTα, IL-1α, IL-1β,IL-4, IL-5, IL-6, and IL-12 [36]. The following cytokines showed good biomarker potentials based on area under the curve: IL-5, LTα, IL-4, and IL-12 [36]. In contrast, Groven et al. reported that levels of IL-10, 1L-17, and TNF-α were significantly lower in ME/CFS patients [45]. Landi et al. reported significantly lower levels of IL-16 and IL-7 [56]. IL-17F and CXCL8 were found to be significantly lower in ME/CFS patients in a study conducted by Khaiboullina et al. [54]. Hornig et al. reported an increase in activation of pro- and anti-inflammatory cytokines in early stages of ME/CFS [51]. Higher levels of inflammatory cytokines were also reported by Domingo et al. A longitudinal study conducted by Brenu et al. found that there was significant variability of cytokine levels at different timepoints: baseline (T1), 6 months (T2) and 12 months (T3). After mitogenic stimulation, there were significant increases in IL-10, IFN-y and TNF-a at T1. IL-10, and IL-17A were significantly decreased at T2. IL-2 was increased at T3 in ME/CFS patients [114].

### Literature reporting on metabolomics/mitochondrial/microbiome biomarkers

Five studies reported on changes in metabolites in ME/CFS patients compared with HC (Additional File 2, Table S3) [17, 41, 42, 76, 96]. All of the studies investigated metabolites in blood [17, 41, 42, 76, 96]. There were no common significant metabolites between the studies. Armstrong et al. described lower glutamine levels in ME/CFS patients [17] and Germain et al. found high predictive value of pyroglutamine, a glutamine derivative [42]. Pathway analysis conducted by each of the studies suggested that disrupted metabolites may impact amino acid (mentioned in 4/5 studies) [17, 41, 42, 96] and/or energy metabolism pathways (mentioned in 3/5 studies) potentially involving the urea cycle [41, 42, 76]. ME/CFS patients have significantly lower levels of phosphatidylcholine, choline and carnitine compared to HC. ME/CFS patients with IBS had significantly higher levels of triglyceride and ceramide [76].

Changes in mitochondrial function and energy metabolism in ME/CFS patients compared to HC were investigated in four studies [28, 39, 73, 103]. Biological antioxidant potential (BAP) measurements are proportional to antioxidant capacity of a serum sample and oxidative activity is determined through measuring diacron reactive oxygen metabolites (d-ROMs) [39]. BAP, d-ROMs, and oxidative stress index were all significantly influenced by age. In the age-matched group, d-ROMs, and oxidative stress index were significantly higher in ME/CFS patients compared with HC. Further, workers who experience sub-acute fatigue had significantly higher values of d-ROMs, and OSI compared to HC and ME/CFS patients at rest [39]. Domingo et al. reported that ME/CFS patients had lower antioxidant capacity and higher lipoperoxide, fibroblast growth factor 21, and brain natriuretic peptide (NT-proBNP) [28]. Missailidis et al. found that differences in mitochondrial respiratory function in combination with target of rapamycin complex I activity and lymphocyte death rate can differentiate ME/CFS patients from HC with a sensitivity of 90% [73]. Sweetman et al. identified 60 differentially expressed proteins in ME/CFS patients that have important roles in mitochondrial and energy metabolism processes [103].

Four studies assessed differences in microbiome composition of ME/CFS patients and HC [55, 66, 77, 95]. Mandarano et al. found that there were no differences in eukaryotic diversity of gut microbiota in ME/CFS patients compared with HC [66]. In stool and plasma samples 72 h post-maximal exercise, there was an increase in relative abundance in six (66.7%) major bacterial phyla in ME/CFS compared with only two of the nine in HC [95]. Clearance of these bacteria in blood was also significantly slower in ME/CFS patients compared with HC [95]. Nagy-Szakal et al. examined faecal metagenomic profiles in ME/CFS patients with or without IBS compared with HC [77]. In general, ME/CFS patients had lower metabolic pathways associated with unsaturated fatty acid biosynthesis and increased atrazine degradation pathways independent of IBS comorbidity [77]. ME/CFS patients with IBS had significantly higher unclassified allistripes and less Faecalibacterium compared with HC. ME/CFS patients without IBS had greater unclassified *Bacteroides* and less *Bacteroides Vulgatus* [77].

### Literature reporting on endovascular/circulatory biomarkers

There were 13 studies that reported on endovascular/circulatory biomarkers (Additional file 2, Table S4) [16, 19, 24, 30, 34, 35, 44, 47, 57, 58, 63, 70, 74, 85, 94, 100, 101, 105]. Endothelial function was assessed in two studies [47, 100]. Flow-mediated dilation (FMD) and post-occlusive reactive hyperemia results suggest that endothelial function was much lower in ME/CFS patients compared with HC [100]. Five out of 14 post COVID-19 ME/CFS patients showed diminished reactive hyperaemia index compared with HC [47]. Haffke et al. also reported elevated endothelin-1 (ET-1) in ME/CFS patients and post COVID-19 condition patients as well as lower angiopoietin-2 in both groups compared with HC [47]. Sørland et al. reported no differences in levels of markers of endothelial function but the same metabolites as Haffke et al. were not investigated [47, 100].

Circulating protein-level differences in ME/CFS patients compared to HC were investigated in six studies [16, 44, 57, 58, 74, 105]. Serum activin B levels were measured in three studies [44, 57, 58]. One study reported no significant differences in activin B levels between ME/CFS patients and HC [44]. Two studies by Lidbury et al. found that serum activin B levels were significantly higher in ME/CFS patients compared with HC [57, 58]. Lidbury et al. also suggested that 24-h urinary creatinine clearance and serum urea were also significantly higher in ME/CFS patients [58]. Both studies were reporting on the same study cohort [57, 58]. Lower levels of serum creatine kinase in severe ME/CFS patients compared with HC and non-severe ME/CFS patients were described by Nacul et al. [74]. Significantly lower creatine kinase was also described by Almenar-Pérez et al. [16]. Thambirajah et al. found that basal HSP27 was significantly higher in ME/CFS patients compared to HC [105]. Levels of HSP27, HSP60 and HSP90 were also significantly decreased post-exercise in ME/CFS patients compared to HC [105].

Lipid-based products were investigated in six studies [16, 19, 24, 30, 34, 85]. Nkiliza et al. investigated sex-specific differences in plasma lipid profiles [85]. Male ME/CFS patients had significantly higher omega-6 linoleic acid-derived oxylipins compared with female ME/CFS patients [85]. Omega-6 linoleic acid-derived oxylipins were significantly higher compared to male HC. In females, phosphatidylinositol, saturated triglyceride levels and hexosylceramides were lower in ME/CFS patients compared with HC [85]. Circulating EV was investigated in four studies [16, 19, 24, 30]. Three studies found significantly higher circulating EV numbers in ME/CFS patients compared with HC [16, 24, 30]. Almenar-Pérez et al. and Castro Marrero et al. also reported EVs smaller in size [16, 24]. Additionally, Almenar-Pérez et al. found that zeta-potential was significantly different in ME/CFS patients compared to HC, where ME/CFS patients presented with more negative values regardless of whether the EV were isolated in the presence or absence of proteinase K [16]. Bonilla et al. found no association between severe ME/CFS and levels of EVs carrying the B cell marker CD19 and platelet marker CD41a [19]. Fenouillet et al. measured thiobarbituric acid reactive substances (TBARS) and found that there were significant differences in this product in ME/CFS patients compared to HC at rest [34]. During exercise, TBARS increased in ME/CFS patients and positively correlated with CD26-expression and negatively correlated with health-related quality of life [34].

Circulating hormone or peptide levels were investigated in five studies [35, 63, 70, 94, 101]. Shishioh-Ikejima et al. measured differences in alpha-melanocyte-stimulating hormone concentrations between ME/CFS patients and HC [94]. ME/CFS patients had significantly higher levels of alpha-melanocyte stimulating hormone; however, this was negatively associated with duration of illness [94]. Maes et al. found significantly lower serum dehydroepiandrosterone-sulfate (DHEAS) in ME/CFS patients [63]. Growth/differentiation factor-15 (GDF15) was found to be significantly higher in severe ME/CFS patients compared with HC by Melvin et al. [70]. Circulating levels of GDF15 was consistent across two different time points in mild/moderate patients [70]. Stringer et al. reported that fatigue severity significantly correlated with leptin in ME/CFS patients [101]. Plasma neuropeptide Y (NPY) was significantly higher in ME/CFS patients compared to HC. NPY had significant associations with various subjective measures including perceived stress and depression levels [35].

### Literature reporting on neurological biomarkers

There were eight studies reporting on neurological changes in ME/CFS patients compared with HC (Additional File 2, Table S5) [69, 79, 86, 89, 90, 93, 106, 112]. Two studies reported on ventricular lactate and levels were significantly higher in ME/CFS patients compared with HC [69, 79]. Functional connectivity differences in ME/CFS patients compared with HC were described in two studies [90, 93]. Shan et al. described higher complexity in ME/CFS patients in the default mode network while performing a stroop task [93]. The posterior cingulate cortex showed more complex blood oxygenation level-dependent (BOLD) signals in both the resting state and during a task compared with HC [93]. Rayhan et al. found that ME/CFS patients had lower BOLD signals compared with HC; however, following exercise, there was an increase in spontaneous activity in the anterior node of the default mode network in ME/CFS patients. All other studies reported on different neurological parameters [90]. Thapaliya et al. found that there were no significant changes in axial and mean diffusivity between FC ME/CFS patients and HC [106]. However, differences were found in ICC ME/CFS patients compared with HC in the descending cortico-cerebellar tract in the midbrain and pons [106]. Zeineh et al. found that bilateral white matter volumes were significantly lower in ME/CFS patients [112]. White matter microstructure disruptions in the right arcuate fasciculus were also identified [112]. Okada et al. reported that grey matter volumes were significantly reduced in the bilateral prefrontal cortex in people with ME/CFS compared with HC [86]. Provenzano et al. used a machine learning methodology based on 10 functional magnetic resonance imaging regions including but not limited to putamen, inferior frontal gyrus, orbital and supramarginal gyrus which was able to effectively differentiate between ME/CFS patients and HC (Pre-exercise day 1: Sensitivity = 87.5%; Specificity = 76.9%; Accuracy = 80.9%; post-exercise day 2: Sensitivity = 76.9%; Specificity = 75%; Accuracy = 76.1%) [89].

### Literature reporting on ion channel biomarkers

Nine studies reported changes in TRP, nociceptor or adrenergic receptors (Additional File 2, Table S6) [23, 29, 52, 59, 60, 67, 68, 83, 111]. Six studies were previously mentioned in the genetic/epigenetic section. [52, 59, 60, 67, 68, 111].

Three studies reported on TRPM3 function in NK cells [23, 29, 83]. Nguyen et al.’s study reported lower surface expression of TRPM3 receptors in unstimulated CD56^bright^CD16^dim/–^ NK cells as well as no significant differences in Ca^2+^ influx into the cell [23]. In contrast, CD56^bright^CD16^dim/–^ NK cells stimulated with pregnenolone sulfate (PregS) have significantly higher Ca^2+^ influx in ME/CFS compared with HC [23]. Cabanas et al. found lower TRPM3 currents following PregS stimulation [23]. Ca^2+^ influx through TRPM3 was also significantly lower in ME/CFS as described by Eaton-Fitch et al. [29].

### Literature reporting on physical biomarkers

In total, nine studies used physical based biomarkers (Additional File 2, Table S7) [15, 31, 34, 38, 40, 75, 80, 81, 98]. Heart rate markers were reported in seven studies [15, 31, 38, 40, 80, 81, 98]. Heart rate variability (HRV) was measured in three studies [31, 38, 40]. Gao et al. found that there was significantly higher heart rate variability (HRV) in ME/CFS patients compared with HC at baseline; however, when exposed to a stress test, there was no significant differences in HRV between the groups [40]. In a study by Frith et al., HRV was significantly increased higher in ME/CFS patients compared to HC at rest while parasympathetic markers were significantly lower [38]. Escorihuela et al. also assessed HRV at rest and found ME/CFS patients had decreased intervals between consecutive heartbeats (RR) in addition to HRV time and frequency-domain parameters compared with HC [31]. There was a significant association between RR scores and self-reported fatigue. Nelson et al. reported that there was lower post-exercise heart rate recovery in ME/CFS patients compared with HC [80]. In an earlier publication by the same author, work rate at ventilatory threshold was found to be lower in ME/CFS patients by 6.3–9.8% compared to HC on day 2 of CPET [81]. Snell et al. conducted two replicate analyses measuring lower workload in ME/CFS patients compared to HC. Test two showed that at peak exercise and ventilatory or anaerobic threshold, ME/CFS had significantly lower workload; however, test one showed no significant differences [98]. Allen et al. reported that overall pulse-timing response to controlled standing across all sites was significantly reduced in ME/CFS patients [15].

Nacul et al. assessed hand-grip strength differences between ME/CFS patients and HC [75]. Mild/moderate ME/CFS patients had a significant reduction of hand-grip strength (HGS) to 10.5 kg, and severe ME/CFS patients were associated with a reduction of HGS to approximately 15.3 kg [75]. These parameters correlated with clinical parameters such as disease severity, fatigue and pain analogue scale and physical component summary [75].

### Quality analysis

Quality of each included publication was assessed using the JBI CACCCS. Full results and justification can be found in Additional File 3. Six studies appropriately addressed all quality criteria [29, 50, 56, 95, 106, 115]. All studies assessed outcomes in a standard, valid and reliable way for both ME/CFS patients and HC (CACCCS criteria item 8). All papers provided appropriate justification for tools used to measure outcomes [15–115]. Potential confounding factors were appropriately identified in 81.18% of the studies [15–20, 22–32, 34–40, 43, 45, 47–52, 55–64, 66–70, 72–81, 83–90, 92, 93, 95, 96, 99–101, 103–106, 108–112, 115]. In most cases where confounding factors were identified, the researchers were able to mitigate or control for them (80%) [15–20, 22–32, 34–40, 43, 45, 47–52, 55–64, 66–70, 72–81, 83–90, 92, 93, 95, 99–101, 104–106, 108–112, 115]. ME/CFS patients were appropriately matched with HC in 67.33% of the studies [15–17, 19, 23–25, 27–31, 36–38, 40–43, 46, 48, 50, 51, 53–56, 60–62, 64–70, 72, 73, 76–79, 82, 84, 85, 87, 89, 91, 94–96, 98, 99, 101–103, 105, 106, 108–113, 115]. The least addressed CACCCs item was checklist item 2: adequate matching of source population which was only achieved by 26.7% [21, 22, 25, 27–31, 43, 50, 51, 56–58, 64, 72, 76, 77, 83, 95, 102, 106, 107, 109, 110, 113, 115]. In total, 36.6% of studies selected appropriate statistical analysis [16, 18, 20, 23–25, 29, 35, 42, 44, 45, 47, 49–51, 55, 56, 66, 68, 94, 95, 99, 101, 104, 106, 114, 115]. There were 23 (22.8%) studies that included an exposure [15, 32, 34, 38, 40, 57–60, 70, 75, 80–82, 84, 89, 90, 93, 95, 99, 105, 111]. The exposure was measured in a standard and valid way in 100% of the studies. The exposure was also measured consistently across patients and HC [15, 32, 34, 38, 40, 57–60, 70, 75, 80–82, 84, 89, 90, 93, 95, 99, 105, 111] and the duration was sufficient to show an effect in 95.7% of studies [15, 32, 34, 38–40, 57–60, 70, 75, 80–82, 84, 89, 90, 93, 95, 98, 99, 105].

## Discussion

Although there is no consensus on a biomarker for ME/CFS, several markers have been suggested as potential candidates. The aim of this systematic review was to collate and appraise available literature on suggested biomarkers for ME/CFS.

This systematic review used “biomarker” keywords in the abstract and title to determine the search. Therefore, the only articles that were retrieved were limited to those that specifically contained the keywords as according to Cochrane’s guidelines [12]. Studies that did not contain the specific keywords but may investigate related biomarkers therefore may not have been included in the search. This study, however, gives an overview of potential biomarkers in the field of ME/CFS.

Females were more prominent across the studies where 76% of the ME/CFS patients and 72% of the HC were female. In literature, ME/CFS is more commonly reported in females [116]. There is limited understanding as to what mechanism is contributing to sex-specific differences. Only one included study investigated potential factors influencing female predominance in epidemiological datasets through analysis of lipid profiles in male and female ME/CFS patients [41]. Although this study found significant differences, these were against all HC not ME/CFS females; therefore, they did not appropriately control for sex-related differences [41].

Majority of the participants in this review are diagnosed according to FC [15, 19, 21, 22, 24, 28, 31, 35–42, 44, 45, 49, 50, 52, 53, 59, 61–69, 71, 78, 79, 83, 84, 86–89, 92–95, 98, 99, 101, 102, 105, 108, 111–115]. The predominance of the FC use in biomarker studies is a limitation as this criteria is broad in nature and has considerable overlap with other illnesses [6]. There was a shift in criteria used over time, where the much broader FC is phasing out in more recent studies for more stringent definitions CCC and ICC. This is due to the CCC now replacing FC as the internationally recognised definition for both clinical and research use [5]. Evidence of necessity for appropriately selected stringent definitions for detection of biomarkers can be shown in Thapaliya et al. who reported significant differences in patients diagnosed according to ICC criteria [106]. There were no significant clusters found between ME/CFS patients diagnosed according to FC [106]. This indicates that stratifying patients according to case criteria may allow for additional meaningful observations to be made.

ME/CFS is a multifactorial condition that is also often associated with comorbid conditions including, but not limited to, IBS and fibromyalgia. Presence of multiple comorbidities complicates determining a biomarker specific for ME/CFS. Where possible, stratification of patients with and without comorbidities may assist with further understanding ME/CFS-specific pathomechanisms. Furthermore, there are other illnesses that are currently only diagnosed with case criteria and have significant symptom and pathological overlap with ME/CFS such as Gulf War illness [90]. Inclusion of both patient groups supports further discernment of both pathologies. In one of the included studies in this review, some post COVID-19 patients met the definition of ME/CFS. Both post COVID-19 patients with and without ME/CFS demonstrated elevated levels of ET‑1 compared with HC [47]. Haffke et al. did not include ME/CFS patients that did not develop ME/CFS post COVID-19; however, whether ET-1 levels were associated with COVID-19 or related to ME/CFS too is unclear. With significant overlap, assessing similar ME/CFS markers in post COVID-19 condition is important to allow understanding of association or differentiation of the condition [47].

Appropriate selection criteria of HC are a necessary experimental consideration. There were two studies that investigated pathology in ME/CFS patients and blood-related relatives compared with HC [102, 109]. Importantly, Tokunaga et al. indicated significant differences in a number of non-classical monocytes between ME/CFS patients and non-affected blood relatives compared with non-related HC [109]. Sung et al. also reported that ME/CFS patients and non-affected blood relatives showed lower ADCC compared with non-affected HC [102]. These results show the importance of having well-matched non-related HC, as even asymptomatic relatives of those with ME/CFS may display physiological differences from non-related HC. Nineteen studies involved the recruitment of sedentary controls [31, 32, 35, 38, 48, 59, 65, 75, 80, 81, 84, 85, 89, 90, 98, 102, 105, 109]. Buford et al. highlighted that selection of sedentary controls introduces bias and may interfere with delineating between patients and healthy patients [117].

The majority of the biomarkers were blood-based. Blood-based biomarkers are recognised as accessible, direct and non-invasive, especially in at-risk populations. Importantly, condition of the blood or blood product is important to consider. Recovery of frozen lymphocyte samples resulted in reduced viability [73]. This result is supported by Mata et al. which found that frozen/overnight rested PBMC had higher antibody-dependent cell-mediated cytotoxicity and NK activity compared to fresh PBMC [118]. It is important that in vitro studies closely represent true in vivo biological processes.

There were 20 studies that investigated genetic and epigenetic changes in ME/CFS patients compared with HC [16, 18, 20, 21, 25, 27, 43, 52, 59, 60, 67, 68, 71, 82, 87, 88, 92, 108, 103, 111]. Many of these studies were independent, association studies and investigated changes in either different cell or tissue types; therefore, it was difficult to make comparisons. However, there is significant evidence of a genetic component of ME/CFS [16, 18, 20, 21, 25, 27, 43, 52, 59, 60, 67, 68, 71, 82, 87, 88, 92, 108, 111]. Only two studies investigated sequence differences through SNPs in ME/CFS patients compared with HC. These studies found significant SNP variants in TRP and mAChRs genes [67, 68]. Other ion channel abnormalities further downstream have been identified including increased expression of purinergic and cellular modulators, sensory and adrenergic receptors [52, 59, 60, 111]. MiR-21 was reported on in three studies. MiR-21 is a highly conserved, non-specific marker implicated in at least 29 diseases as reported by Jenike et al. [119]. Therefore, due to its association with many other diseases, miR-21 is not a suitable distinguishing biomarker. The emergence of whole genome sequencing may allow for a more comprehensive high-throughput characterisation of genome changes in ME/CFS patients compared with HC in contrast to association studies; however, there are significant cost ramifications for its use in laboratory or clinical settings [120].

Immune dysfunction was the most prevalent biomarker type investigated in the included publications. Among these studies supporting immune dysfunction, there were five that investigated NKCC [37, 65, 67, 107, 114]. NKCC and function were significantly reduced in ME/CFS patients compared with HC, this corroborates with findings in an individual systematic review on ME/CFS and NK cell cytotoxicity and phenotype by Eaton-Fitch et al. [121]. Cliff et al. found no difference in NKCC; however, it is difficult to compare these studies as there was significant differences in methodology including the use of freeze-thawed samples in contrast to freshly isolated NK cells [26]. Theorell et al. also found no differences in cytotoxic NK cell phenotype and function; however, these experiments were also conducted on freeze-thawed cells with 10% dimethyl sulfoxide (DMSO) [107]. DMSO has been shown to be toxic to cells at even lower doses < 10% [122]. Therefore, use of DMSO in higher concentrations is a significant limitation. These investigations were also only conducted on PBMCs and not directly on isolated lymphocytes. Brenu et al. conducted a longitudinal investigation on NKCC in ME/CFS patients compared to HC. Although the level of NKCC in ME/CFS fluctuates, NKCC is consistently reduced over time [114]. The consistency of NK cell pathology across multiple studies and over time as well as the accessibility of NK cells suggests that it is an effective model to investigate the pathomechanism of ME/CFS. There is insufficient evidence to suggest ME/CFS is an inflammatory or autoimmune disorder. Instead, there was evidence of both disrupted innate and acquired immune systems suggesting inflammation may be a secondary characteristic in response to cellular and immune dysregulation due to the cell’s reduced capacity to respond to the alert given off by inflammatory markers. van Eeden et al. reported that intensity of inflammation was associated with a decrease in NK cells in COVID-19 patients [123]. NK cell effector function was also significantly compromised in COVID-19 patients [123, 124].

Cabanas et al. and Eaton-Fitch et al. assessed potential physiological processes underlying NK cell dysfunction including measurement of TRPM3 ionic currents and Ca^2+^ influx in NK cells from ME/CFS patients compared to HC [23, 29]. These authors emphasised the importance of the second messenger Ca^2+^ in regulating NK cell function and maintenance of cellular homeostasis. In contrast, Nguyen et al. did not show any significant differences in Ca^2+^ influx through TRPM3 in ME/CFS patients compared to HC; however, this was potentially due to interfering small inward currents activated by PregS through the flow cytometry technique which was not apparent in the confocal microscopy approach [83]. Patch clamp offers a highly sensitive method to measure Ca^2+^ influx. While patch clamp is the gold standard for investigating ion channel physiology, it can be conducted complimentary with confocal live imaging. TRP channels are also stress-activated including infection. ME/CFS development post-infection was described in three of the included studies [47–49]. An epidemiology study by Chu et al. identified that approximately 64% of patients reported an infectious onset preceding development of ME/CFS [125].

Three studies described changes in mitochondria [73, 96, 113]. Most of the metabolomic studies were most associated with changes in amino acids and energy metabolism. There was evidence of mitochondrial physiological changes in ME/CFS patients compared with HC; however, there were very few corroborated findings across the included studies in this review [73, 96, 113]. A systematic review by Holden et al. investigated mitochondrial dysfunction in ME/CFS patients. This review found that there was minimal indication that there is disruption of mitochondrial genes [126]. Primary mitochondrial defects or mitochondrial disease are associated with multi-system manifestations and often result in respiratory failure and high morbidity [127]. To the best of the authors’ knowledge, respiratory failure has not been described in ME/CFS patients.

Neurological changes in ME/CFS were investigated in eight  of the included studies [69, 79, 86, 89, 90, 93, 106, 112]. The major characteristics that were found was that the activity of the brain of patients was more complex; however, functional connectivity was weaker in ME/CFS patients and this is associated with cognitive difficulties. Evidence of neuroinflammation through elevated ventricular lactate concentrations were observed; however, as shown by Natelson, there was no significant differences between ME/CFS, fibromyalgia and comorbid ME/CFS and fibromyalgia groups. Therefore, inflammation of the brain may not be a sufficient biomarker on its own as it is characteristic and non-differentiated between other overlapping conditions [79].

**Fig. 2:**
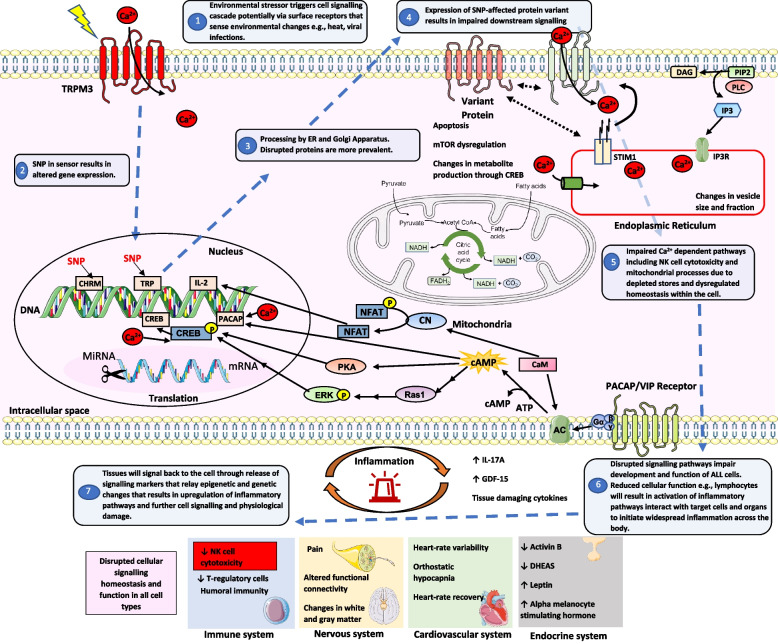
Postulated multidisciplinary pathway of ME/CFS. ME/CFS onset often occurs following an environmental trigger/s such as infection, trauma or chemical insult. ME/CFS is associated with genetic changes including SNPs in TRP and CHRM that are critical in cell signalling processes. In a two-step process, environmental triggers may result in upregulation of defected proteins that participate in these pathways and disruption of downstream signalling pathways involved in natural killer cell cytotoxicity and mitochondrial regulation. This can either directly affect different tissues and systems or indirectly through inflammatory pathways and cytokines. Cytokines and inflammation trigger epigenetic changes through mRNA or miRNA that further affect physiological function. Ca2^+^, calcium; CN, calcineurin, CaM, Camodulin, CHRM, cholinergic Receptor Muscarinic; cAMP, cyclic adenosine monophosphate, CREB, cyclic adenosine monophosphate response element-binding protein; DAG, diacylglycerol; DHEAs, dehydroepiandrosterone sulfate; DNA, Deoxyribonucleic acid; ERK, extracellular signal-regulated kinase; GDF15, growth/differentiation factor 15; IP3, inositol trisphosphate; IP3R, inositol trisphosphate receptor; IL, interleukin; mTOR, mammalian target of rapamycin; mRNA, messenger ribonucleic acid, MiRNA, micro ribonucleic acid; NFAT, nuclear factor of activated T D cells; PIP2, Phosphatidylinositol 4,5-bisphosphate; PLC, phospholipase C; PACAP, pituitary adenylate cyclase-activating peptide; STIM, stromal interaction molecule; TRP, Transient Receptor Potential; TRPM3, Transient Receptor Potential Melastatin 3; VIP, vasoactive intestinal peptide

Many of these biomarkers were studied in isolation but may be part of a complex multidisciplinary process as displayed by some of the overlap between observations made and extensive crosstalk between each system. There is evidence of widespread genetic, immune, neurological, mitochondrial and endocrine differences in ME/CFS compared with HC. Genetic abnormalities lead physiological disruption at the cellular and tissue level. Potential linking pathways have been described in Fig. 2. Some studies also showed multiple layers of evidence through use of different techniques to validate the same finding, for example TRPM3 dysfunction was demonstrated in various ways including genetic SNPs, genotypes, cell electrophysiology, and Ca^2+^ influx [9, 18, 61, 62, 77].

Use of exercise to promote PEM was commonly used in some studies. There are significant limitations to use of exercise to stimulate and measure biological changes in ME/CFS patients. The studies also do not cater for potential delays in onset of PEM where some patients reported symptoms presenting post 24 h. In many cases, inducing PEM through exercise requires specialised equipment and transport of patients to specific locations in order to collect data and many patients are unable to participate due to the severity of their illness. There is also a trade-off for specificity in all studies, for example Frith et al. had a sensitivity of 77% but a specificity of 53% [38]. Many ME/CFS patients will experience exercise intolerance but not everyone with exercise intolerance has ME/CFS. Exercise testing fell under the complex and indirect biomarker category as it required specialised equipment that is not available in most settings and has severe, long-term consequences to patients for an indirect measure of ME/CFS.

Most of these biomarkers fall under “moderate to difficult-to-detect” and requires extensive multi-step laboratory experiments by trained scientists in order to make these observations. Considerations for translation of the biomarker from laboratory to more automated high-throughput industry technology are critical. A rigorous approach is required for biomarkers to enter into the clinical stages of development [128]. Byrnes and Weigl described the importance of a biomarker’s ability to identify a disease-causing agent [10]. There were limited studies that were labelled “primary” as although onset from infectious agent have been described, there are several pathogens that have been attributed to the onset of ME/CFS [10]. Cytokines and circulatory markers such as hormones were investigated in many of the included studies and are “tertiary” biomarkers. These markers are produced via an immune response and are transient or variable and therefore often unstable as diagnostic targets. Two studies had “primary” markers that identified a disease-causing agent [48, 97]. Onset of ME/CFS through various infectious agents have been described and use of these markers provide valuable insight on mechanisms underlying different onsets, but are limited as a diagnostic tool [125].

Integration of machine learning algorithms with the development of biomarkers will allow to assess a model for classification and feature selection. With machine learning, you can select multiple strong candidates for ME/CFS and train the software to recognise these patterns and appropriately classify patients and HC with great sensitivity, specificity and accuracy as described by Missailidis et al. [73]. In this study, the authors combined all three assays: frozen lymphocyte death rate, lymphoblast mitochondrial dysfunction, and lymphoblast Target Of Rapamycin Complex 1 (TORC1) signalling which on their own were not outstanding discriminators; however, combining them gave an accuracy of 95% in determining ME/CFS patients from HC and a sensitivity of 97% and a specificity of 100% [73].

Stability is an important component when considering biomarkers. In some of the biomarkers investigated, changes in certain parameters were influenced by illness severity, illness duration, and onset patterns such as infectious onset and time [18, 70, 72, 74, 75, 100]. Although these investigations are useful in understanding the pathomechanism occurring at different stages and presentations as well as stratification of patients, a diagnostic biomarker should be universal and applicable despite these factors. Incorporation of a mixed disease population representative of the ME/CFS community in cases where illness is heterogenous is important. Longitudinal investigations are critical for understanding the stability of the biomarker over time [114]. There were only two studies that investigated biomarkers longitudinally, one that measured NK cell subtypes iNKT and NKG2D and one that investigated NKCC over time [114, 115].

Many of the studies investigated were standalone studies with insufficient or proof-of-concept sample sizes. It is difficult to compare studies of the same marker as their methodology varied significantly including cell or tissue type. Some studies implemented two phases, discovery and validation phase and this process was effective in demonstrating test-test reproducibility. In some cases, the sample sizes are relatively small; however, reproducibility is also demonstrated through assessing many individual cells across multiple samples and shows robustness between and within samples [23, 29, 83].

### Quality analysis

Levels of quality varied across the included papers, with six studies appropriately addressing all quality criteria [29, 50, 56, 95, 106, 115]. All studies assessed outcomes in a standard, valid and reliable way for both ME/CFS patients and HC (CACCCS criteria item 8); this is often through use of validated experimental tools. All papers provided appropriate justification for tools used to measure outcomes including, but not limited to, flow cytometry to heart rate monitors [15–115]. ME/CFS patients and HC were matched in various ways most predominantly age and sex, however, also through BMI and race. In most cases where a confounding factor was identified, the researchers were able to mitigate or control for potential confounding variables using a variety of methods including exclusion, a washout period for medication or statistical adjustment for cofactors [15–20, 22–32, 34–40, 43, 45, 47–52, 55–64, 66–70, 72–81, 83–90, 92, 93, 95, 99–101, 104–106, 108–112, 115]. The least addressed CACCCs item was checklist item 2: adequate matching of source population [21, 22, 25, 27–31, 43, 50, 51, 56–58, 64, 72, 76, 77, 83, 95, 102, 106, 107, 109, 110, 113, 115]. Appropriate matching of sociodemographic characteristics through recruitment of matched participants from similar geographical regions is critical to eliminate potential sources of bias. Additionally, inclusion of power-based sample sizes, normality testing and adjustments for multiple comparisons is also necessary to ensure accurate and representative statistical outputs.

## Conclusions

There is currently no consensus on a biomarker for ME/CFS; however, there is significant body of biological evidence demonstrating that ME/CFS results in widespread immunological disruption. Effective biomarkers are accessible and have strong sensitivity, specificity and selectivity to detect disease targets. Additionally, having multiple layers of evidence such as genetic and physiological high test-test reproducibility of results is paramount. The included studies ranged in efficacy, quality, and potential to be developed into a diagnostic biomarker. This review also corroborates the use of NK cells as a suitable model to investigate the pathomechanism of this illness due to the consistency observed over time. This systematic review identifies potential associations between the findings and highlights that these systems do not work independently and rather they could be part of a complex integrative network. The heterogeneity shown across many of the included studies highlights the need for multidisciplinary research and uniform protocols in ME/CFS biomarker research.

## Supplementary Information

**Additional file 1.** Database search terms.

**Additional file 2.** Study and participant results. **Table S1.** Genetic biomarkers; **Table S2.** Immunological biomarkers; **Table S3.** Metabolomics/ mitochondrial/ microbiome biomarkers; **Table S4.** Endovascular/circulatory biomarkers; **Table S5.** Neurological biomarkers; **Table S6.** Ion channel biomarkers; **Table S7.** Physical biomarkers. 

**Additional file 3.** JBI quality assessment table and descriptions

## Data Availability

All data generated or analysed during this study are included in this published article [and its Additional files].

## Abbreviations

ADCC: Antibody-dependent cell-mediated cytotoxicity
AUC: Area under the curve
BAP: Biological antioxidant potential
BMI: Body mass index
BOLD: Blood oxygenation level dependent
Ca^2+^: Calcium
CCC: Canadian Consensus Criteria
DHEAS: Dehydroepiandrosterone-sulfate
DNA: Deoxyribonucleic acid
d-ROMs: Diacron reactive oxygen metabolites
ET-1: Endothelin 1
EV: Extracellular vesicles
FC: Fukuda Criteria
GDF15: Growth/differentiation factor-15
HC: Healthy control
HGS: Hand-grip strength
HRV: Heart rate variability
IBS: Irritable bowel syndrome
ICC: International Consensus Criteria
IL: Interleukin
IOMC: Institute of Medicine Criteria
JBI CACCCS: Joanna Briggs Institute Critical Appraisal Checklist for Case Control Studies
mAChM3: Muscarinic Acetylcholine Receptor M3
ME/CFS: Myalgic encephalomyelitis/chronic fatigue syndrome
MeSH: Medical Subject Headings
miRNA: Micro ribonucleic acid
mRNA: Messenger ribonucleic acid
NK: Natural killer
NKCC: Natural killer cell cytotoxicity
NPY: Neuropeptide Y
PBMC: Peripheral blood mononuclear cells
PEM: Post-exertional malaise
PENE: Post-exertional neuroimmune exhaustion
PRISMA: Preferred Reporting Items for Systematic Reviews and Meta-analyses
ROC: Receiver operator characteristics
SNP: Single-nucleotide polymorphism
TORC1: Target Of Rapamycin Complex 1
TRPM3: Transient Receptor Potential Melastatin 3
