# Supplementary material for: Biomarkers for myalgic encephalomyelitis/chronic fatigue syndrome (ME/CFS): a systematic review
Source: BMC Med. 2023 May 24;21:189. doi: 10.1186/s12916-023-02893-9 (PMC10206551; doi:10.1186/s12916-023-02893-9)
Supplement: Supplementary file 1 — Additional file 1. Database search terms. [file 12916_2023_2893_MOESM1_ESM.docx]

**Additional file 1.** Database search terms

| **Pubmed** | (("Chronic Fatigue Syndrome"[title/abstract] OR "Myalgic Encephalomyelitis"[title/abstract] OR “Encephalomyelitis, Myalgic”[title/abstract] OR “Chronic Fatigue Syndromes”[title/abstract] OR “Fatigue Syndromes, Chronic”[title/abstract] OR “Chronic Fatigue-Fibromyalgia Syndrome”[title/abstract] OR “Chronic Fatigue Fibromyalgia Syndrome”[title/abstract] OR “Chronic Fatigue Fibromyalgia Syndromes”[title/abstract] OR “Fatigue-Fibromyalgia Syndrome, Chronic”[title/abstract] OR “Fatigue-Fibromyalgia Syndromes, Chronic”[title/abstract] OR “Postviral Fatigue Syndrome”[title/abstract] OR “Infectious Mononucleosis-Like Syndrome, Chronic”[title/abstract] OR “Infectious Mononucleosis Like Syndrome, Chronic”[title/abstract] OR “Royal Free Disease”[title/abstract] OR “Chronic Fatigue and Immune Dysfunction Syndrome”[title/abstract] OR “Chronic Fatigue Disorder”[title/abstract] OR “Chronic Fatigue Disorders”[title/abstract] OR “Fatigue Disorder, Chronic”[title/abstract] OR “Fatigue Disorders, Chronic”[title/abstract] OR "systemic exertion intolerance"[title/abstract] OR “Fatigue Syndrome, Postviral”[title/abstract] OR “Fatigue Syndromes, Postviral”[title/abstract] OR “Postviral Fatigue Syndromes”[title/abstract])) AND ((“Biomarkers”[title/abstract] OR “Marker, Biological”[title/abstract] OR “Biological Marker”[title/abstract] OR “Biologic Marker”[title/abstract] OR “Marker, Biologic”[title/abstract] OR “Biological Markers”[title/abstract] OR “Biologic Markers”[title/abstract] OR “Markers, Biologic”[title/abstract] OR “Biomarker”[title/abstract] OR “Markers, Biological”[title/abstract] OR “Markers, Immunologic”[title/abstract] OR “Immune Markers”[title/abstract] OR “Markers, Immune”[title/abstract] OR “Marker, Immunologic”[title/abstract] OR “Immunologic Markers”[title/abstract] OR “Immune Marker”[title/abstract] OR “Marker, Immune”[title/abstract] OR “Immunologic Marker”[title/abstract] OR “Serum Markers”[title/abstract] OR “Markers, Serum”[title/abstract] OR “Marker, Serum”[title/abstract] OR “Serum Marker”[title/abstract] OR “Surrogate Endpoints”[title/abstract] OR “Surrogate End Point”[title/abstract] OR “End Point, Surrogate”[title/abstract] OR “Surrogate End Points”[title/abstract] OR “Surrogate Endpoint”[title/abstract] OR “Endpoint, Surrogate”[title/abstract] OR “Markers, Clinical”[title/abstract] OR “Clinical Markers”[title/abstract] OR “Clinical Marker”[title/abstract] OR “Marker, Clinical”[title/abstract] OR “Viral Markers”[title/abstract] OR “Markers, Viral”[title/abstract] OR “Viral Marker”[title/abstract] OR “Marker, Viral”[title/abstract] OR “Biochemical Marker”[title/abstract] OR “Markers, Biochemical”[title/abstract] OR “Marker, Biochemical”[title/abstract] OR “Biochemical Markers”[title/abstract] OR “Markers, Laboratory”[title/abstract] OR “Laboratory Markers”[title/abstract] OR “Laboratory Marker”[title/abstract] OR “Marker, Laboratory”[title/abstract] OR “Surrogate Markers”[title/abstract] OR “Markers, Surrogate”[title/abstract] OR “Marker, Surrogate”[title/abstract] OR “Surrogate Marker”[title/abstract])) |
| --- | --- |
| **Embase** | (("Chronic Fatigue Syndrome":ab,ti OR "Myalgic Encephalomyelitis":ab,ti OR “Encephalomyelitis, Myalgic”:ab,ti OR “Chronic Fatigue Syndromes”:ab,ti OR “Fatigue Syndromes, Chronic”:ab,ti OR “Chronic Fatigue-Fibromyalgia Syndrome”:ab,ti OR “Chronic Fatigue Fibromyalgia Syndrome”:ab,ti OR “Chronic Fatigue Fibromyalgia Syndromes”:ab,ti OR “Fatigue-Fibromyalgia Syndrome, Chronic”:ab,ti OR “Fatigue-Fibromyalgia Syndromes, Chronic”:ab,ti OR “Postviral Fatigue Syndrome”:ab,ti OR “Infectious Mononucleosis-Like Syndrome, Chronic”:ab,ti OR “Infectious Mononucleosis Like Syndrome, Chronic”:ab,ti OR “Royal Free Disease”:ab,ti OR “Chronic Fatigue and Immune Dysfunction Syndrome”:ab,ti OR “Chronic Fatigue Disorder”:ab,ti OR “Chronic Fatigue Disorders”:ab,ti OR “Fatigue Disorder, Chronic”:ab,ti OR “Fatigue Disorders, Chronic”:ab,ti OR "systemic exertion intolerance":ab,ti OR “Fatigue Syndrome, Postviral”:ab,ti OR “Fatigue Syndromes, Postviral”:ab,ti OR “Postviral Fatigue Syndromes”:ab,ti)) AND ((“Biomarkers”:ab,ti OR “Marker, Biological”:ab,ti OR “Biological Marker”:ab,ti OR “Biologic Marker”:ab,ti OR “Marker, Biologic”:ab,ti OR “Biological Markers”:ab,ti OR “Biologic Markers”:ab,ti OR “Markers, Biologic”:ab,ti OR “Biomarker”:ab,ti OR “Markers, Biological”:ab,ti OR “Markers, Immunologic”:ab,ti OR “Immune Markers”:ab,ti OR “Markers, Immune”:ab,ti OR “Marker, Immunologic”:ab,ti OR “Immunologic Markers”:ab,ti OR “Immune Marker”:ab,ti OR “Marker, Immune”:ab,ti OR “Immunologic Marker”:ab,ti OR “Serum Markers”:ab,ti OR “Markers, Serum”:ab,ti OR “Marker, Serum”:ab,ti OR “Serum Marker”:ab,ti OR “Surrogate Endpoints”:ab,ti OR “Surrogate End Point”:ab,ti OR “End Point, Surrogate”:ab,ti OR “Surrogate End Points”:ab,ti OR “Surrogate Endpoint”:ab,ti OR “Endpoint, Surrogate”:ab,ti OR “Markers, Clinical”:ab,ti OR “Clinical Markers”:ab,ti OR “Clinical Marker”:ab,ti OR “Marker, Clinical”:ab,ti OR “Viral Markers”:ab,ti OR “Markers, Viral”:ab,ti OR “Viral Marker”:ab,ti OR “Marker, Viral”:ab,ti OR “Biochemical Marker”:ab,ti OR “Markers, Biochemical”:ab,ti OR “Marker, Biochemical”:ab,ti OR “Biochemical Markers”:ab,ti OR “Markers, Laboratory”:ab,ti OR “Laboratory Markers”:ab,ti OR “Laboratory Marker”:ab,ti OR “Marker, Laboratory”:ab,ti OR “Surrogate Markers”:ab,ti OR “Markers, Surrogate”:ab,ti OR “Marker, Surrogate”:ab,ti OR “Surrogate Marker”:ab,ti)) |
| **Scopus** | ((TITLE-ABS("Chronic Fatigue Syndrome") OR TITLE-ABS("Myalgic Encephalomyelitis") OR TITLE-ABS(“Encephalomyelitis, Myalgic”) OR TITLE-ABS(“Chronic Fatigue Syndromes”) OR TITLE-ABS(“Fatigue Syndromes, Chronic”) OR TITLE-ABS(“Chronic Fatigue-Fibromyalgia Syndrome”) OR TITLE-ABS(“Chronic Fatigue Fibromyalgia Syndrome”) OR TITLE-ABS(“Chronic Fatigue Fibromyalgia Syndromes”) OR TITLE-ABS(“Fatigue-Fibromyalgia Syndrome, Chronic”) OR TITLE-ABS(“Fatigue-Fibromyalgia Syndromes, Chronic”) OR TITLE-ABS(“Postviral Fatigue Syndrome”) OR TITLE-ABS(“Infectious Mononucleosis-Like Syndrome, Chronic”) OR TITLE-ABS(“Infectious Mononucleosis Like Syndrome, Chronic”) OR TITLE-ABS(“Royal Free Disease”) OR TITLE-ABS(“Chronic Fatigue and Immune Dysfunction Syndrome”) OR TITLE-ABS(“Chronic Fatigue Disorder”) OR TITLE-ABS(“Chronic Fatigue Disorders”) OR TITLE-ABS(“Fatigue Disorder, Chronic”) OR TITLE-ABS(“Fatigue Disorders, Chronic”) OR TITLE-ABS("systemic exertion intolerance") OR TITLE-ABS(“Fatigue Syndrome, Postviral”) OR TITLE-ABS(“Fatigue Syndromes, Postviral”) OR TITLE-ABS(“Postviral Fatigue Syndromes”))) AND ((TITLE-ABS(“Biomarkers”) OR TITLE-ABS(“Marker, Biological”) OR TITLE-ABS(“Biological Marker”) OR TITLE-ABS(“Biologic Marker”) OR TITLE-ABS(“Marker, Biologic”) OR TITLE-ABS(“Biological Markers”) OR TITLE-ABS(“Biologic Markers”) OR TITLE-ABS(“Markers, Biologic”) OR TITLE-ABS(“Biomarker”) OR TITLE-ABS(“Markers, Biological”) OR TITLE-ABS(“Markers, Immunologic”) OR TITLE-ABS(“Immune Markers”) OR TITLE-ABS(“Markers, Immune”) OR TITLE-ABS(“Marker, Immunologic”) OR TITLE-ABS(“Immunologic Markers”) OR TITLE-ABS(“Immune Marker”) OR TITLE-ABS(“Marker, Immune”) OR TITLE-ABS(“Immunologic Marker”) OR TITLE-ABS(“Serum Markers”) OR TITLE-ABS(“Markers, Serum”) OR TITLE-ABS(“Marker, Serum”) OR TITLE-ABS(“Serum Marker”) OR TITLE-ABS(“Surrogate Endpoints”) OR TITLE-ABS(“Surrogate End Point”) OR TITLE-ABS(“End Point, Surrogate”) OR TITLE-ABS(“Surrogate End Points”) OR TITLE-ABS(“Surrogate Endpoint”) OR TITLE-ABS(“Endpoint, Surrogate”) OR TITLE-ABS(“Markers, Clinical”) OR TITLE-ABS(“Clinical Markers”) OR TITLE-ABS(“Clinical Marker”) OR TITLE-ABS(“Marker, Clinical”) OR TITLE-ABS(“Viral Markers”) OR TITLE-ABS(“Markers, Viral”) OR TITLE-ABS(“Viral Marker”) OR TITLE-ABS(“Marker, Viral”) OR TITLE-ABS(“Biochemical Marker”) OR TITLE-ABS(“Markers, Biochemical”) OR TITLE-ABS(“Marker, Biochemical”) OR TITLE-ABS(“Biochemical Markers”) OR TITLE-ABS(“Markers, Laboratory”) OR TITLE-ABS(“Laboratory Markers”) OR TITLE-ABS(“Laboratory Marker”) OR TITLE-ABS(“Marker, Laboratory”) OR TITLE-ABS(“Surrogate Markers”) OR TITLE-ABS(“Markers, Surrogate”) OR TITLE-ABS(“Marker, Surrogate”) OR TITLE-ABS(“Surrogate Marker”)) |
