# Supplementary material for: Biomarkers for myalgic encephalomyelitis/chronic fatigue syndrome (ME/CFS): a systematic review
Source: BMC Med. 2023 May 24;21:189. doi: 10.1186/s12916-023-02893-9 (PMC10206551; doi:10.1186/s12916-023-02893-9)
Supplement: Supplementary file 2 — Additional file 2. Study and participant results. Table S1. Genetic biomarkers; Table S2. Immunological biomarkers; Table S3. Metabolomics/ mitochondrial/ microbiome biomarkers; Table S4. Endovascular/circulatory biomarkers; Table S5. Neurological biomarkers; Table S6. Ion channel biomarkers; Table S7. Physical biomarkers. [file 12916_2023_2893_MOESM2_ESM.docx]

| **Additional file 2.** Study and participant results | | | | | | | | | | | | | | | | | |
| --- | --- | --- | --- | --- | --- | --- | --- | --- | --- | --- | --- | --- | --- | --- | --- | --- | --- |
| **Table S1. Genetic biomarkers** | | | | | | | | | | | | | | | | | |
| **Author (year)** | **Study type** | **Criteria** | **Sample size** | | **Age (years, mean ± SD)** | | **Sex (female)** | | **BMI (kg/m^2^)** | | **Illness duration (years)** | **Biomarker** | **Classification** | **Selectivity** | **Ease of detection** | **AUC; Sensitivity; Specificity** | **Findings** |
|  |  |  | **ME/CFS** | **HC** | **ME/CFS** | **HC** | **ME/CFS** | **HC** | **ME/CFS** | **HC** | **ME/CFS** |  |  |  |  |  |  |
| Blauensteiner et al (2021) (18) | Observational case-control | CCC  Fukuda | 30 | 29 | ME/CFS mild/ moderate: 26 (23-28)  ME/CFS severely affected : 22 (20-27) | 43 (32-48)* | ME/CFS mild/moderate: 71%  ME/CFS severely affected: 80% | 41% | ME/CFS mm: 26 (23 - 28)*  ME/CFS Sa: 22 (20-27)* | 24 (22-30)* | NR | microRNAs | Blood | Secondary | Moderate | NR | Sirt1/eNOS modulating microRNAs: miR‑21, miR‑34a, miR‑92a, miR‑126, and miR‑200c are significantly increased in ME/CFS patients compared to HC (p = 0.006). |
| Brenu et al (2012A) (21) | Observational case- control | Fukuda | 28 | 28 | 41.8 ± 9.6 | 5.3 ± 11.7 | 82.1% | 71.4% | NR | NR | NR | Cytotoxic lymphocyte microRNAs | Blood | Tertiary | Moderate | NR | Expression of miR-21, miR-17-5p,miR-10a,miR-103,miR-152,miR-146a,miR-106,miR-223and miR-191 was significantly lower in NK cells belonging to ME/CFS patients compared to HC (p < 0.05). MiR-21 was also lower in CD8^+^ T cells of ME/CFS patients (p < 0.05). |
| Brenu et al (2014) (20) | Observational case-control | Fukuda | 20 | 20 | 44.5 ± 6.0 | 47.3 ± 6.7 | NR | NR | NR | NR | NR | Plasma microRNAs | Blood | Tertiary | Moderate | NR | Three miRNAs (hsa-miR-127-3p, hsa-miR-142-5pand hsa-miR-143-3p that were differentially expressed and significantly upregulated in ME/CFS based on RT-qPCR (p < 0.05). |
| Chacko et al (2016) (25) | Observational case- control | ICC | Moderate: 11  Severe: 12 | 11 | Moderate: 54.9 ± 10.3  Severe: 47.5 ± 8.0 | 50.0 ± 12. | Moderate: 83.3%  Severe: 75% | 72.5% | NR | NR | NR | Protein Kinase | Blood | Secondary | Moderate | NR | There were 92 differentially expressed protein kinase genes: 37 genes that were significantly upregulated and 55 genes that were significantly downregulated in severe ME/CFS patients compared to HC (p < 0.001). |
| de Vega et al (2017) (27) | Observational case- control | CCC  Fukuda | 49 | 25 | 49.4 ± 1.9 | 51.1 ± 2.7 | 100% | 100% | 23.3 ± 0.5 | 23.4 ± 0.6 | NR | DNA methylome | Blood | Tertiary | Complex | NR | There were 12, 608 differentially methylated sites in ME/CFS patients compared to HC. Glucocorticoid sensitivity in ME/CFS patients was associated with differential methylation at 13 loci (p≤0.05). |
| Gow et al (2009) (43) | Observational case-control | Fukuda | 8 | 7 | 36 (18-54) | 34 (22-58) | 0% | 0% | NR | NR | 4.5* | mRNA | Blood | Secondary | Moderate | NR | There were 366 differentially expressed genes between ME/CFS patients and HC (p < 0.05) |
| Iacob et al (2016) (52) | Observational case-control | Fukuda | ME/CFS: 33  ME/CFS + FMS: 79 | 61 | ME/CFS: 44.76 ± 13.9  ME/CFS + FMS: 48.16 ± 13.4 | 40.86 ± 14.5 | ME/CFS: 42.4%  ME/CFS + FMS: 82.3% | 54.1% | NR | NR | NR | Gene expression | Blood | Secondary | Moderate | NR | Greater gene expression of purinergic and cellular modulators (p= 0.04) and nociception and stress mediators were positively associated with ME/CFS (p = 0.02). |
| Light et al (2009) (60) | Observational case-control | CCC  Fukuda | 48 | 49 | 41.8 ± 1.9 | 42.0 ± 1.9 | 68.8% | 59.1% | 26.2 ± 0.8 | 23.9 ± 0.7 | NR | Gene/mRNA expression | Blood | Secondary | Moderate | Unclear | Post exercise, sensory and adrenergic receptors and cytokine gene transcription was increased for 48 hours (p < 0.05). In 29% of patients with ME/CFS adrenergica-2A receptor’s transcription was decreased (p < 0.05). |
| Light et al (2012) (59) | Observational case-control | Fukuda | 19 | 15 | 42.2 ± 2.7 | 35.6 ± 3.0 | 78.9% | 73.0% | 27.0 ± 1.18 | 22.9 ± 0.73 | NR | mRNA expression | Blood | Secondary | Moderate | AUC: 0.91; Sensitivity: 0.91; specificity: 0.77. | Gene expression for the following: ASIC3, P2X4, and P2X5, for sensory receptors alpha-2A, beta-1, beta-2, and COMT and IS genes for IL10 and TLR4 was increased in ME/CFS for up to 0.5 to 48 hours (p < 0.05). |
| Marshall-Gradisnik et al (2016A) (67) | Observational case-control | Fukuda | 39 | 30 | 51.69±2 | 47.60±2.39 | 71.79% | 56.67% | NR | NR | NR | Genetic SNPs TRP channels | Blood | Secondary | Moderate | NR | ME/CFS patients had significantly lower NK cell lysis of target cells compared to HC. There were eleven SNPs identified in TRP ion channel genes (TRPC4, TRPC2, TRPM3, and TRPM8). Five of these SNPs were associated with TRPM3 (p < 0.05). There were 14 SNPs associated with nicotinic and muscarinic genes including: CHRNA2, CHRNA3, CHRNB4, CHRNA5, and CHRNE (p < 0.05). There were six SNPs associated with CHRNA3. Sixteen genotypes were identified from the SNPs (p < 0.05). |
| Marshall- Gradisnik et al (2016B) (68) | Observational  Case-control | Fukuda | 11 | 11 | 31.82±5.50 | 33.91±5.06 | 72.7% | 63.6% | NR | NR | NR | SNPs TRP and Acetylcholine receptors | Blood | Secondary | Moderate | NR | There were 78 SNPs identified in nicotinic and muscarinic acetylcholine receptor genes in B cells from ME/CFS patients compared to HC. Thirty- five of these SNPs were identified in mAChM3. Other SNPs that were identified included: nAChR delta, nAChR alpha 9, TRPV2, TRPM3, TRPM4, mAChRM3 2, and mAChRM5 (p < 0.05). From these SNPs, nine genotypes were identified (p <0.05) |
| Metselaar et al (2021) (71) | Observational case- control | Fukuda | 93 | 25 | NR | NR | NR | NR | NR | NR | NR | mRNA expression | Blood | Secondary | Moderate | REFS AUC: 0.92  REFS Validated AUC: 0.97  AUC CpGs: 0.97 | Recursive ensemble feature selection (REFS) found 23 genes that were able to significantly differentiate between ME/CFS patients and HC. From these genes, 48 CpGs were predictive of ME/CFS (r2 = 0.960, p < 0.0001). |
| Nepotchatykh et al (2020) (82) | Observational case- control | CCC | Discovery cohort: 11  Replication cohort: 32 | Discovery cohort: 8  Replication cohort: 17 | Discovery cohort: 58 ± 2.3  Replication cohort: 49.2 ± 2.1 | Discovery cohort: 58 ± 4  Replication cohort: 49.8 ± 2.2 | Discovery cohort: 81%  Replication cohort: 56.25% | Discovery cohort: 62.5%  Replication cohort: 64.7% | Discovery cohort: 23.1 ± 1.2  Replication cohort: 25.1 ± 0.8 | Discovery cohort: 24.0 ± 1.4  Replication cohort: 25.5 ± 1.4 | Discovery cohort: 17 ± 2.0  Replication cohort: 14.6 ± 2.1 | microRNAs | Blood | Secondary | Moderate | NR | ME/CFS patients had specific microRNA expression signatures following PEM induction. These microRNA expression signatures were associated with symptom severity (p < 0.05). |
| Petty et al (2016) (87) | Observational case- control | Fukuda | Microarray cohort: 15  Fractionated lymphocyte cohort: 20 | Microarray cohort: 30  Fractionated lymphocyte cohort: 20 | Microarray cohort:  Fractionated lymphocyte cohort: | Microarray cohort:  Fractionated lymphocyte cohort: | Microarray cohort: 80%  Fractionated lymphocyte cohort: 80.0% | Microarray cohort: 73.0%  Fractionated lymphocyte cohort: 80.0% | NR | NR | 6.8 | mRNAs | Blood | Secondary | Moderate | AUC: 0.71-0.78 | Thirty-four differentially expressed, upregulated miRNA were found. Four of these miRNA markers were also upregulated by qRT-PCR, (p ≤ 0.05). Fractionization of PBMC samples found the most profound changes occurred in NK cells (p ≤ 0.05). |
| Powell et al (2003) (88) | Observational case- control | Fukuda | 7 | 4 | 18-50 | 18-50 | 28.6% | 50.0% | NR | NR | NR | mRNA | Blood | Secondary | Moderate | NR | Using differential display, 12 short expressed sequence tags were found to be overexpressed in activated lymphocytes. Elevated expression levels for seven genes were confirmed using TAQman quantitative PCR (p < 0.05) |
| Saiki et al (2008) (92) | Observational case- control | Fukuda | 11 | 20 | 33.4 ± 9.4 | NR | 63.3% | NR | 20.3 ± 2.2 | NR | NR | mRNA | Blood | Secondary | Moderate | NR | mRNA levels were significantly different in ME/CFS patients compared to HC in 12 genes. Quantitative real-time PCR validated these changes in nine of these genes. These genes encoded: granzyme in activated T or natural killer cells, energy regulators (ATP5J2,COX5B, and DBI), proteasome subunits (PSMA3 and PSMA4), putative protein kinase c inhibitor (HINT), GTPase (ARHC), and signal transducers and activators of transcription 5A (STAT5A) (p < 0.05). In a follow up microarray investigation: 9 genes were found to effectively classify 79% of ME/CFS patients when compared to other non-ME/CFS patients that experience fatigue. Real- time PCR, in contrast, was able to effectively classify 94% of ME/CFS patients. |
| Tiev et al (2003) (108) | Observational case- control | Fukuda | 11 | 14 | 43.2 ± 13.8 | 39.1 ± 11.6 | 54.54% | 71.43% | NR | NR | 7.6 ± 6.6 | RNase L | Blood | Secondary | Moderate | Sensitivity = 91%; Specificity = 71% | Presence of RNase L isoform ratio levels in PBMC’s belonging to ME/CFS patients was significantly higher compared to HC (no p-value) |
| White et al (2012) (111) | Observational case- control | Fukuda | 22 | 23 | 40.8 ± 2.0 | 38.7 ± 2.4 | 86.36% | 82.61% | 25.0 ± 1.2 | 24.3 ± 0.8 | 7 ± 1.4 | mRNA in metabolite- detecting receptors | Blood | Secondary | Moderate | NR | ME/CFS patients had elevated mRNA in P2X4, TRPV1, CD14 and all adrenergic receptors compared to HC post-exercise (p = < 0.05). Those with ME/CFS and comorbid Fibromyalgia showed elevated mRNA in ASIC3 and P2X5 (p < 0.05). |

| **Table S2. Immunological biomarkers** | | | | | | | | | | | | | | | | | |
| --- | --- | --- | --- | --- | --- | --- | --- | --- | --- | --- | --- | --- | --- | --- | --- | --- | --- |
| **Author (date)** | **Study type** | **Criteria** | **Sample size** | | **Age (years, mean ± SD)** | | **Sex (female %)** | | **BMI (kg/m^2^)** | | **Illness duration (years)** | **Biomarker** | **Classification** | **Selectivity** | **Ease of detection** | **AUC; Sensitivity; Specificity** | **Findings** |
|  |  |  | **ME/CFS** | **HC** | **ME/CFS** | **HC** | **ME/CFS** | **HC** | **ME/CFS** | **HC** | **ME/CFS** |  |  |  |  |  |  |
| Brenu et al (2011) (22) | Observational case-control | Fukuda | 95 | 50 | 46.47 ± 11.7 | 41.9 ± 9.6 | 70.5% | 57.7% | NR | NR | NR | Cytokines | Blood | Tertiary | Complex | NR | ME.CFS patients had significant increases in IL-10, IFN-g, TNF-a,CD4^+^CD25^+^T cells, FoxP3 and VPACR2 expression compared to HC. Cytotoxic activity of NK cells, particularly, CD56^bright^NK cells were decreased in ME/CFS patients. Granzyme A and Granzyme K expression were reduced, and perforin levels were elevated in ME/CFS patients. Cytotoxic activity of CD8^+^ T cells and NK phenotypes were also decreased in patients (P≤0.05). |
| Brenu et al (2012B) (113) | Prospective  longitudinal linked panels | Fukuda | 65 | 21 | 47.2 ± 11.5 | 45.2 ± 9.3 | 75.4% | 66.7% | 24.4 ± 4.9 | 25.3 ± 5.5 | NR | NK cell cytotoxicity and cytokines | Blood | Tertiary | Complex | NR | NKCC was significantly decreased at all timepoints: baseline (T1), six months (T2) and 12 months (T3) in ME/CFS patients compared to HC (p < 0.05). Differences between each timepoint was also significantly different (p < 0.001). ME/CFS patients had lower CD56^bright^CD16^-^NK cells at timepoints T1 and T2 (p < 0.05). After mitogenic stimulation, there were significant increases in IL-10, IFN-y and TNF-a at T1. IL-10, IL-17A were significantly decreased at T2. IL-2 was increased at T3 in ME/CFS patients (p < 0.05). |
| Cliff et al (2019) (26) | Observational case-control | CCC  Fukuda | Moderate: 197  Severe: 54 | 107 | Moderate: 43 (34-52)  Severe: 46 (32-51) | 44 (32-52) | Moderate: 76.0%  Severe: 75.9% | 73.8% | <40 | NR <40 | NR | herpes virus serological status and T cell, B cell, NK cell and monocyte populations | Blood | Secondary | Moderate | NR | No significant differences were observed in NK cell numbers, subtype proportions and cell cytotoxicity. There was an increase in circulating MAIT cells in those with severe ME/CFS (p < 0.001). |
| Espinosa et al (2019) (33) | Observational case-control | CCC | 22 | 25 | 32 ± 3 | 35 ± 11 | 80% | 76% | NR | NR | NR | CD57 | Blood | Secondary | Moderate | NR | There was a significant decrease in CD57 in lymphocytes of ME/CFS patients compared to HC. This decrease was significant in both percentage of cells expressing CD57 (p = 0.024) and the amount of CD57 molecule expressed per cell (p < 0.001) in T lymphocytes. In non- T lymphocytes only the amount of CD57 per cell was significantly different in ME/CFS patients compared to HC (p = 0.007). |
| Fletcher et al (2009) (36) | Observational case-control | Fukuda | 40 | 59 | 50 | 53 | 100% | 100% | NR | NR | NR | Cytokines | Blood | Tertiary | Complex | AUC IL-5 (0.84); AUC LTα (0.77); AUC IL-4 (0.77); IL-12 | The following cytokines were elevated in ME/CFS patients compared to HC: LTα, IL-1α, IL-1β,IL-4, IL-5, IL-6 and IL-12 and the following cytokines were decreased in ME/CFS patients: IL-8, IL-13 and IL-15 (p < 0.05). The following cytokines showed good biomarker potentials based on area under the curve: IL-5, LTα, IL-4 and IL-12 (p < 0.05). |
| Fletcher et al (2010A) (37) | Observational case-control | Fukuda | 176 | 230 | 44 (18-60) | 41 (23-74) | 83% | 86% | NR | NR | NR | NK cell cytotoxicity and dipeptidyl peptidase IV/CD26 | Blood | Tertiary | Moderate | AUC NK cell cytotoxicity: 0.776  AUC CD2+CD26+%: 0.746  AUC sCD26: 0.732  AUC rMolCD26/CD2+cell: 0.650 | NKCC was significantly lower in ME/CFS patients (p < 0.001). There was significantly higher percentage CD2^+^ lymphocytes positive for DPPIV/C26 in ME/CFS patients (p = 0.001) however levels of DPPIV/C26 expressed on T cells and NK cells were lower (p < 0.001). |
| Groven et al (2020) (45) | Observational case-control | Fukuda | 49 | 53 | 33.8 ± 11.3 | 39.4 ± 10.4 | 100% | 100% | 24.0 ± 3.6 | 24.7 ± 4.0 | NR | Immune markers  MCP-1 | Blood | Tertiary | Complex | NR | MCP-1 was significantly increased in ME/CFS patients compared to HC while other immune markers including IL-1β, Il-4, IL-6, TNF-α,TGF-β1, TGF-β2, TGF-β3, IL-10 and IL17 were significantly lower (p < 0.01) |
| Günther et al (2019) (46) | Observational case-control | CCC | Canada: 25  Norway: 25 | Canada: 25  USA: 6 | 30-62 | 30-62 | NR | NR | NR | NR | NR | Immunosignature | Blood | Tertiary | Moderate | AUC in discovery set: 0.75-0.73  AUC in Validation set: 0.60-0.82 | 256- peptide signatures were detected that could significantly differentiate between ME/CFS patients and HC based on area under the curve values (no p-value). |
| Halpin et al (2017) (48) | Observational case-control | CCC  Fukuda | 55 | 151 | 45.42 ± 8.4 | 46.9 ± 8.6 | 64% | 48% | NR | NR | NR | deoxyuridine triphosphate nucleotidohydrolases (enterovirus) | Blood | Primary | Moderate | NR | ME/CFS patients had higher levels of anti-EBV-dUTPase antibodies (p < 0.001) and anti- human dUTPase antibodies (p = 0.0241) compared to HC. |
| Hanevik et al (2012) (49) | Observational case-control | Fukuda | 19 | 10 | 45.5 ± 9.1 | 40.9 ± 11.7 | 79% | 72% | NR | NR | NR | NK-cell and CD8-cell populations | Blood | Secondary | Moderate | NR | In post-giardiasis ME/CFS patients had significantly lower NK- cell levels compared to HC. There was a negative correlation between abdominal and fatigue symptoms and NK- cell levels (p < 0.05). |
| Hardcastle et al (2015A) (50) | Observational case-control | Fukuda | Moderate: 15  Severe: 12 | 18 | Moderate: 45.93 ± 2.96  Severe: 41.25 ± 2.77 | 40.39 ± 2 | Moderate: 73.3%  Severe: 83.3% | 66.6% | NR | NR | 6.5 | Immune cell receptors | Blood | Secondary | Moderate | NR | Moderate ME/CFS patients had significantly increased CD8^+^CD45RA effector memory T cells, signalling lymphocytic activation molecule expression on NK cells, killer cell immunoglobulin-like receptor 2DL5A on CD4^+^ T cells and BTLA4^+^ on CD4^+^T central memory cells as well as significantly reduced CD8^+^T central memory LFA-1, total CD8^+^T KLRG1, naïve CD4^+^T KLRG1 and CD56^dim^CD16^−^NK cell CD2^+^ and CD18^+^CD2^+^ (p < 0.05). Severe ME/CFS patients had significantly increased CD18^+^CD11c^−^in the CD56^dim^CD16^−^NK cell phenotype and significantly reduced NKp46 in CD56^bright^CD16^dim^NK cells (p < 0.05). |
| Hardcastle et al (2015B) (115) | Prospective longitudinal linked panels | Fukuda | Moderate: 12  Severe: 12 | 18 | Moderate: 44.73 ± 12.90  Severe: 41.27 ± 10.05 | 41.94 ± 10.76 | Moderate: 67%  Severe: 83% | 72% | NR | NR | NR | Immune cell parameters | Blood | Secondary | Moderate | NR | iNKT CD62L increased in expression over time in moderate ME/CFS patients. CD56^bright^ NK receptors significantly differed in severe ME/CFS patients (p = 0.004). At six months, naïve CD8^+^T cells (p = 0.041), CD8^−^CD4^−^ (p = 0.024) and CD56^−^CD16^−^ iNKT phenotypes (p = 0.030), γδ2T cells (p = 0.035) and effector memory subsets were significantly increased in severe ME/CFS patients. Severe ME/CFS also had significantly reduced CD56^bright^CD16^dim^ NKG2D, CD56^dim^CD16^−^ KIR2DL2/DL3, CD94^−^CD11a^−^γδ1T cells and CD62L+CD11a−γδ1T cells at six months. |
| Hornig et al (2015) (51) | Observational case-control | Fukuda  CCC | Short-duration illness: 52  Long duration illness: 246 | 348 | Short duration illness: 40.5 ± 13.6  Long duration illness: 50.2 ± 11.4 | 48.5 (12.0) | Short-duration illness: 75%  Long duration illness: 73.6% | 74.7% | NR | NR | 13.2 ± 9.2 | Cytokine alterations | Blood | Tertiary | Complex | NR | There are distinct and significant alterations in plasma immune signatures in the early course of ME/CFS compared to those with long term changes. Early stages of ME/CFS had increased activation of pro- and anti-inflammatory cytokines (p < 0.05). |
| Jawad Kadhum et al (2018) (53) | Observational case-control | Fukuda | 53 | 35 | 40.96 ± 1.875 | 45.63 ± 2.071 | 64.2% | 65.7% | 25.957 ± 4.798 | 26.539 ± 2.025 | NR | Interleukin-17A | Blood | Secondary | Moderate | AUC: 0.972; Sensitivity: 98.1%; Specificity: 65.7%; Accuracy: 85.23% | IL-17A levels are significantly higher in ME/CFS patients compared to HC (p < 0.01). |
| Khaiboullina et al (2015) (54) | Observational case-control | CCC  Fukuda | 67 | 42 | NR | NR | 64% | 43% | NR | NR | NR | Cytokine | Blood | Tertiary | Complex | Sensitivity: 92.54%; Specificity: 33.33% | The following cytokines were significantly higher in ME/CFS patients compared to HC: TNF- α, IL-12, IL-4, IL-12, IL-1β, and IL-25. IL-17F and CXCL8 were significantly lower compared to HC (p < 0.05). |
| Landi et al (2016) (56) | Observational case-control | CCC  Fukuda | 100 | 79 | 50.5 ± 12.6 | 50.4 ± 12.6 | 72% | 78.5% | < 40 | < 40 | NR | IL-16, IL-7 and VEGF-A | Blood | Tertiary | Complex | LASSO - Sensitivity: 41% specificity: 94%  CART-1 – Sensitivity: 43%; specificity: 96%; CART-2 – Sensitivity 46%; Specificity: 96% | There was a significant reduction in the concentration of circulating interleukin (IL)-16 (p < 0.001), IL-7 (p < 0.001), Vascular Endothelial Growth Factor A (VEGF-A) (p = 0.01), CX3CL1 (p = 0.01) and monokine-induced-by-IFN-c(MIG; CXCL9) (p = 0.006) along with increases in the concentrations of eotaxin 2 (CCL24) (p = 0.007) in ME/CFS patients compared to HC. |
| Maes et al (2011) (62) | Observational case-control | Fukuda | 56 | 37 | 38.2 ± 14.0 | 42.5 ± 11.4 | 89.2% | 67.5% | NR | NR | NR | Plasma peroxides | Blood | Tertiary | Complex | NR | Plasma peroxide and serum oxLDL antibodies concentrations were significantly higher in ME/CFS patients compared to HC (p = 0.01). These parameters weakly correlated with the Fibromyalgia and Chronic Fatigue Syndrome (FF) rating scale (r = 0.27, p = 0.01). |
| Maes et al (2012) (64) | Observational case-control | Fukuda | 107 | 20 | 41.6 ± 13.3 | 43.4 ± 13.3 | 81.3% | 75% | ≤30 | - ≤ 30 | NR | Cell- mediated immunity, cytokines | Blood | Tertiary | Complex | IL-1: AUC = 0.832, Sensitivity = 62.2%, Specificity = 100%; TNFα: AUC = 0899, Sensitivity 74.3%, Specificity = 100%; neopterin: AUC = 0.865, Sensitivity = 71.6%, Specificity = 100%; lysozyme: AUC = 0.771, PMN-elastase: AUC = 0.923, Sensitivity = 78.0%, Specificity = 100% | Serum IL-1 (p < 0.001), TNFα (p < 0.001), neopterin (p < 0.001), lysozyme (p < 0.001) and plasma PMN-elastase (p = 0.007) are significantly higher in ME/CFS patients than in HC. |
| Maes et al (2015) (61) | Observational case-control | Fukuda | 139 | 40 | 39.9 ± 12.5 | 42.6 ± 10.6 | 82.7% | 90% | NR | NR | 5.0 ± 5.0 | Activation antigens on CD8+ T lymphocyte | Blood | Secondary | Moderate | NR | CD3^+^ (p = 0.005), CD8^+^ (p = 0.020), CD8^+^CD38^+^ (p = 0.007) and CD8^+^HLA-DR^+^ (p < 0.011) were significantly higher in ME/CFS compared to HC. |
| Maher et al (2005) (65) | Observational case-control | Fukuda | 30 | NR | 46 ± 10 | NR | 83.0% | 84.21% | NR | NR | 10 ± 7 | Perforin | Blood | Tertiary | Complex | NR | There was a significant reduction in NK (p = 0.0001) and T cell (p = 0.05) associated perforin. |
| Milivojevic et al (2020) (72) | Observational case- control | CCC  Fukuda | 39 | 41 | 52.06 ± 10.87 | 51.43 ± 11.89 | 76.9% | 78.0% | NR | NR | < 3 years (n=4)  >3 years (n=46) | Antigen driven clonal B cell expansion | Blood | Secondary | Moderate | AUC: 0.806-0.846 | There was a significant association with immunoglobulin heavy variable region 3-23/30 plasma proteome and ME/CFS (p < 0.001). ME/CFS patients with coexisting IBS showed a significant association with immunoglobulin lambda constant region 7 (p <0.001) and ME/CFS without IBS showed association with immunoglobulin kappa variable region 3–11 (p < 0.001). |
| Nijs et al (2010) (84) | Observational case-control | Fukuda | 22 | 22 | 34.3 ± 8.8 | 38.9 ± 15 | 100% | 100% | 24.1 ± 4.7 | 24.5 ± 4.8 | NR | Elastase, , IL- 1β, or complement split product levels | Blood | Tertiary | Moderate | NR | There was no difference in elastase activity, IL- 1β, or complement split product levels in ME/CFS patients or HC postexercise. |
| Rivas et al (2018) (91) | Observational case- control | CCC | 76 | 73 | 49.78 (20–80) | Mean: 48.71 (23–66) | 82.89% | 82.19% | NR | NR | 17.44 | T and NK cell phenotypes | Blood | Primary | Moderate | Accuracy: 70% | ME/CFS patients showed significantly lower values of T regulatory cells (CD4^+^CD25^++^(high)FOXP3+) and higher NKT-like cells (CD3^+^CD16^+/−^CD56^+^) compared to HC NKCD69 and NKCD56 were significantly higher and NKG2C were significantly lower in ME/CFS patients compared to HC (p < 0.05). |
| Singh et al (2016) (97) | Observational case- control | CCC  Fukuda | USA: 11  EURO: 10 | USA: 12  EURO: 10 | NR | NR | NR | NR | NR | NR | NR | Human self-antigens and endogenous retroviral sequences. | Blood | Primary | Secondary | Sensitivity = 92.9%; Specificity = 97.6% | Humoral immunity profiling identified 25 peptides that effectively differentiate between ME/CFS patients and HC (no p-value) |
| Sorensen et al (2003) (99) | Observational case- control | Fukuda | Allergic: 23  Nonallergic: 9 | 29 | 18-45 | 18-45 | NR | NR | NR | NR | NR | Complement activation | Blood | Tertiary | Complex | NR | Six hours post-exercise, the level of complement split product C4a was significantly increased (p < 0.01). |
| Sung et al (2020) (102) | Observational case- control | Fukuda | 11 | Family members: 22  Unrelated controls: 16 | 45.5 ± 21.3 | Family members: 46.2 ± 14.7  Unrelated controls: 42.8 ± 18.6 | 81.8% | Family members: 54.5 %  Unrelated controls: 75.0% | NR | NR | 22 | Antibody-dependent cell-mediated cytotoxicity | Blood | Secondary | Moderate | ME/CFS vs unrelated HC: 0.76  ME/CFS vs family without ME/CFS: 0.69 | ME/CFS had significantly lower antibody-dependent cell-mediated cytotoxicity (ADCC) compared to HC (AUC: 77%, p <0.02). Related family members also showed significantly lower ADCC. |
| Szklarski et al (2021) (104) | Observational case- control | CCC | 205 | 98 | 43 (32.5-50.5) | 29 (26-38) | 70.73% | 50% | NR | NR | NR | Autoantibodies | Blood | Secondary | Moderate | AUC: 0.754 | Lower concentrations of sCD26 were only found in female ME/CFS patients and diagnostic suitability could not be confirmed. There was, however, an association of low sCD26 with higher levels of autoantibodies against alpha1 adrenergic and M3 muscarinic acetylcholine receptors. In infection-triggered ME/CFS, sCD26 were positively associated with activated T cells (r: 0.153-0.316, p < 0.05), liver enzymes, creatin kinase (r: 0.247, p: <0.001), and lactate dehydrogenase (r: 0.173; p: 0.014) and negatively associated with interleukin-1 beta (r: -0.182; p: 0.018). |
| Theorell et al (2017) (107) | Observational case-control | CCC | Stockholm: 24  Oslo: 24 | Stockholm: 28  Oslo: 24 | Stockholm:  44 (34-63)*  Oslo  30 (18-52)* | Stockholm:  44 (24-65)*  Oslo  30 (20-46)* | Stockholm: 75%  Oslo: 79% | Stockholm: 71%  Oslo: 79% | NR | NR | Duration > 2 years %  Stockholm: 100%  Oslo: 88% | Cytotoxic lymphocyte phenotype and function | Blood | Secondary | Moderate | Sensitivity ≈ 70%; Specificity ≈ 70% | There were no significant changes in cytotoxic lymphocyte phenotype and function. |
| Tokunaga et al (2020) (109) | Observational case-control | ICC  Fukuda | 6 | 16 | 49.0±30.1 | 42.8±18.6 | 66.7% | 75.0% | NR | 22.5 | NR | Inflammatory markers | Blood | Tertiary | Moderate | NR | Non-classical monocytes were higher in patients compared to HC (p < 0.05) but there was no significant difference between ME/CFS patients and non-affected family members. |
| Vernon et al (2005) (110) | Observational case- control | Fukuda | Atlanta: 22  Wichita: 37 | Atlanta: 34  Wichita: 57 | Atlanta: 39  Wichita:46 | Atlanta: 38  Wichita: 42 | Atlanta: 86.36%  Wichita: 97.06% | Atlanta: 83.78%  Wichita: 57.89% | NR | NR | 5.75 | Autoantibodies | Blood | Secondary | Moderate | NR | Subsets of ME/CFS patients had greater levels of antibodies to microtubule- associated proteins 2 (MAP2) (p = 0.03) and single-stranded DNA (p = 0.04). Across the whole ME/CFS cohort there were no significantly elevated autoantibodies. |

| **Table S3. Metabolomics/ mitochondrial/ microbiome biomarkers** | | | | | | | | | | | | | | | | | |
| --- | --- | --- | --- | --- | --- | --- | --- | --- | --- | --- | --- | --- | --- | --- | --- | --- | --- |
| **Author** | **Study type** | **Criteria** | **Sample size** | | **Age (years, mean ± SD)** | | **Sex (female %)** | | **BMI (kg/m^2^)** | | **Illness duration (years)** | **Biomarker** | **Classification** | **Selectivity** | **Ease of detection** | **AUC; Sensitivity; Specificity** | **Findings** |
|  |  |  | **ME/CFS** | **HC** | **ME/CFS** | **HC** | **ME/CFS** | **HC** | **ME/CFS** | **HC** | **ME/CFS** |  |  |  |  |  |  |
| Armstrong et al (2012) (17) | Observational case-control | CCC | Males: 5  Females: 6 | Males: 5  Females: 5 | Males: 45.2±12.4  Females: 49.7±6.6 | Males: 47.8±15.4  Females: 50.2±7.4 | 54.55% | 50% | NR | NR | NR | Metabolites | Blood | Secondary | Moderate | NR | There was significantly lower glutamine (p = 0.002) and ornithine (p < 0.05) in blood from ME/CFS patients compared to HC. Correlation analysis showed associations with glucogenic amino acids and metabolites involved in the urea cycle. |
| Ciregia et al (2016) (114) | Observational case/ control/ twin study | Fukuda | 45 | 45 | 38.6 ± 11.9 | 36.4 ± 10.9 | 51% | 56% | NR | NR | 7.2 ± 5.6 | Mitochondrial proteins | Blood | Secondary | Moderate | NR | The following mitochondrial proteins: aconitate hydratase (p < 0.05), ATP synthase subunit beta (p < 0.01) were upregulated in ME/CFS patients compared to twin control. |
| Domingo et al (2021) (28) | Observational case- control | Fukuda | 21 | 20 | 47.3 ± 1.5 | 54.6 ± 4.3 | 100% | 100% | 23.6 ± 0.5 | 25.0 ± 2.7 | NR | Circulating FGF21 and NT-proBNP levels | Blood | Tertiary | Complex | NR | ME/CFS patients had significantly decreased plasma levels of antioxidant capacity (p = 0.009) and higher lipoperoxide (p = 0.021), inflammatory cytokines, FGF21 and NT-proBNP (p < 0.05). There were significant positive correlations between NT-proBNP and IL-1b (p= 0.04) and IL6 (p = 0.01) compared to HC. |
| Esfandyarpour et al (2019) (32) | Observational case-control | CCC | 20 | 20 | NR | NR | NR | NR | NR | NR | NR | Whole blood serum and plasma | Blood | Tertiary | Moderate | NR | ME/CFS patients’ response to impedance pattern was significantly different compared to HC (p < 0.001). |
| Fukuda et al (2016) (39) | Observational case-control | Fukuda | 121 | HV1: 12  HV2: 24  HV3: 656 | 37.3 ± 8.36 | HV1: 20.4 ± 0.5  HV2: 36.7 ± 8.8  HV3: 40.8 ± 12.4 | 63% | HV1: 100%  HV2: 50%  HV3: 58% | ≤ 28 | ≤ 28 | NR | Oxidative stress index  (d-ROMS) | Blood | Tertiary | Complex | NR | In the resting state, ME/CFS patients had significantly higher oxidative stress index and lower anti-oxidative activity (p < 0.001). |
| Germain et al (2017) (41) | Observational case-control | Fukuda  IOMC | 17 | 15 | 53.9 ± 8.6 | 51.9 ± 6.2 | 100% | 100% | 25.2 ± 5.5 | 26.6 ± 5.6 | NR | Fatty acid and lipid metabolism | Blood | Tertiary | Complex | NR | Thirty-five metabolites were found to be significantly differentially accumulating in ME/CFS patients compared to HC following statistical correction (q < 0.15). |
| Germain et al (2018) (42) | Observational case- control | Fukuda | 32 | 19 | 48.5 ± 13.7 | 49.2 ± 10.5 | 100% | 100% | 25.5 ± 5.7 | 26.8 ± 5.9 | <3 n=6; >3 n=26 | Plasma metabolomics | Blood | Tertiary | Complex | AUC Pyroglutamine and heme: 0.75 | Levels of 14 metabolites significantly differed in ME/CFS patients compared to HC (p < 0.05) |
| Kitami et al (2020) (55) | Observational case-control | Fukuda  ICC  IOMC | 48 | 52 | NR | NR | NR | NR | <30 | <30 | NR | Various | Blood | Tertiary | Complex | NR | Twenty-six potential markers were identified through deep phenotyping especially monocyte number, microbiome abundance, and lipoprotein profiles (p < 0.05). Lipoprotein and microbiome profiles correlated with sleep disruption. All these biomarkers occur early in the course of the illness. |
| Mandarano et al (2018) (66) | Observational case- control | Fukuda | Taxa abundance comparisons: 17  Diversity subgroup: 11 | Taxa abundance comparisons: 17  Diversity subgroup: 10 | Taxa abundance comparisons: 52 ± 11.9  Diversity subgroup: 54 ± 11.6 | Taxa abundance comparisons: 44.6 ± 10.9  Diversity subgroup: 43.6 ± 13.6 | Taxa abundance comparisons: 76.5%  Diversity subgroup: 63.0% | Taxa abundance comparisons: 94.1%  Diversity subgroup: 90.0% | Taxa abundance comparisons: 26.8 ± 4.7  Diversity subgroup: 26.2 ± 4.9 | Taxa abundance comparisons: 27.4 ± 4.5  Diversity subgroup: 27.7 ± 5.2 | NR | Eukaryotes in gut microbiota | Blood | Tertiary | Moderate | NR | There was a no significant changes in eukaryotic diversity in ME/CFS patients compared to HC. |
| Missailidis et al (2020) (73) | Observational case- control | CCC | 51 | 22 | 51 (26–71) | 43 (21–67) | 86% | 68% | NR | NR | NR | Mitochondrial markers | Blood | Tertiary | Complex | Combination of lymphocyte 48 h death rate, lymphoblast respiratory dysfunction and lymphoblast TORC1 activity: AUC = 0.61, Sensitivity = 97%, Specificity = 100% | Lymphocyte death rate, mitochondrial respiratory function and TORC1 activity showed greater than 90% sensitivity for diagnostic differentiation of ME/CFS patients compared to HC (p < 0.001). |
| Nagy-Szakal et al (2017) (77) | Observational case-control | CCC  Fukuda | 50 | 50 | 51.081 ± 1.607 | 51.320 ±1.620 | 82% | 82% | High BMI (>25 kg/m^2^): 56%  Normal BMI (<25 kg/m^2^): 44% | High BMI (>25 kg/m^2^): 44%  Normal BMI (<25 kg/m^2^): 56% | < 3 years (n=4)  >3 years (n=46) | Fecal metagenomic profiles | Stool | Tertiary | Complex | AUC = 0.831; Cross-validated AUC = 0.684 | ME/CFS patients with IBS had more abundant unclassified Alistipes and less Faecalibacterium compared to HC (p < 0.05). ME/CFS patients without IBS had greater unclassified B*acteroides* and less *Bacteroides Vulgatus* (p < 0.05). ME/CFS patients had lower metabolic pathways associated with unsaturated fatty acid biosynthesis and increased atrazine degradation pathways independent of IBS comorbidity (p < 0.05). |
| Nagy-Szakal et al (2018) (76) | Observational case- control | CCC  Fukuda | 50 | 50 | 51.081 ± 1.607 | 51.320 ±1.620 | 82% | 82% | High BMI (>25 kg/m^2^): 56%  Normal BMI (<25 kg/m^2^): 44% | High BMI (>25 kg/m^2^): 44%  Normal BMI (<25 kg/m^2^): 56% | < 3 years (n=4)  >3 years (n=46) | Metabolomics/ metagenomics | Stool and  Blood | Tertiary | Complex | Metabolomic: Cross-validated AUC = 0.820  Metagenomic AUC = 0.745  Metabolomic and metagenomic: Cross-validated AUC=0.836 | ME/CFS patients have significantly lower levels of phosphatidylcholine (p = 0.017), choline and carnitine (p = 0.017) compared to HC. ME/CFS patients with IBS had significantly higher levels of triglyceride (p = 0.004) and ceramide (p = 0.021). |
| Shukla et al (2015) (95) | Observational case- control | Fukuda | 10 | 10 | 48.6 ± 10.5 | 46.5 ± 13.0 | 80.0% | 80.0% | 23.9 ± 4.3 | 24.6 ± 3.3 | NR | Microbiome | Stool  Blood- | Tertiary | Complex | NR | Several microbiome phyla microbiome compositions significantly differed between ME/CFS patients and HC. Following 72 hours post exercise, there was an increase in relative abundance of 6 of the 9 major bacteria phyla (MBP) compared to only 2 of the 9 MBP (p = 0.005). Clearance of certain groups of bacterial phyla in blood was also significantly higher in ME/CFS compared to HC post- exercise. |
| Simonato et al (2021) (96) | Observational case-control |  | 40 | 40 | 32 (23–46) | 33 (22-46) | 65% | 65% | NR | NR | 6 (3–12) | Tryptophan metabolites, cytokines, and fatty acid binding protein 2 | Blood | Tertiary | Complex | NR | IL-17A (p = 0.018), FABP-2 (p = 0.002) and 3-hydroxykynurenine (p = 0.037) were increased in ME/CFS patients compared to HC while kynurenine (p = 0.012) and serotonin (p = 0.045) were lower in ME/CFS patients. Changes in kynurenine and 3-hydroxykynurenine were associated with higher kynurenic acid/kynurenine and 3-hydroxykynurenine/kynurenine ratios. |
| Sweetman et al (2020) (103) | Observational case- control | CCC | 11 | 9 | 43.2 (11.3-69)* | 38.0 (12.5-60)* | 63.63% | 66.6% | 23.7* | NR | 11* | SWATH-MS analysis | Blood | Tertiary | Complex | NR | ME/CFS patients had 60 differentially expressed proteins following SWATH- MS analysis (p < 0.01). |

| **Table S4. Endovascular/ circulatory biomarkers** | | | | | | | | | | | | | | | | | |
| --- | --- | --- | --- | --- | --- | --- | --- | --- | --- | --- | --- | --- | --- | --- | --- | --- | --- |
| **Author (date)** | **Study type** | **Criteria** | **Sample size** | | **Age (years, mean ± SD)** | | **Sex (female %)** | | **BMI (kg/m^2^)** | | **Illness duration (years)** | **Biomarker** | **Classification** | **Selectivity** | **Ease of detection** | **AUC; Sensitivity; Specificity** | **Findings** |
|  |  |  | **ME/CFS** | **HC** | **ME/CFS** | **HC** | **ME/CFS** | **HC** | **ME/CFS** | **HC** | **ME/CFS** |  |  |  |  |  |  |
| Almenar-Pérez et al (2020) (16) | Observational case-control | CCC  Fukuda | 15 | 15 | 46.8* ( 38–53) | 45.2* ( 18–52) | 100% | 100% | <40 | <40 | 18.4* (1.5-30.9) | microRNAs from peripheral mononuclear cells and extracellular vesicles. | Blood | Secondary | Moderate | AUC: 15 significant mRNA had AUC > 0.75; Sensitivity: 96%; specificity: <50% | ME/CFS patients had significantly higher extracellular vesicle (EV) count and EVs were also significantly smaller compared to HC (p < 0.05). Blood creatine kinase (CK) was significantly lower compared to HC (p < 0.05). Zeta-potential was significantly different in ME/CFS patients compared to HC, where ME/CFS patients presented with more negative values regardless of whether the EV were isolated in the presence or absence of proteinase K (p < 0.05). CK blood values, plasma EV physical characteristics (including counts, size, and zeta- potential) miRNAs were significantly associated with severe ME/CFS (p < 0.05). |
| Bonilla et al (2022) (19) | Observational case- control | Fukuda | Severe: 10  Mild: 10 | 20 | Severe: 54.3  Mild: 50.1 | 51.2 | Severe: 90%  Mild: 80% | 80% | NR | NR | NR | Extracellular vesicles | Blood | Secondary | Moderate | NR | There was no significant association between severe ME/CFS and levels of extracellular vesicles carrying the B cell marker CD19 and platelet marker CD41a following adjustments for multiple comparisons. |
| Castro-Marrero et al (2018) (24) | Observational case-control | Fukuda | 10 | 5 | 46.3 ± 8.5 | 44.2 ± 6.2 | 100% | 100% | NR | NsR | NR | Circulating extracellular vesicles | Blood | Secondary | Moderate | NR | Compared to HC, extracellular Vesicles (EV)- enriched fraction was significantly higher in ME/CFS patients (p = 0.007) and EVs were significantly smaller (p = 0.014). |
| Eguchi et al (2020) (30) | Observational case-control | CCC  Fukuda | ME/CFS 1: 39  ME/CFS 2: 30 | 33 | ME/CFS 1: 40.08±0.96  ME/CFS 2: 39.77±1.11 | 40.18±0.98 | ME/CFS 1: 71.79%  ME/CFS 2: 100% | 75.76% | ME/CFS 1: 22.34±0.70  ME/CFS 2: 20.24±0.59 | 22.07±0.69 | NR | Extracellular vesicles | Blood | Tertiary | Complex | AUC Circulating EV = 0.802 | Circulating EV number was significantly higher in ME/CFS patients compared to HC (p < 0.001). Circulating EV number correlated with serum c-reactive protein levels (p = 0.442, p = 0.0007). Variations in Talin-1, Filamin-A and 14-4-4 family proteins were identified by proteomic analysis in ME/CFS patients (p < 0.05) |
| Fenouillet et al (2016) (34) | Observational case-control | IOMC | 36 | 11 | 42 ± 7 | 46 ± 5 | 67% | 54% | NR | NR | 5±1 | post-exercise M-wave, TBARS variations and CD26-expression at rest | Physical | Tertiary | Moderate | NR | There were significant differences in three biological variables post-exercise M-wave, thiobarbituric acid reactive substances: (TBARS) variations and CD26- expression at rest. These were correlated with each other. Health-related quality of life was negatively correlated with exercise-induced TBARS increase (r: 0.570, p < 0.001) and positively correlated with CD26-expression (r: 0.486, p < 0.01). The pain component of SF-36 was negatively correlated with CD26-expression (r: 0.618, p < 0.001). The TBARS increase and the M-wave decrease were the highest, and the CD26-expression level were the lowest in those who reported an infectious onset. |
| Fletcher et al (2010B) (35) | Observational case-control | Fukuda | 93 | 100 | 44 ± 9 | 41 ± 10 | 83% | 84% | NR | NR | NR | Plasma neuropeptide Y | Blood | Tertiary | Moderate | AUC: 0.655 | Plasma neuropeptide Y (NPY) was significantly higher in ME/CFS patients compared to HC (p < 0.001). NPY had significant associations with various subjective measures including perceived stress and depression levels (p < 0.05). |
| Gravelsina et al (2021) (44) | Observational case- control | Fukuda | Males: 42  Females: 92 | Males: 41  Females: 13 | Males: 23-76  Females: 23-68 | Males: 18-65  Females: 18-61 | 68.66% | 24.07% | NR | NR | NR | Activin B | Blood | Tertiary | Complex | NR | ME/CFS patients and HC did not have significantly different activin B levels. |
| Haffke et al (2022) (47) | Observational case-control | CCC | PAT study group: 14  Validation study group: 26 | PAT study group: 15  Validation study group: 50 | PAT study group: 44.5 (24–59)  Validation study group: 46.5 (21–62) | PAT study group: 43 (23–58)  Validation study group: 38 (19–65) | PAT study group: 85.7%  Validation study group: 88.5% | PAT study group: 86.6%  Validation study group: 70% | PAT study group: 23.78 (20.24–31.83)  Validation study group: NR | PAT study group: NR  Validation study group: NR | NR | Endothelial dysfunction | Endothelial | Tertiary | Complex | NR | Out of 14 post-COVID ME/CFS patients, five showed diminished reactive hyperaemia index (RHI) (p ≤0.05). HC did not show significantly lower RHI. |
| Lidbury et al (2017) (57) | Observational case-control | CCC | 45 | 17 | 19–66 | 24–60 | 89% | 76.0% | 22.2 (18.9–47.2) | 21.9 (19.6–32.1) | NR | Activin B | Blood | Tertiary | Complex | AUC: 0.724; | Serum activin B levels were significantly higher compared to HC (p = 0.002) |
| Lidbury et al (2019) (58) | Observational case-control | **CCC**  **ICC** | 45 | 17 | 19–66 | 24–60 | 89% | 76.0% | 22.2 (18.9–47.2) | 21.9 (19.6–32.1) | NR | Activin B | Blood and urine | Tertiary | Complex | AUC: 0.755 | Machine learning indicated that Serum activin B (p <0.001) and associated markers 24-h urinary creatinine clearance ( p = 0.02) and serum urea (p = 0.002) were significantly higher in ME/CFS patients compared to HC. |
| Maes et al (2005) (63) | Observational case-control | Fukuda | 20 | 12 | NR | NR | NR | NR | NR | NR | NR | DHEAS, IGF1 | Blood | Tertiary | Complex | AUC: 0.804; Sensitivity: 70.4%; Specificity: 90.9% | There was significantly lower serum dehydroepiandrosterone-sulfate (DHEAS) in ME/CFS patients compared to HC (p = 0.003). There were no significant differences in IGF1 or IGFBP3/IGF1 ratio. |
| Melvin et al (2019) (70) | Observational case- control | CCC  Fukuda | Non-severe cases: 24  Severe cases: 16 | 40 | Non-severe cases: 47 (42–51.9)  Severe cases: 39.8 (33–46.5) | 42.9 ± 39.1–46.6) | Non-severe cases: 66.7%  Severe cases: 68.8% | 57.5% | Non-severe cases: 27.1 (24.9–29.3)  Severe cases: 22.9 (20.1–25.7) | 42.9 (39.1–46.6) | NR | GDF15 | Blood | Tertiary | Moderate | NR | GDF15 levels in severe ME/CFS patients was significantly elevated (p = 0.01) and this positively correlated with fatigue scores (p = 0.026). Circulating levels of GDF15 were stable at two different time points (across seven months) in mild/ moderate patients. |
| Nacul et al (2019) (74) | Observational case- control | CCC  Fukuda | Mild/moderate: 216  Severe: 56 | 136 | 18-60 | 18-60 | Mild/ moderate: 76.9%  Severe: 76.8% | 61.8% | Mild/moderate: 27.17  Severe: 23.73 | 25.96 | NR | Serum creatine kinase | Blood | Secondary | Moderate | AUC = 0.67; Sensitivity = 96%; Specificity = <50% | Severe ME/CFS patients had significantly lower median values of serum creatine kinase compared to HC and non-severe ME/CFS patients (p < 0.001). |
| Nkiliza et al (2021) (85) | Observational case-control | CCC  Fukuda | 50 | 50 | Females: 48.4 ± 9.9  Males: 38.7 ±12.9 | Females: 44.7 ± 12.2  Males: 34.5 ± 9.7 | 50% | 50% | Females: 26.9 ± 4.9  Males: 26.2 ± 4.7 | Females: 27.2 ± 2.8  Males: 27.4 ± 3.6 | NR | Plasma lipid profiles | Blood | Secondary | Moderate | NR | Male ME/CFS patients had significantly higher hexosylceramides (HexCer), monounsaturated plasma phospholipids (PL), saturated triglycerides compared to HC (p < 0.05). In Female ME/CFS patients, levels of total phosphatidylethanolamine (PE), omega-6 arachidonic acid-containing PE, and total HexCer (p , 0.05). Omega-6 linoleic acid-derived oxylipins were significantly higher in male ME/CFS patients compared to male HC (p < 0.05). Principal component analysis showed that most of PC and some PE, PI and SM species were negatively associated with headache and fatigue severity this correlation was not sex-specific (p < 0.05). Lower correlations of oxylipins and ethanolamides were associated with headaches, fatigue, and cognitive difficulties. This correlation was dependent on sex (p < 0.05). |
| Shishioh-Ikejima et al (2010) (94) | Observational case- control | Fukuda | 6-60 months  25  61-120 months  30 | 30 | 6-60 months  33.6 ± 1.7  61-120 months  36.9 ± 1.5 | 36.1 ± 1.6 | 63.63% | 63.3% | 6-60 months  21.4 ± 0.63  61-120 months  21.1 ± 0.59 | NR | 0.5-5 (n=25)  5-12 (n=30) | alpha-melanocyte-stimulating hormone | Blood | Tertiary | Moderate | NR | Mean alpha-melanocyte-stimulating hormone concentration was significantly higher in ME/CFS patients compared to HC (p = 0.02). There was a negative correlation between duration of illness and plasma alpha-melanocyte-stimulating hormone concentration (p = 0.04). In patients that were diagnosed ≤5 years there was a significant difference in alpha-melanocyte-stimulating hormone concentration compared to HC (p < 0.01) |
| Sørland et al (2021) (100) | Observational case-control | CCC | 40 | NR | 42.4 (21.5-61.1) | NR | 77.5% | NR | 23.3 (17.1-33.1) | NR | 2-5: 17.5%, 5-10: 32.5% 10-15: 22.5% >15: 27.5% | Endothelial function | Physical | Secondary | Complex | NR | Endothelial dysfunction, as measured by flow- mediated dilation and post- occlusive reactive hyperemia was significantly lower in ME/CFS patients compared to HC (p = 0.005 and p = 0.003, respectively). |
| Stringer et al (2013) (101) | Observational case- control | Fukuda | 10 | 10 | 52.9 | 53.0 | 100% | 100% | NR | NR | 15 | Leptin-driven cytokine fluctuations | Blood | Tertiary | Complex | NR | Fatigue severity significantly correlated (r= 0.303, p< 0.01) with leptin. |
| Thambirajah et al (2008) (105) | Observational case- control | Fukuda | 6 | 7 | 51.5 ± 8.46 | 52.3 ± 6.40 | 71.43% | 83.3% | NR | NR | NR | Heat- shock protein expression | Blood | Secondary | Moderate | NR | Basal HSP27 was significantly higher in ME/CFS patients compared to HC (p < 0.01). Levels of HSP27, HSP60 and HSP90 significantly decreased post-exercise in ME/CFS patients (p < 0.05) while remaining relatively constant in HC. This decrease of HSP in ME/CFS patients following exercise was corroborated using a repeated measures one-way ANOVA (p < 0.05). |

|  | | | | | | | | | | | | | | | | | |
| --- | --- | --- | --- | --- | --- | --- | --- | --- | --- | --- | --- | --- | --- | --- | --- | --- | --- |
| **Table S5. Neurological biomarkers** | | | | | | | | | | | | | | | | | |
| **Author** | **Study type** | **Criteria** | **Sample size** | | **Age (years, mean ± SD)** | | **Sex (female %)** | | **BMI (kg/m^2^)** | | **Illness duration (years)** | **Biomarker** | **Classification** | **Selectivity** | **Ease of detection** | **AUC; Sensitivity; Specificity** | **Findings** |
|  |  |  | **ME/CFS** | **HC** | **ME/CFS** | **HC** | **ME/CFS** | **HC** | **ME/CFS** | **HC** | **ME/CFS** |  |  |  |  |  |  |
| Mathew et al (2009) (69) | Observational case- control | Fukuda | 16 | 15 | 37.6 ± 9.9 | 35.3 ± 10.3 | 69% | 60% | 24.3 ± 5.3 | 25.9 ± 4.3 | 5.7 ± 5.0 | Ventricular cerebrospinal fluid lactate | Neuroimaging | Secondary | Complex | NR | ME/CFS patients ventricular lactate concentrations were elevated by 348% compared to HC (p < 0.001). |
| Natelson et al (2017) (79) | Observational case-control | Fukuda | 38 | 29 | NR | NR | NR | NR | NR | NR | NR | Ventricular lactate | Neuroimaging | Tertiary | Moderate | NR | ME/CFS, fibromyalgia and ME/CFS and fibromyalgia did not significantly differ in CSF lactate levels but all groups were significantly higher than HC (p < 0.016). |
| Okada et al (2004) (86) | Observational case- control | Fukuda | 16 | 49 | NR | NR | NR | NR | NR | NR | 5.82 | acetyl-L-carnitine uptake in prefrontal cortex. | Neuroimaging | Secondary | Complex | NR | ME/CFS patients had significantly reduced gray- matter volume in the bilateral prefrontal cortex. The volume reduction in the prefrontal cortex was related to the severity of fatigue (p = 0.004). |
| Provenzano et al (2020) (89) | Observational case- control | Fukuda | 38 | 31 | 47.74 ± 16.46 | 43.9 ± 16.3 | 89.4% | 74.2% | 26.20+4.52 | 28.4 ± 4.5 | NR | fMRI patterns | Neuroimaging | Tertiary | Complex | Pre-exercise (day 1): Sensitivity = 87.5%; Specificity = 76.9%; Accuracy = 80.9%; Post-exercise (day 2): Sensitivity = 76.9%; Specificity = 75%; Accuracy = 76.1%. | Logistic regression model on fMRI data was able to significantly differentiate between ME/CFS and HC (p < 0.05). |
| Rayhan et al (2021) (90) | Observational case-control | CCC  Fukuda | 34 | 24 | 46.9±12.8 | 41.4±17.9 | 73.53% | 41.7% | NR | NR | NR | Submaximal exercise  MRI | Neuroimaging | Tertiary | Complex | NR | There was an increase in exercise- induced spontaneous activity in the medial prefrontal cortex (anterior node of the default mode network) compared to HC (p < 0.001). ME/CFS patients had lower BOLD signals compared to HC (p < 0.05). |
| Shan et al (2018) (93) | Observational case- control | Fukuda | 45 | 27 | 47.12 ± 11.67 | 43.10 ± 13.77 | 73.0% | 60.0% | 26.58 ± 5.53 | 25.28 ± 4.18 | NR | Default Mode Network | Neuroimaging | Secondary | Complex | NR | The default mode network regions were more complex in ME/CFS during the task (p < 0.05). The posterior cingulate cortex which is a prominent driving region of the default mode network had more complex BOLD signals in both the resting state and during a task (p < 0.05). Functional connectivity was weaker between the medial prefrontal cortex and both inferior parietal lobules (p < 0.05). |
| Thapaliya et al (2021) (106) | Observational case- control | ICC  Fukuda | ME/CFS Fukuda: 25  ME/CFS ICC: 18 | 26 | ME/CFS Fukuda: 49.8 ± 12.22  ME/CFS ICC:43.26 ± 10.72 | 43.10 ± 13.7 | ME/CFS Fukuda: 80.0%  ME/CFS ICC:66.6% | 65.38% | < 35 | <35 | NR | neuronal microstructural changes | Neuroimaging | Secondary | Moderate | NR | There were no significant differences in axial diffusivity and mean diffusivity between Fukuda ME/CFS patients and HC. ICC ME/CFS patients had decreased axial diffusivity (p = 0.001) and mean diffusivity (p = 0.01) in the descending cortico-cerebellar tract in the midbrain and pons compared to HC. There was increased transverse diffusivity in the medulla and anisotropy was significantly decreased (p = 0.002) in a cluster region in the superior longitudinal fasciculus region of ICC ME/CFS patients. |
| Zeineh et al (2015) (112) | Observational case-control | Fukuda | 15 | 14 | 46.5 ± 13.2 | 46.6 ± 14.6 | 53.3% | 57.1% | NR | NR | 12.1 ± 6.9 | Right arcuate fasciculus abnormality MRI | Neuroimaging | Secondary | Moderate | AUC = 0.918; Sensitivity = 81.8%; Specificity = 100% | ME/CFS patients had higher FA in the right arcuate fasciculus (p=0.0015); this increase correlated with disease severity (r = 0.649, p = 0.026). ME/CFS patients who were right-handed FA was higher in the right inferior longitudinal fasciculus (p = 0.0008). Bilateral white matter volumes were significantly lower in ME/CFS patients compared to HC (p = 0.0026). |

| **Table S6. Ion channel biomarkers** | | | | | | | | | | | | | | | | | |
| --- | --- | --- | --- | --- | --- | --- | --- | --- | --- | --- | --- | --- | --- | --- | --- | --- | --- |
| **Author (date)** | **Study type** | **Criteria** | **Sample size** | | **Age (years, mean ± SD)** | | **Sex (female %)** | | **BMI (kg/m^2^)** | | **Illness duration (years)** | **Biomarker** | **Classification** | **Selectivity** | **Ease of detection** | **AUC; Sensitivity; Specificity** | **Findings** |
|  |  |  | **ME/CFS** | **HC** | **ME/CFS** | **HC** | **ME/CFS** | **HC** | **ME/CFS** | **HC** | **ME/CFS** |  |  |  |  |  |  |
| Cabanas et al (2018) (23) | Observational case-control | CCC | 12 | 12 | 42.5 ± 6.05 | 42.8 ± 5.47 | 80% | 80% | 23.54 ± 0.54 | 23.35 ± 1.37 | NR | TRPM3 | Blood | Secondary | Moderate | NR | The TRPM3 current amplitude following pregnenolone sulfate stimulation in NK cells from ME/CFS patients was significantly reduced (p < 0.001) compared to HC. This current was significantly influenced by ononetin (p < 0.001). |
| Eaton- Fitch et al (2022) (29) | Observational case-control | CCC  ICC | 10 | 10 | 43.9 ± 10.71 | 44.10 ± 10.39 | 90% | 90% | 25.74±5.49 | 23.68±3.96 | 17.70±14.95 | TRPM3 | Blood | Secondary | Moderate | NR | At baseline, calcium influx as determined by amplitude and half-time of calcium response in NK cells from ME/CFS patients is significantly lower compared to HC (p < 0.001). |
| Nguyen et al (2016) (83) | Observational case-control | Fukuda | 15 | 25 | 48.82 ± 9.83 | 39.2 ± 12.12 | 70.6% | 68% | 25.602 ± 4.945 | 25.387 ± 4.383 | NR | TRPM3 | Blood | Secondary | Moderate | NR | Unstimulated CD56^bright^CD16^dim/–^ NK cells from ME/CFS patients showed significantly lower TRPM3 receptors compared to HC (p < 0.05). There was no significant difference in Ca2+ influx. Pregnenolone Sulfate (PregS)- stimulated CD56^bright^CD16^dim/–^ NK cells had significantly higher Ca2+ influx compared to HC (p < 0.05). CD56^dim^ CD16^+^ NK cells had no significant difference in TRPM3 expression and Ca2+ flux. TRPM3 expression was significantly higher in CD56^dim^ CD16^+^ NK cells but ca2+ was not significantly affected. Thapsigargin significantly increased cell lysis of target cell line K562 prior to PregS stimulation in ME/CFS patients compared to HC. |

| **Table S7. Physical biomarkers** | | | | | | | | | | | | | | | | | |
| --- | --- | --- | --- | --- | --- | --- | --- | --- | --- | --- | --- | --- | --- | --- | --- | --- | --- |
| **Author (date)** | **Study type** | **Criteria** | **Sample size** | | **Age (years, mean ± SD)** | | **Sex (female %)** | | **BMI (kg/m^2^)** | | **Illness duration (years)** | **Biomarker** | **Classification** | **Selectivity** | **Ease of detection** | **AUC; Sensitivity; Specificity** | **Findings** |
|  |  |  | **ME/CFS** | **HC** | **ME/CFS** | **HC** | **ME/CFS** | **HC** | **ME/CFS** | **HC** | **ME/CFS** |  |  |  |  |  |  |
| Allen et al (2012) (15) | Observational case-control | Fukuda | 14 | 14 | 50 ± 14 | 42 ± 14 | NR | NR | 25.0 ± 3.0 | 26.0 ± 4.9 | NR | Pulse wave abnormalities | Physical | Tertiary | Moderate | AUC = 0.81; Sensitivity = 86%; specificity = 64%;  Accuracy = 82% | Overall pulse- timing response to controlled standing across all sites were significantly reduced in ME/CFS patients (p = 0.002). There was no significant difference in pulse amplitude between both groups. Changes in pulse amplitude in combination with tilt was found to be weakly significantly and negatively correlated with fatigue severity (p < 0.05). |
| Escorihuela et al (2020) (31) | Observational case-control | Fukuda | 45 | 25 | 46.41 ± 0.84 | 44.96 ± 1.30 | 100% | 100% | 24.59 ± 0.69 | 23.77 ± 0.61 | NR | Heart rate variability | Physical | Tertiary | Moderate | NR | ME/CFS patients had decreased intervals between consecutive heartbeats (RR) (p < 0.01) and heart rate variability (HRV) time and frequency domain parameters (p < 0.005). There was a significant correlation between questionnaire scores and HRV time and frequency-domain parameters (p < 0.05). There was also a significant correlation between RR scores and self-reported fatigue (p = 0.005). |
| Frith et al (2012) (38) | Observational case-control | Fukuda | 68 | 68 | 46.6 ± 12.1 | 47.9 ± 13.4 | 62% | 62% | NR | NR | NR | Heart rate and non-invasive continuous blood pressure measurements | Physical | Tertiary | Easy | Sensitivity: 77%; Specificity: 53% | Low frequency-HRV was significantly higher in ME/CFS patients compared to HC at rest while parasympathetics markers were significantly lower (p = 0.006). Across all domains total diastolic blood pressure variability was increased (p = 0.0003). |
| Gao et al (2013) (40) | Observational case-control | Reeves/ Fukuda | 23 | 41 | 47.6 | 46.3 | 91.3% | 75.6% | 28.3 | 26.8 | NR | Heart-rate variability | Physical | Tertiary | Complex | AUC: 0.65 | There are significant differences in heart-rate variability at baseline prior to social stress test (p < 0.001). During the stress-test there is no significant differences in heart-rate variability. |
| Nacul et al (2018) (75) | Observational case-control | CCC  Fukuda | Moderate: 216  Severe: 56 | 136 | Moderate: 47.1 ± 11.0  Severe: 45.9 ± 11.5 | 45.4 ± 12.0 | Moderate: 76.9%  Severe: 76.8% | 62.8% | Moderate: 6.4 (6.0)  Severe: 24.3 (5.0) | 24.9 (4.3) | NR | Hand grip strength | Physical | Tertiary | Moderate | NR | Mild/ moderate ME/CFS was associated with a reduction in minimum hand grip strength (HGS) of 10.5kg (p < 0.001). Severe ME/CFS was associated with a reduction in HGS of 15.3kg (p < 0.001). These HGS indicators had significant correlation with clinical parameters: disease severity, fatigue analog scale, pain analog scale, and physical component summary (p < 0.001) |
| Natelson et al (2007) (78) | Observational case-control | Fukuda | 75 | 40 | 43.3 ± 10.5 | 40.4 ±7.9 | 80% | 90% | NR | NR | NR | Hypocapnia | Physical | Tertiary | Moderate | NR | Orthostatic hypocapnia rates were significantly higher in ME/CFS (20.6%) than HC (2.9%; p < 0.02). |
| Nelson et al (2019) (81) | Observational case-control | ICC  CCC  Fukuda | 16 | 10 | 50.3 ± 12.5 | 49.8 ± 13.7 | 56.25% | 50% | 25.9 ± 5.3 | 24.6 ± 3.0 | 8.1±4.7 | 2- day cardiopulmonary exercise testing | Physical | Tertiary | Complex | Decrease in Work Rate at Ventilatory Threshold of 6.3-9.8%  VT -6.3%: Sensitivity = 87.5%; Specificity = 90%  VT -9.8%: Sensitivity = 68.8%; Specificity = 100% | Work rate (WR) at ventilatory threshold decreases by 6.3-9.8% in ME/CFS patients compared to HC on day two of CPET (p < 0.05). |
| Nelson et al (2021) (80) | Observational case-control | ICC  CCC  Fukuda | 16 | 10 | 50.3 ± 12.5 | 49.8 ± 13.7 | 56.3% | 50% | 25.9± 5.3 | 24.6± 3.0 | 8.1±4.7 | 2- day cardiopulmonary exercise testing | Physical | Tertiary | Complex | AUC = 0.748; Sensitivity = 63%; Specificity = 40% | There was significantly lower post-exercise heart rate recovery in ME/CFS patients compared to HC (p < 0.01). |
| Snell et al (2013) (98) | Observational case- control | Fukuda | 51 | 10 | 46.29 ± 8.01 | 40.80 ± 7.69 | 100% | 100% | 25.96 ± 4.95 | 28.99 ± 4.24 | 11.0 | Metabolic and Workload Measurements | Physical | Tertiary | Complex | Test 2 accuracy: 95.1% | Test 2 showed that at peak exercise and ventilatory or anaerobic threshold ME/CFS had significantly lower workload. These differences were able to characterise patients with an 95.1% accuracy (p < 0.001). Test 1 showed no significant difference. |

*= median (range). *Abbreviations: ASIC, Acid-sensing ion channel; ADCC, antibody-dependent cell-mediated cytotoxicity; blood-oxygen-level dependent imaging; COMT, catechol-O-methyltransferase; CSF, cerebral spinal fluid; CHRNA, cholinergic receptor nicotinic; CK, creatine kinase; DHEAs, dehydroepiandrosterone- sulfate; DPPIV, dipeptidyl peptidase IV; EV, extracellular vesicles; FABP-2, fatty acid-binding protein; FA, fractional anisotropy; fMRI, functional magnetic resonance imaging; intestinal; FGF, fibroblast growth factor; foxP3, forkhead box P3; GTP, Guanosine triphosphate; HC, healthy controls; HRV, heart-rate variability; HSP, heat shock protein; IFN, interferon; HexCer, hexosylceramides; IL, interleukin; iNKT, invariant NKT; IBS, irritable bowel syndrome; KLRG1, killer cell lectin-like receptor G1; lymphocyte function-associated antigen 1; MBP, major bacterial phyla; mRNA, messenger ribonucleic acid; miRNA, micro ribonucleic acid; PL, monounsaturated plasma phospholipids; ME/CFS, myalgic Encephalomyelitis/ chronic fatigue syndrome; NK, natural killer; NKCC, natural killer cell cytotoxicity; NT-proBNP, N-terminal pro b-type natriuretic peptide; PE, phosphatidylethanolamine; NPY, plasma neuropeptide Y; PEM, post-exertional malaise; AMP, pulse amplitude; P2X, purinergic receptor; PregS, pregnenolone sulfate; PSMA, proteasome subunits; RHI, reactive hyperaemia index; REFS, recursive ensemble feature selection; RT- PCR, reverse transcription polymerase chain reaction; RNAse, ribonuclease; STAT, signal transducers and activators of transcription; TORC, target of rapamycin; TLR4, Toll-like receptor 4; TRPC, transient receptor potential canonical; TRPM, Transient Receptor Potential Melastatin; TRPV, Transient receptor potential vanilloid; TNF, Tumour necrosis factor; VEGF-A, vascular endothelial growth factor A; WR, work rate*
