# Supplementary material for: Biomarkers for myalgic encephalomyelitis/chronic fatigue syndrome (ME/CFS): a systematic review
Source: BMC Med. 2023 May 24;21:189. doi: 10.1186/s12916-023-02893-9 (PMC10206551; doi:10.1186/s12916-023-02893-9)
Supplement: Supplementary file 3 — Additional file 3. JBI quality assessment table and descriptions [file 12916_2023_2893_MOESM3_ESM.docx]

**Additional file 3.** JBI quality assessment table and descriptions

|  | 1 | 2 | 3 | 4 | 5 | 6 | 7 | 8 | 9 | 10 |
| --- | --- | --- | --- | --- | --- | --- | --- | --- | --- | --- |
| Allen et al, 2012 | Y | N | N | Y | Y | Y | Y | Y | Y | N |
| Almenar-Pérez et al, 2020 | Y | N | Y | N/A | N/A | Y | Y | Y | N/A | Y |
| Armstrong et al, 2012 | Y | N | Y | N/A | N/A | Y | Y | Y | N/A | N |
| Blauensteiner et al, 2021 | N | N | Y | N/A | N/A | Y | Y | Y | N/A | Y |
| Bonilla et al, 2022 | Y | N | N | N/A | N/A | Y | Y | Y | N/A | N |
| Brenu et al, 2011 | U | Y | Y | N/A | N/A | Y | Y | Y | N/A | N |
| Brenu et al, 2012A | U | Y | Y | N/A | N/A | N | N | Y | N/A | N |
| Brenu et al, 2012B | U | N | Y | N/A | N/A | N | N | Y | N/A | Y |
| Brenu et al, 2014 | U | N | Y | N/A | N/A | Y | Y | Y | N/A | Y |
| Cabanas et al, 2018 | Y | N | N | N/A | N/A | Y | Y | Y | N/A | Y |
| Castro-Marrero et al, 2018 | Y | N | Y | N/A | N/A | Y | Y | Y | N/A | Y |
| Chacko et al, 2016 | Y | Y | Y | N/A | N/A | Y | Y | Y | N/A | Y |
| Ciregia et al, 2016 | N | Y | N | N/A | N/A | N | N | Y | N/A | N |
| Cliff et al, 2019 | N | N | Y | N/A | N/A | Y | Y | Y | N/A | N |
| de Vega et al, 2017 | Y | Y | Y | N/A | N/A | Y | Y | Y | N/A | N |
| Domingo et al, 2021 | Y | Y | Y | N/A | N/A | Y | Y | Y | N/A | N |
| Eaton-Fitch et al, 2022 | Y | Y | Y | N/A | N/A | Y | Y | Y | N/A | Y |
| Eguchi et al, 2020 | Y | Y | Y | N/A | N/A | Y | Y | Y | N/A | N |
| Escorihuela et al, 2020 | Y | Y | Y | N/A | N/A | Y | Y | Y | N/A | N |
| Esfandyarpour et al, 2019 | N | N | Y | Y | Y | Y | Y | Y | Y | N |
| Espinosa et al | N | N | N | N/A | N/A | N | N | Y | N/A | N |
| Fenouillet et al, 2016 | N | N | Y | Y | Y | Y | Y | Y | Y | N |
| Fletcher et al, 2009 | Y | N | Y | N/A | N/A | Y | Y | Y | N/A | N |
| Fletcher et al, 2010A | Y | N | Y | N/A | N/A | Y | Y | Y | N/A | N |
| Fletcher et al, 2010B | U | N | Y | N/A | N/A | Y | Y | Y | N/A | Y |
| Frith et al, 2012 | Y | N | Y | Y | Y | Y | Y | Y | Y | N |
| Fukuda et al, 2016 | U | N | N | Y | N | Y | N | Y | Y | N |
| Gao et al, 2013 | Y | N | N | Y | Y | Y | Y | Y | Y | U |
| Germain et al, 2017 | Y | N | N | N/A | N/A | N | N | Y | N/A | N |
| Germain et al, 2018 | Y | N | N | N/A | N/A | N | Y | Y | N/A | Y |
| Gow et al, 2009 | Y | Y | Y | N/A | N/A | Y | Y | Y | N/A | N |
| Gravelsina et al, 2021 | U | N | N | N/A | N/A | N | N | Y | N/A | Y |
| Groven et al, 2020 | U | N | N | N/A | N/A | Y | Y | Y | N/A | Y |
| Günther et al, 2019 | Y | N | N | N/A | N/A | N | N | Y | N/A | N |
| Haffke et al, 2022 | U | N | Y | N/A | N/A | Y | Y | Y | N/A | Y |
| Halpin et al, 2017 | Y | N | Y | N/A | N/A | Y | Y | Y | N/A | N |
| Hanevik et al, 2012 | N | N | Y | N/A | N/A | Y | Y | Y | N/A | Y |
| Hardcastle et al 2015A | Y | Y | Y | N/A | N/A | Y | Y | Y | N/A | Y |
| Hardcastle et al 2015B | Y | Y | Y | N/A | N/A | Y | Y | Y | N/A | Y |
| Hornig et al, 2015 | Y | Y | N | N/A | N/A | Y | Y | Y | N/A | Y |
| Iacob et al, 2016 | N | N | Y | N/A | N/A | Y | Y | Y | N/A | N |
| Jawad Kadhum et al | Y | N | N | N/A | N/A | N | N | Y | N/A | N |
| Khaiboullina et al, 2015 | Y | N | N | N/A | N/A | N | N | Y | N/A | N |
| Kitami et al, 2020 | Y | N | N | N/A | N/A | Y | Y | Y | N/A | Y |
| Landi et al, 2016 | Y | Y | Y | N/A | N/A | Y | Y | Y | N/A | Y |
| Lidbury et al, 2017 | U | Y | Y | Y | Y | Y | Y | Y | Y | N |
| Lidbury et al, 2019 | U | Y | Y | Y | Y | Y | Y | Y | Y | N |
| Light et al, 2009 | Y | N | N | Y | Y | Y | Y | Y | Y | N |
| Light et al, 2012 | N | N | Y | Y | Y | Y | Y | Y | Y | N |
| Maes et al, 2005 | U | N | N | N/A | N/A | Y | Y | Y | N/A | N |
| Maes et al, 2011 | Y | N | N | N/A | N/A | Y | Y | Y | N/A | N |
| Maes et al, 2012 | Y | Y | Y | NA | N/A | Y | Y | Y | N/A | N |
| Maes et al, 2015 | Y | N | Y | N/A | N/A | Y | Y | Y | N/A | N |
| Maher et al, 2005 | Y | N | Y | N/A | N/A | N | N | Y | N/A | N |
| Mandarano et al, 2018 | Y | N | Y | N/A | N/A | Y | Y | Y | N/A | Y |
| Marshall-Gradisnik et al 2016A | Y | N | Y | N/A | N/A | Y | Y | Y | N/A | N |
| Marshall-Gradisnik et al 2016B | Y | N | Y | N/A | N/A | Y | Y | Y | N/A | Y |
| Mathew et al, 2009 | Y | N | Y | N/A | N/A | Y | Y | Y | N/A | N |
| Melvin et al, 2019 | Y | N | Y | N | Y | Y | Y | Y | Y | N |
| Metselaar et al, 2021 | U | N | N | N/A | N/A | N | N | Y | N/A | N |
| Milivojevic et al, 2020 | Y | Y | Y | N/A | N/A | Y | Y | Y | N/A | Y |
| Missailidis et al, 2020 | Y | N | Y | N/A | N/A | Y | Y | Y | N/A | Y |
| Nacul et al, 2018 | N | N | Y | Y | Y | Y | Y | Y | Y | N |
| Nacul et al, 2019 | U | N | N | N/A | N/A | Y | Y | Y | N/A | Y |
| Nagy-Szakal et al, 2017 | Y | Y | Y | N/A | N/A | Y | Y | Y | N/A | Y |
| Nagy-Szakal et al, 2018 | Y | Y | Y | N/A | N/A | Y | Y | Y | N/A | Y |
| Natelson et al, 2007 | Y | N | Y | N/A | N/A | Y | Y | Y | N/A | Y |
| Natelson et al, 2017 | Y | N | Y | N/A | N/A | Y | Y | Y | N/A | Y |
| Nelson et al, 2019 | U | U | Y | Y | Y | Y | Y | Y | Y | N |
| Nelson et al, 2021 | U | U | Y | Y | Y | Y | Y | Y | Y | N |
| Nepotchatykh et al, 2020 | Y | N | Y | Y | Y | N | N | Y | Y | N |
| Nguyen et al, 2017 | N | Y | Y | N/A | N/A | Y | Y | Y | N/A | Y |
| Nijs et al, 2010 | Y | N | Y | Y | Y | Y | Y | Y | Y | N |
| Nkiliza et al, 2021 | Y | N | Y | N/A | N/A | Y | Y | Y | N/A | N |
| Okada et al, 2004 | N | U | N | N/A | N/A | Y | Y | Y | N/A | Y |
| Petty et al, 2016 | Y | N | Y | N/A | N/A | Y | Y | Y | N/A | N |
| Powell et al, 2003 | U | N | N | N/A | N/A | Y | Y | Y | N/A | N |
| Provenzano et al, 2020 | Y | N | N | Y | Y | Y | Y | Y | Y | N |
| Rayhan et al, 2021 | U | N | Y | Y | Y | Y | Y | Y | Y | U |
| Rivas et al, 2018 | Y | N | Y | N/A | N/A | N | N | Y | N/A | Y |
| Saiki et al, 2008 | Y | N | U | N/A | N/A | Y | Y | Y | N/A | N |
| Shan et al, 2018 | N | N | N | Y | Y | Y | Y | Y | Y | N |
| Shishioh-Ikejima et al, 2010 | Y | N | Y | N/A | N/A | N | N | Y | N/A | Y |
| Shukla et al, 2015 | Y | Y | Y | Y | Y | Y | Y | Y | Y | Y |
| Simonato et al, 2021 | Y | N | Y | N/A | N/A | Y | N | Y | N/A | N |
| Singh et al, 2016 | U | N | N | N/A | N/A | N | N | Y | N/A | N |
| Snell et al, 2013 | Y | N | Y | Y | N | N | N | Y | Y | N |
| Sorensen et al, 2003 | Y | N | N | Y | Y | Y | Y | Y | Y | Y |
| Sørland et al, 2021 | U | N | N | N/A | N/A | Y | Y | Y | N/A | N |
| Stringer et al, 2013 | Y | N | Y | N/A | N/A | Y | Y | Y | N/A | Y |
| Sung et al, 2020 | Y | Y | Y | N/A | N/A | N | N | Y | N/A | N |
| Sweetman et al, 2020 | Y | N | Y | N/A | N/A | Y | N | Y | N/A | N |
| Szklarski et al, 2021 | N | N | Y | N/A | N/A | Y | Y | Y | N/A | Y |
| Thambirajah et al, 2008 | Y | N | Y | Y | Y | Y | Y | Y | Y | N |
| Thapaliya et al, 2021 | Y | Y | Y | N/A | N/A | Y | Y | Y | N/A | Y |
| Theorell et al, 2017 | U | Y | N | N/A | N/A | N | N | Y | N/A | N |
| Tiev et al, 2003 | Y | N | Y | N/A | N/A | Y | Y | Y | N/A | N |
| Tokunaga et al, 2020 | Y | Y | N | N/A | N/A | Y | Y | Y | N/A | N |
| Vernon et al, 2005 | Y | Y | N | N/A | N/A | Y | Y | Y | N/A | N |
| White et al, 2012 | Y | N | N | Y | Y | Y | Y | Y | Y | N |
| Zeineh et al, 2015 | Y | N | Y | N/A | N/A | Y | Y | Y | N/A | N |

Supplementary Table: The Joanna Briggs Institute Checklist for Case Control Studies. Items answered as not applicable were removed from the final percentage. Abbreviations: JBI, Joanna Briggs Institute; Y, Yes; N, No; N/A, not applicable; U, unclear.

JBI Checklist items:
1. Were the groups comparable other than the presence of disease in cases or the absence of disease in controls?
2. Were cases and controls matched appropriately?
3. Were the same criteria used for identification of cases and controls?
4. Was exposure measured in a standard, valid and reliable way?
5. Was exposure measured in the same way for cases and controls?
6. Were confounding factors identified?
7. Were strategies to deal with confounding factors stated?
8. Were outcomes assessed in a standard, valid and reliable way for cases and controls?
9. Was the exposure period of interest long enough to be meaningful?
10. Was appropriate statistical analysis used?

**Allen et al, 2012**

1. Yes, Patients and HC were matched for age, sex, and BMI.
2. No, Source population information not provided.
3. No, ME/CFS patients met the Fukuda criteria. Healthy controls were sedentary. Criteria by which HC were selected was not described.
4. Yes, Exposure was orthostatic tilt. This comprised of a 10-minute resting baseline measurement that was followed by a 70◦controlled head up tilt for three minutes before returning to original position.
5. Yes, exposure was measured consistently between patients and HC.
6. Medications and fitness levels were identified as a potential confounding variables.
7. Yes, those who took potentially interfering medications were excluded and only sedentary HC were recruited.
8. Yes, outcomes were assessed using optical multi-site photoplethysmography.
9. Yes, the exposure period showed an effect.
10. No, tilt changes were assessed using Student’s *t*-test. Normality was not assessed. Contingency tables to summarise group classifications, diagnostic accuracy, diagnostic sensitivity, specificity, negative predictive value, and positive predictive value were produced. Cluster analysis was also performed, optimal linear separation of groups was calculated.

**Almenar-Perez et al, 2020**

1. Yes, all participants were women and aged matched.
2. Yes, participants were obtained from the UK ME/CFS Biobank.
3. Yes, ME/CFS patients met Fukuda and CCC criteria. The following exclusion criteria applied to both ME/CFS patient and HC: use of antivirals in the past three months, had any history of acute or chronic infectious diseases, had a mood disorder, pregnant or breastfeeding in the last 12 months, were morbidly obese (BMI ≥ 40).
4. N/A. No exposure. Study investigated baseline microRNAs from PBMCs and extracellular vesicles in ME/CFS patients compared to HC.
5. N/A. As above.
6. Yes, potential confounding variables identified include the use of antivirals in the past 3 months, a history of acute or chronic infectious diseases, a mood disorder, pregnant or breastfeeding in the last 12 months or were morbidly obese.
7. Yes, confounding variables were mitigated via exclusion.
8. Yes, outcomes were measured using miRNA profiling assays and clinically validated methodologies to assess plasma RV features.
9. N/A. No exposure.

Yes, normality was assessed using Shapiro-Wilk test. T-student or Mann-Whitney test were used depending on whether data distribution was normal or not. **Armstrong et al, 2012**

1. Yes, participants were age and sex- matched.
2. No, no demographic information was provided.
3. Yes, ME/CFS patients were diagnosed according to the CCC criteria. HC did not report symptoms of fatigue. Participants were not related- or living together.
4. N/A. No exposure. This study investigates metabolite differences in ME/CFS patients compared to HC using NMR metabolic profiling.
5. N/A. No exposure.
6. Yes, confounding factors include large concentration of glucose (consistent with insulin- resistant disorder).
7. Yes, confounding factors were mitigated through exclusion.
8. Yes, Nuclear Magnetic Resonance was used.
9. N/A. No exposure.
10. No, Data was assessed for normality. Data was log-transformed. Log-transformation is not sufficient to adjust for skewness of data.

**Blauensteiner et al, 2021**

1. No. Patients were aged matched but not sex matched. BMI did not significantly differ.
2. No, Source population information not provided.
3. Yes, ME/CFS patients were diagnosed according to the CCC and Fukuda criteria. Exclusion criteria include: (1) used drugs that altered immune function or antiviral medications in the past three months. (2) had vaccinations in the last three months. (3) had experience of acute infectious disease Hepatitis B and C or tuberculosis. (4) had another severe illness such as cancer, heart disease, or diabetes (5) severe mood disorder (6) pregnant or breast feeding in the last 12 months or severely obese.
4. N/A, no exposure. Study investigated altered endothelial dysfunction-related miRs in ME/CFS patients compared to HC.
5. As above.
6. Yes, confounding variables identified include those reported in [3].
7. Yes, confounding variables were controlled through exclusion criteria.
8. Yes, real-time PCR and clinical data was used
9. N/A. No exposure.
10. Yes, statistical analysis was conducted using R software. A correlation matrix was produced. The tool “MASS” was used to adjust for multiple comparisons.

**Bonilla et al, 2022**

1. Yes, ME/CFS patients were age- and sex- matched.
2. No, no demographic information was supplied.
3. No, patients were diagnosed according to Fukuda criteria, however, no criteria was provided for HC.
4. N/A no exposure. This study investigates the role of extracellular vesicles in the pathology of ME/CFS.
5. N/A. No exposure.
6. Yes, confounding factors include active or uncontrolled morbidities that make patients unable to participate such as conditions or medications that may cause immunosuppression or immunodeficiency.
7. Yes, confounding factors were mitigated through exclusion.
8. Yes, flow cytometry was used.
9. N/A. No exposure.
10. No, one- way ANOVA with adjustments for multiple comparisons were made, however, it was unclear whether justification of tests were made based on normality tests.

**Brenu et al, 2011**

1. Unclear, age- matched, however, it is unclear if sex matched.
2. Yes, participants were recruited from Queensland and New South Wales.
3. Yes, ME/CFS patients were diagnosed according to the Fukuda criteria. Participants previously diagnosed with autoimmune disorders, psychosis, epilepsy, heart disease, or who were pregnant or breastfeeding were excluded.
4. N/A. No exposure, this study assesses immunological markers in ME/CFS patients compared to HC.
5. N/A. No exposure.
6. Yes, possible confounding factors include: autoimmune disorders, psychosis, epilepsy, heart disease.
7. Yes, confounding variables were mitigated via exclusion.
8. Yes, flow cytometry was used.
9. N/A. No exposure.
10. No, while appropriate considerations were made on sample size, the authors did not assess for normality or adjust for multiple comparisons.

**Brenu et al, 2012A**

1. Unclear, if age or sex-matched.
2. Yes, participants were recruited from Queensland and New South Wales. HC were recruited from similar locations to ME/CFS patients.
3. Yes, ME/CFS patients were diagnosed according to the Fukuda criteria. HC were non-fatigued. Participants previously diagnosed with autoimmune disorders, psychosis, epilepsy, heart disease were excluded.
4. N/A. No exposure, this study assesses longitudinal changes in NK cell cytotoxicity in ME/CFS patients compared to HC.
5. N/A. No exposure.
6. No, possible confounding factors include: autoimmune disorders, psychosis, epilepsy, heart disease were identified. Other confounding variables may arise from blood collections at different time points
7. No, some confounding variables were mitigated via exclusion. Confounding variables that arise from blood collections at different time points were not mitigated: including testing at similar sites – although they provided some justification of this it did not control for added variability in data.
8. Yes, flow cytometry was used.
9. N/A. No exposure.
10. No, Although variances in data (that deviate from sphericity has been conducted) as well as adjustment for multiple comparisons – Correlations for comparisons should have used the same test (either Spearman or Pearson and this decision should have been made based on normality and applied in the selection of other tests such as ANOVA or Kruskal – Wallis test.

**Brenu et al, 2012B**

1. Unclear, if age- or sex- matched.
2. No, No demographic information was suppled.
3. Yes, ME/CFS patients were diagnosed according to the Fukuda criteria. HC had no medical history or symptoms of prolong fatigue.
4. N/A. No exposure, This study investigated cytotoxic lymphocyte microRNAs in ME/CFS patients compared to HC.
5. N/A. No exposure.
6. No, no confounding factors were identified.
7. No, as above.
8. Yes, flow cytometry was used.
9. N/A. No exposure.
10. Yes. A non-parametric mann-whitney U test was used.

**Brenu et al, 2014**

1. Unclear, aged- matched but unclear if sex- matched.
2. No, study states that participants were recruited from a South-East Queensland patient database, no mention of where HC were sourced.
3. Yes, ME/CFS patients were diagnosed according to the Fukuda criteria. HC had no medical history or symptoms of prolong fatigue. Exclusion criteria includes: smokers, pregnant/breast-feeding, individuals with autoimmune, thyroid or cardiac related disorders prior to the onset of ME/CFS.
4. N/A. No exposure, this study investigated plasma microRNAs in ME/CFS patients compared to HC using high-throughput sequencing.
5. N/A. No exposure.
6. Yes, confounding factors include: smokers, pregnant/breast-feeding, individuals with autoimmune, thyroid or cardiac related disorders prior to the onset of ME/CFS.
7. Yes, confounding factors were mitigated through exclusion.
8. Yes, Illumina high throughput Hiseq2000 sequencing was used
9. N/A. No exposure.
10. Yes. A non-parametric mann-whitney U test was used.

**Cabanas et al, 2018**

1. Yes, ME/CFS patients were age- and sex- matched
2. No, no demographic information was supplied.
3. No, All patients were diagnosed by the CCC criteria. No criteria was provided HC.
4. N/A. No exposure. This study investigated calcium signalling through TRPM3 channels in ME/CFS patients compared to HC. Cells were exposed to ononetin and preg-s in order to understand the physiology and cell function, however, the study was observational and these were not provided as an intervention.
5. N/A. As above.
6. Yes, confounding factors include history of smoking, autoimmune diseases, cardiac diseases, diabetes or other co-morbidities. Use of pharmacological agents that directly or indirectly influence TRPM3 or Ca^2+^ signalling.
7. Yes, confounding factors were mitigated via exclusion. No participant used any pharmaceuticals that in directly or indirectly interact with TRPM3 channels.
8. Yes, whole- cell patch clamp was used.
9. Yes, please refer to [4]
10. Yes, normality was considered using the Shapiro- Wilk test. Mann-Whitney U and Fisher’s exact test were used to determine significant differences.

**Castro-Marrero et al, 2018**

1. Yes, patients were age, sex and ethnicity matched.
2. No, Source information provided for ME/CFS patients but not HC.
3. Yes, ME/CFS patients fulfilled the Fukuda definition. Exclusion criteria for participants included those with comorbid psychiatric illness, endocrino-metabolic, haematological, and autoimmune disorders, CVD, pregnancy, or breast feeding, drug abuse and smoking.
4. N/A, no exposure. Study aimed to characterise extracellular vesicles in ME/CFS.
5. N/A, As above.
6. Yes, confounding variables identified include those reported in [3].
7. Yes, confounding variables were controlled through exclusion criteria.
8. Yes, Western blot, nanoparticles tracking analysis, and lateral flow immunoassay was used.
9. N/A. No exposure.
10. Yes. Study was conducted using a Mann-Whitney U test following assessment of normality.

**Chacko et al, 2016**

1. Yes, Participants were age- and sex- matched.
2. Yes, all participants were of European descent and were residents of Australia at the time of blood collection.
3. Yes, ME/CFS patients were defined according to ICC criteria. Dr Bell’s disability scale was used to determine severity: Patients categorised as moderate ME/CFS scored > 30%. Severe ME/CFS patients scored < 30% and were considered housebound or bedridden. HC were defined as non-fatigued. Any participant that had an alternative explanation for their symptoms were excluded. All participants were excluded if they had the following: any alterative disease that explains symptoms or autoimmune disorder, multiple sclerosis, psychosis, major depression, heart disease, thyroid related disorder or were pregnant, breast feeding or smoking.
4. N/A. There was no exposure. This study investigated the role of protein kinase genes in the pathology of ME/CFS.
5. N/A. No exposure.
6. Yes any alterative disease that explains symptoms or autoimmune disorder, multiple sclerosis, psychosis, major depression, heart disease, thyroid related disorder or were pregnant, breast feeding or smoking.
7. Yes, these confounding factors were mitigated through exclusion.
8. Yes, nanostring was used.
9. N/A no exposure.
10. Yes, normality was determined using Shapiro Wilk normality test. Kruskal- Wallis test was used to determine significance.

**Ciregia et al, 2016**

1. No, Participants were age and demographic matched. No mention as to whether participants were sex matched.
2. Yes, ME/CFS patients and HC controls were matched based on similar demographic characteristics.
3. No, ME/CFS patients were classified according to the Fukuda criteria. HC recruitment criteria was not defined.
4. N/A, no exposure. This study investigates differential expression of proteins in ME/CFS using bottom-up proteomics.
5. N/A, as above.
6. No, no potential confounding factors were identified.
7. No, As above.
8. Yes, bottom-up proteomics was used.
9. N/A, no exposure.
10. No, Mann- Whitney U test was used for non-normal data. No mention of adjustments for multiple comparisons for logistic and linear regression was made.

**Cliff et al, 2019**

1. No, ME/CFS patients were sex matched but not age- matched.
2. No, no source information was provided.
3. Yes, ME/CFS patients met CCC and/or Fukuda criteria. All participants were excluded if they took antiviral medication or drugs, had vaccinations in the preceding three months, had a history of acute and chronic infectious diseases such as hepatitis B and C, tuberculosis, HIV, had other chronic conditions such as cancer, had a severe mood disorder or has been pregnant or breastfeeding in the past 12 months or had a morbidly high BMI.
4. N/A. Study investigated cellular immune function in ME/CFS patients.
5. N/A. As above.
6. Yes, confounding variables identified include those reported in [3].
7. Yes, confounding variables were controlled through exclusion criteria. Data analysis was conducted blinded.
8. Yes, serology data was collected. Cell-based assays were also conducted.
9. N/A. No exposure.
10. No, data was log-transformed. Justification was that this was done due to convenience rather than opting for a non-parametric test for skewed data which would have been more appropriate.

**de Vega et al, 2017**

1. Yes, age and sex matched
2. Yes, blood was collected from one of four collection sites in USA and all participants were Caucasian.
3. Yes, ME/CFS patients met Fukuda and Canadian Consensus Criteria. HC: tested negative for HIV, AIDS, and/ or Hepatitis C, were non-obese.
4. N/A, no exposure. Study investigated epigenetic modifications and glucocorticoid sensitivity.
5. N/A, As above.
6. Yes, confounding variables identified include those reported in [3]. Potential interference of DNA methylome and immune response.
7. Yes, confounding variables were controlled through exclusion criteria.
8. Yes, DNA methylome was analysed using Illumina HumanMethylation450 BeadChip Array. Glucocorticoid sensitivity was assessed by stimulating PBMCs with phytohaemagglutinin and suppressed growth with dexamethasone.
9. N/A. No exposure.
10. No, Assays were conducted in triplicate. Selected a parametric, Pearson correlation test but their output was a non-parametric Wilcoxon-rank sum.

**Domingo et al, 2021**

1. Yes, participants were age, sex, BMI and demographic matched
2. Yes, participants were from the same geographical area and were all Caucasian descent.
3. Yes, ME/CFS patients met Fukuda case definition and all patients were diagnosed by an ME/CFS specialist. All participants followed strict exclusion criteria: No previous or current diagnosis of acute infectious diseases in the previous four weeks, autoimmune disorders, multiple sclerosis, psychosis, major depression, heart disease, haematological disorders, sleep apnoea, or thyroid-related disorders, pregnancy or breast feeding, smoking habit, strong hormone- related medications.
4. N/A, No exposure. This study investigates the role of protein N-terminal prohormone of brain natriuretic peptide and fibroblast growth factor 21 in ME/CFS patients compared to HC.
5. N/A. As above.
6. Yes, confounding variables identified include those reported in [3].
7. Yes, confounding variables were controlled through exclusion criteria.
8. Yes, immunoassays and standardised protocols were used to determine cytokine concentration, C-reactive protein and FGF21 and NT-proBNP.
9. N/A. No exposure.
10. No, Normality test was conducted and Mann- Whitney U test was selected. A Pearson’s correlation test was used on a non-normal dataset.

**Eaton-Fitch et al, 2022**

1. Yes, ME/CFS patients were age- and sex- matched.
2. Yes, all collections were conducted within South-East Queensland and NSW (Tweed area).
3. Yes, ME/CFS patients met the CCC or ICC criteria. HC have not been diagnosed with any underlying illness and are non-fatigued.
4. N/A. There was an intervention included in this study, however, it is not applicable to our systematic review as we are not reporting on interventions.
5. N/A. See [4].
6. Yes, confounding variables include history of alcohol abuse, cardiovascular disease, diabetes, metabolic syndrome, thyroid disease, malignancies, insomnia. Use of pharmacological agents that directly or indirectly interfere with TRPM3 ion channel function were also considered confounding factors.
7. Yes, Confounding factors were mitigated through exclusion.
8. Yes, calcium influx was measured through imaging via confocal microscopy
9. N/A.
10. Yes, normality was measured using the Shapiro- Wilk test with further validation using observation of histogram plots. A non-parametric Mann-Whitney U test was selected based on the results.

**Eguchi et al, 2020**

1. Yes, participants were matched in age, gender, weight, and BMI.
2. No, No demographic information was provided. Only that patients attended the Osaka outpatient clinic.
3. Yes, ME/CFS patients were diagnosed according to the Fukuda and CCC criteria. Exclusion criteria for all participants include those with: neuroinflammatory or immune disorders, active medical condition that could explain presence of chronic fatigue, diagnosable illness that isn’t resolved, alcohol or substance abuse, severe obesity, pregnancy or breast feeding.
4. N/A. No exposure. This study investigated differences in circulating extracellular vesicles in ME/CFS patients compared to HC.
5. N/A. No exposure.
6. Yes, confounding variables include: neuroinflammatory or immune disorders, active medical condition that could explain presence of chronic fatigue, diagnosable illness that isn’t resolved, alcohol or substance abuse, severe obesity, pregnancy or breast feeding.
7. Yes, confounding variables were mitigated via exclusion.
8. Yes, protein composition in circumating EV were assessed using nanoLC-MS/MS. EV size was measured using transmission electron microscopy. Flow cytometry was used to count EVs.
9. N/A. No exposure
10. No, differences between groups were assessed either by the Mann-Whitney U test or Kruskal-Wallis test. Correlations were determined using Spearman rank-sum test. Proteomics were transformed – justification was provided. No adjustments for multiple comparisons was described.

**Escorihuela et al, 2020**

1. Yes, participants were age, sex, BMI and demographic matched
2. Yes, participants were from the same geographical area and were all Caucasian descent.
3. Yes, ME/CFS patients met Fukuda case definition. Exclusion criteria include: previous or current diagnosis of autoimmune illnesses, multiple sclerosis, psychosis, major depression, cardiovascular disorders, haematological disorders, infectious diseases, sleep apnea or thyroid-related illnesses, pregnancy or breast-feeding, smoking, hormone-related drugs or any symptoms that might be confused with ME/CFS.
4. N/A, no exposure. This study investigated heart rate variability in ME/CFS patients compared to HC.
5. N/A. As above.
6. Yes, confounding factors include having an active lifestyle, autoimmune illnesses, multiple sclerosis, psychosis, major depression, cardiovascular disorders, haematological disorders, infectious diseases, sleep apnea, or thyroid related illnesses, pregnancy or breast-feeding, smoking, hormone-related drugs or any associated symptoms of ME/CFS.
7. Yes, Confounding factors were mitigated by exclusion
8. Yes, HRV recording-resting state tests were conducted.
9. N/A. No exposure.
10. No, Normality distribution was taken into consideration where student t test was used for independent samples and non-parametric Mann Whitney U test was used for non-normal datasets. Regression was conducted, however, no adjustments for multiple comparisons was conducted.

**Esfandyarpour et al, 2019**

1. No, there were 5 HC that were age- and-sex- matched with ME/CFS patients, however, an additional 15 HC participated in the study.
2. No, no demographic information was provided.
3. Yes, ME/CFS patients were diagnosed according to the CCC criteria. HC were not diagnosed with ME/CFS or related diseases and had no blood relatives diagnosed with ME/CFS.
4. Yes, the exposure to the cells was hyperosmotic stressors. This exposure was measured in a standard and reliable way. Using sensors, three parameters of in-phase impedance (Zre), out-of-phase impedance (Zim), and impedance magnitudes were recorded and calculated to further enhance the precision and accuracy of the sensors.
5. Yes, all protocols were identically conducted in ME/CFS patients and HC.
6. Yes, being blood-related was identified as a potential confounding factor.
7. Yes, confounding factor was mitigated through exclusion.
8. Yes, electrochemical impedance spectroscopy was used.
9. Yes the exposure time was sufficient to see a response.
10. No, All P- values were calculated using welch’s T test. It is unclear whether data distribution and normality were considered.

**Espinosa et al, 2019**

1. No, It was unclear whether age or sex matched.
2. No, patient demographics were not provided.
3. No, ME/CFS patients met CCC criteria. There was no criteria given for healthy controls.
4. N/A, no exposure. This study investigated protein expression in T- lymphocytes.
5. As above.
6. No, potential confounding variables were not identified.
7. No, No article did not mitigate for confounding factors
8. Yes, flow cytometry was conducted.
9. N/A. No exposure.
10. Yes, Mann Whitney U test was conducted. ROC curves were also carried out.

**Fenouillet et al, 2016**

1. No, Patients were age-matched but not sex matched.
2. No, Patient demographics were not provided.
3. Yes, ME/CFS patients met IOMC criteria. Healthy controls did not have any symptoms of ME/CFS – healthy controls underwent medical examination.
4. Yes, incremental exercise at maximal V0_2_ value. V0_2_ max (based on maximal heart rate), M-wave recordings.
5. Yes, Exposure was measured consistently between patients and healthy controls
6. Yes, Patients with other medically explained reasons for fatigue
7. Yes, authors mitigated confounding variables by exclusion.
8. Yes, Clinically validated tools including blood tests and London Handicap Scale (LHS) and the Medical Outcome Study Short Form-36 (SF-36) were used.
9. Yes the exposure period showed an effect.
10. No correction for multiple comparisons.

**Fletcher et al, 2009**

1. Yes, participants were age matched and were all women.
2. No, patient demographics were not provided.
3. Yes, ME/CFS patients met Fukuda case definition of ME/CFS. HC had no medical or psychiatric conditions: heart disease, COPD. Malignancy or systemic disorders. No alcohol or substance abuse.
4. N/A, this study has no exposure. This study investigates the role of plasma cytokines in ME/CFS pathology.
5. N/A, no exposure.
6. Yes, confounding factors include medical or psychiatric conditions, alcohol or substance abuse.
7. Yes, confounding variables were mitigated through exclusion.
8. Yes, A quantitative ELISA based test was used to measure cytokines.
9. N/A. No exposure.
10. No, normality was assessed and a non-parametric Kruskal- Wallis test, however, no adjustment for multiple comparisons were made.

**Fletcher et al, 2010A**

1. Yes, participants were age- and sex- matched
2. No, patient demographics were not provided.
3. Yes, ME/CFS patients met Fukuda case definition of ME/CFS. All participants had no medical or psychiatric conditions: immunomodulatory or antibiotic medication,. No alcohol or substance abuse within two years.
4. N/A, this study has no exposure. This study investigates the role of PeptidaseIV/CD26 and NK cell function in ME/CFS pathology.
5. N/A, no exposure.
6. Yes, confounding factors include medical or psychiatric conditions, immunomodulatory medications alcohol or substance abuse. Confounding factors also included timing of experimentation.
7. Yes, confounding variables were mitigated through exclusion. Delivery of blood to the laboratory was within 4 hours.
8. Yes, A quantitative ELISA was used to measure Dipeptidyl PeptidaseIV/CD26 and NK cell function was measured using flow cytometry was used.
9. N/A. No exposure.
10. No, normality was assessed and a non-parametric Kruskal- Wallis test, however, no adjustment for multiple comparisons were made.

**Fletcher et al, 2010B**

1. U, It was unclear whether patients were age- or sex- matched
2. No, patients were drawn from the University of Miami Miller school of Medicine CFS and Immunodeficiency Clinic. Demographics for HC was not reported.
3. Yes, ME/CFS patients met Fukuda case definition of ME/CFS. Healthy controls were sedentary. HC had no medical or psychiatric conditions or take immunostimulatory medications. No alcohol or substance abuse.
4. N/A, this study has no exposure. This study investigates the role of Plasma neuropeptide Y in ME/CFS pathology.
5. N/A, no exposure.
6. Yes, confounding factors include medical or psychiatric conditions or certain medications, alcohol or substance abuse.
7. Yes, confounding variables were mitigated through exclusion.
8. Yes plasma neuropeptide Y was measured using competitive radioimmunoassay (RIA)
9. N/A. No exposure.
10. Yes, Mann Whitney U test was selected for independent comparisons between psychosocial measures and plasma NPY. ROC comparisons were used to calculate specificity and sensitivity.

**Frith et al, 2012**

1. Yes, patients were age and sex-matched with healthy controls.
2. No, Patients attended the same Northern Regional ME/CFS clinic. The patient demographic of the healthy controls were not provided.
3. Yes, ME/CFS patients met Fukuda criteria. HC were sedentary, were not taking any conflicting medications of haemodynamic analysis, not diabetic or have renal disease.
4. Yes, The exposure is standing via tilt-table test.
5. Yes, The duration of supine/ standing was the same for ME/CFS patients and healthy controls.
6. Yes, Confounding variables identified includes: conflicting medications, other medical conditions. Alcohol, smoking, caffeine, intensive physical exercise.
7. Conflicting medications and medical conditions were mitigated using exclusion. Participants were requested to refrain from alcohol, smoking, caffeine, intensive physical exercise to control for potential confounding variables.
8. Yes, a high-tech device—task force monitor was used.
9. Yes, the time was sufficient to see a response.
10. No, Students T test was exclusively used, however, non-parametric data was present.

**Fukuda et al, 2016**

1. Unclear, HC came from a pool that were age- and sex- matched, however, they were separated into groups for different experiments and it was unclear if the individual groups were age- and sex- matched.
2. No, no demographical information was provided.
3. No, ME/CFS patients were diagnosed according to the Fukuda criteria. No criteria was provided for HC.
4. Yes, at a sub-acute task. Exposure was measured using clinical standard pathology measurements.
5. No, the exposure was measured in all ME/CFS patients while the HC were broken into three groups where they only received one exposure.
6. Yes, confounding variables identified includes: neuroinflammatory or immune disorders, any active medical condition, alcohol or substance abuse, pregnancy
7. No, it was unclear whether these confounding factors were mitigated by exclusion in HC it was only noted in ME/CFS patients. Also other conditions such as Fibromyalgia and major depressive disorder were not a criteria for exclusion, however, no mention of mitigation of potential confounding effects were mentions. T-tests were conducted to see if there were significant influence, however, no adjustments or approach to mitigate the results were made.
8. Yes, clinical blood collections analysed by pathology centres were collected.
9. Yes, time was sufficient to see a response.
10. No, Correlations were measured using Pearsons correlation. T-tests were also used to see if there are any significant differences between sex influenced differences in d-ROM, BAP and OSI and whether medications and comorbidity significantly influenced findings.

**Gao et al, 2013**

1. Yes, age, sex, race, and BMI- matched.
2. No, although they provided information on race, they did not provide any indication on geographical location.
3. No, Fukuda criteria was used to diagnose ME/CFS patients. No criteria was provided for HC.
4. Yes, HRV was measured in a standard and reliable way.
5. Yes, HRV was measured in the same way for ME/CFS and HC.
6. Yes, confounding factors include those who experience arrythmias or those who did not have interpretable data.
7. Yes, confounding factors were mitigated via exclusion
8. Yes, heart rate data was collected using biopac feeds on somnologica software.
9. Yes, time was sufficient to see a response.
10. Unclear, Two- sided T test was used. Unclear whether selection of test took into consideration distribution of data.

**Germain et al 2017**

1. Yes, participants were age, sex and BMI matched.
2. No, patient demographics were not provided.
3. No, All patients met Fukuda criteria but inclusion/ exclusion criteria for ME/CFS patients and healthy controls were not provided.
4. N/A, no exposure. Study assessed metabolic differences in ME/CFS patients and healthy controls using metabolic profiling.
5. N/A. As above.
6. No. No confounding variables were identified.
7. No, As above.
8. Yes, Mass spectrometry was conducted using validated methodology.
9. N/A. No exposure.
10. No information on statistical methods were provided.

**Germain et al 2018**

1. Yes, patients were age, sex and BMI matched.
2. No, patient demographics were not provided.
3. No, All patients met Fukuda criteria but inclusion/ exclusion criteria for ME/CFS patients and healthy controls were not provided.
4. N/A, no exposure. Study assessed metabolic differences in ME/CFS patients and healthy controls using metabolic profiling.
5. N/A. As above.
6. No. Suggestions to reduce variables were provided, however, the potential variables were not outlined.
7. Yes, the only metabolites that were used in this study where the ratio of the mean for patients to the mean for controls was either above 1.1 or below 0.9.
8. Yes, Mass spectrometry was conducted using validated methodology.
9. N/A. No exposure.
10. Yes, distribution of dataset was considered. P- value was computed numerous times with simulated datasets.

**Gow et al, 2009**

1. Yes, ME/CFS patients and HC were closely matched for age, sex, and ethnicity (all participants were male).
2. Yes, ME/CFS patients and HC were closely matched in place of residence (geographical location).
3. Yes, ME/CFS patients were diagnosed according to the Fukuda criteria and had a history of infectious onset. HC were seven asymptomatic and physically active males with no recent history of infection.
4. N/A. No exposure. This study investigates post-infectious gene signatures in ME/CFS patients compared to HC.
5. N/A. No exposure.
6. Yes, confounding factors included reproductive cycles and contraceptives.
7. Yes, confounding factors were mitigated via exclusion (all participants were male)
8. Yes, human genome-wide Affymetrix GeneChip arrays were conducted.
9. N/A. No exposure.
10. No, Fisher’s exact test was used. No adjustments for multiple comparisons.

**Gravelsina et al, 2021**

1. Unclear if age or sex matched.
2. No participant demographics were provided.
3. No, ME/CFS patients met the Fukuda criteria. No criteria was provided for healthy controls.
4. N/A, no exposure, this study investigated the role of activin B in the pathomechanism of ME/CFS.
5. N/A. No exposure.
6. No, No confounding variables were identified.
7. No, As above.
8. Yes, a validated human activin B ELISA assay was conducted.
9. N/A, no exposure.
10. Yes. This study utilised non-parametric mann-whitney U test or Kruskal- Wallis test or Kruskal-Wallis test and applied post-hoc protocols including Benjamini, Krieger and Yekutieli.

**Groven et al, 2020**

1. Unclear, It was unclear whether patients were age- or sex- matched
2. No, participant demographics were not provided.
3. No, all patients met Fukuda criteria. Although healthy controls health were assessed using a structural medical history reporting, the selection criteria was not provided.
4. N/A. No exposure. Study is investigating immune markers in ME/CFS patients compared to healthy controls using multianalyte profiling Milliplex MAP assay.
5. N/A. No exposure.
6. Yes, history regarding infections, immune disorders, illness in general (somatic as well as psychiatric), medication, menstrual cycle, use of contraceptives, status of menopause, duration of illness (if applicable), and level of physical activity were all recorded as potential confounding variables.
7. Yes, confounding variables were defined as variables with significant associations.
8. Yes, multianalyte profiling Milliplex MAP assay was used.
9. N.A, no exposure.
10. Yes, normality was assessed and appropriate statistical tests were selected. Adjustments for multiple comparisons were also made.

**Günther et al, 2019**

1. Yes, Age and sex- matched
2. No, participants were not stratified by geographical location – participants were located in Barcelona, Madrid and San Sebastian.
3. No, All patients met Canadian Consensus Criteria (CCC). HC criteria was not provided.
4. N/A, immunosignature analysis was conducted in ME/CFS patients compared to HC.
5. N/A. No exposure.
6. No, Confounding factors were not identified.
7. No, As above.
8. Yes, validated immunoassay techniques was conducted.
9. N/A. No exposure.
10. No, the justification of statistical tests was not provided.

**Haffke et al, 2022**

1. Unclear, It is unclear if participants were age or sex- matched because analysis were conducted together with post- COVID-19 condition. ME/CFS patients vs. HC were not statistically investigated.
2. No, no demographic information were supplied.
3. Yes, ME/CFS patients were diagnosed according to the CCC criteria. HC were without a known history of COVID-19.
4. N/A. No exposure. This study investigates endothelial dysfunction and altered endothelial biomarkers in ME/CFS patients compared to HC.
5. N/A. No exposure.
6. Yes, Confounding factors included: diagnosis with COVID-19
7. Yes, this confounding factor was mitigated through inclusion of HC who were diagnosed with COVID-19 to ensure that the significant results were not indicative of post covid rather than ME/CFS.
8. Yes, peripheral endothelial function is assessed by reactive hyperaemia index (RHI) using peripheral arterial tonometry (PAT).
9. N/A. No exposure.
10. No, Kruskal-Wallis test with Dunn’s post-hoc multiple comparisons it was unclear whether they chose on the basis of data distribution as a normality test was not described.

**Halpin et al, 2017**

1. Yes, ME/CFS was age, gender and race and ethnicity matched with healthy controls.
2. No, no participant demographic information was provided.
3. Yes, ME/CFS patients met the Fukuda and CCC criteria. The only criteria that was provided for healthy controls is that they are sedentary no other definitions of healthy controls were provided.
4. N/A. This study investigates the presence of antibodies against herpesviruses in ME/CFS patients.
5. N/A. As above.
6. Yes, Controls that participate in higher levels of exercise. Participants that take immunomodulating medication.
7. Yes, Recruitment of sedentary controls. Exclusion of participants that take immunomodulating medication.
8. Yes, serological assays were conducted.
9. N.A. No exposure.
10. No, Used ANOVA however did not correct for multiple comparisons.

**Hanevik et al, 2012**

1. No, Confounding factors such as age- and sex- matching was adjusted using statistical measures.
2. No, no participant demographic information was provided
3. Yes, ME/CFS patients were defined according to the Fukuda criteria. Healthy controls did not have ME/CFS.
4. N/A. No exposure. This study conducted immunophenotyping in ME/CFS patients.
5. N/A. Exposure.
6. Yes, HIV positive participants, those taking immunomodulating medications or antibiotics, exclusion of other diseases.
7. Yes, All these confounding variables were controlled via exclusion.
8. Yes, flow cytometry was used.
9. N/A. No exposure.
10. Yes, Kruskal-Wallis test was used. A more restrictive significance level was chosen to adjust for multiple comparisons.

**Hardcastle et al 2015A**

1. Yes, ME/CFS patients and HC were age- and sex- matched
2. Yes, Participants were recruited from Queensland and NSW.
3. Yes, ME/CFS patients met the Fukuda criteria. ME/CFS were recognised as having either mild or severe ME/CFS. HC were described as non-fatigued.
4. N/A. No exposure. This study assessed cell functions and receptors in ME/CFS compared to HC.
5. N/A. No exposure.
6. Yes, confounding factors include: severity as well as history of autoimmune disorders, psychosis or other mental health disorder or other physical condition.
7. Yes, confounding factors were mitigated either by grouping based- on illness severity and exclusion of confounding conditions.
8. Yes, flow cytometry was used.
9. N/A. No exposure.
10. Yes, ANOVA or Kruskal Wallis test was selected based on normality. Bonferroni or post-hoc tests were conducted to correct for multiple comparisons. Spearman’s non-parametric correlation was used.

**Hardcastle et al 2015B**

1. Yes, participants were age- and sex- matched.
2. Yes, Participants were from Queensland/ New South Wales.
3. Yes, ME/CFS patients met the Fukuda criteria. Patients were further stratified according to severity. All participants were included if they were previously diagnosed with an autoimmune disorder, psychosis, heart disease or thyroid- related disorders or if they were pregnant, breast feeding or smoking.
4. N/A, there was no exposure. This study longitudinally investigates immune cell abnormalities in ME/CFS patients with different severities of illness.
5. N/A. No exposure.
6. Yes, participants with confounding illnesses such as autoimmune disorder, psychosis or thyroid- related disorders or activities such as smoking were identified as confounding factors.
7. Yes, confounding factors were mitigated via exclusion.
8. Yes, validated flow cytometry techniques were used.
9. N/A. No exposure.
10. Yes, paired T-tests were used or one-way repeated measures ANOVA was conducted. Consideration for multiple comparisons were made by introducing appropriate post-hoc tests.

**Hornig et al, 2015**

1. Yes, Participants were age, sex- and ethnicity- matched
2. Yes, majority of participants were white in race and matched in geographical region of residence.
3. No, ME/CFS patients met the Fukuda or Canadian Consensus Criteria (CCC). A diagnosis of ME/CFS requires duration of illness for more than 6 months. Healthy controls were not defined.
4. N/A. No exposure. This study investigates immune signatures in ME/CFS patients.
5. N/A. No exposure.
6. Yes, participant characteristics such as age may be confounding factors.
7. Yes, confounding factors were mitigated via conduction of statistical analysis controlling for age.
8. Yes, standardised protocols and assays were used to assess cytokine and other biological parameters.
9. N.A. No exposure.
10. 10. Yes, logistic regression modelling was conducted with adjustments for multiple comparisons made.

**Iacob et al, 2016**

1. No, participants were not age- or sex- matched.
2. No, source population information was not provided.
3. Yes, ME/CFS patients were diagnosed according to the Fukuda criteria. Healthy controls had no prior diagnosis of pain, fatigue, or depression.
4. N/A. No exposure. This study investigated gene differences in ME/CFS patients compared to HC using gene expression factor analysis.
5. N/A. No exposure.
6. Yes, Potential confounding factors that were identified included: active viral or upper respiratory infections, chronic cardiovascular or pulmonary disorders, or other chronic conditions, such as anemia or cancer.
7. Yes, confounding factors were mitigated via exclusion.
8. Yes, expression analysis was conducted with validated kits and protocols.
9. N/A. No exposure.
10. No, the study did not adjust for multiple comparisons and had a low P-value. They said further confirmation studies should have more stringent criteria, however, this was not applied to the current study.

**Jawad Kadhum et al**

1. Yes, Participants were age, sex and BMI matched.
2. No, no participant demographic information was provided.
3. No, ME/CFS patients were diagnosed according to the Fukuda criteria. There was no description provided on criteria used to select healthy controls.
4. N/A. No exposure. This study investigates the role of Interleukin-17A Elevation in the pathomechanism of ME/CFS.
5. N/A. No exposure.
6. No, participants completed a variety of blood tests liver and thyroid function, however, it is unclear whether they identified these results as potential confounding variables.
7. No, as above.
8. Yes, this study used clinically validated blood tests.
9. No, No exposure.
10. No, Chi square was used but there was no mention of adjustments made for multiple comparisons.

**Khaiboullina et al, 2015**

1. Yes, It was unclear whether participants were aged- and sex matched.
2. No, Participants were recruited from Belgium and across the united states – no clear indicator of whether participants were matched based on demographic information.
3. No, Patients were diagnosed according to the Fukuda or CCC criteria. HC criteria was not provided.
4. N/A. No exposure. This study investigates cytokine expression in ME/CFS patients compared to HC.
5. N/A as above.
6. No, confounding factors were not identified.
7. No, as above.
8. Yes. This study assesses cytokine expression using a validated protocol and tools clinically validated by the manufacturer.
9. N/A, No exposure.
10. No, a mann-whitney U analysis was selected on the basis of normality. The Kruskal- Wallis test was used to assess whether ME/CFS patients and HC came from the same distribution. No adjustments for multiple comparisons were made.

**Kitami et al, 2020**

1. Yes, Participants were age, sex and BMI matched.
2. No, no patient demographic information was provided.
3. No, ME/CFS patients were diagnosed according to the Fukuda and International Consensus Criteria. Criteria was not provided for healthy controls.
4. N/A. There is no exposure. This study uses deep phenotyping to characterise ME/CFS patients compared to HC.
5. N/A. No exposure.
6. Yes, Possible confounding factors included medications and duration of illness.
7. Yes, Confounding factors such as medications were added as subgroups for example ME/CFS patients that take antidepressants – this was to see the effect these factors have on results observed.
8. Yes, validated phenotyping and transcriptomic protocols were used.
9. N/A. No exposure.
10. Yes, mann-whitney U test was used to determine significant differences between ME/CFS patients and multiple comparisons were corrected for using Benjamini–Hochberg correction method.

**Landi et al, 2016**

1. Yes, Participants were matched for age, sex, BMI and race.
2. Yes, Participants were geographically co-localised.
3. Yes, ME/CFS patients met Fukuda or CCC criteria. HC matched ME/CFS patients in age, sex, BMI and race but were not a household contact.
4. N/A. No exposure. This study investigated levels of IL-16, IL-7 and VEGF-A in ME/CFS patients compared to HC.
5. N/A. No exposure.
6. Yes, age, sex, BMI and race were identified as potential confounding variables.
7. Yes, these confounding variables were mitigated via exclusion.
8. Yes, validated methodologies were used including ELISA to analyse cytokine, chemokine, and growth factors.
9. N/A. No exposure.
10. Yes, appropriate test was selected based on distribution of data. Corrected p-value in consideration for multiple comparisons were made.

**Lindbury et al, 2017**

1. Unclear, Participants age were not significantly different, however, it was unclear whether sex matched.
2. Yes, The study exclusively recruited participants from Donvale (Victoria, Australia)
3. Yes, ME/CFS patients met the CCC criteria. Healthy controls were not related or lived at the same address.
4. Yes there was an exposure. Activin and follistatin were measured following the 20- minute stand test. The 20- minute stand test is a validated protocol.
5. Yes, Activin and Follistatin were measured using clinically validated tools across both ME/CFS patients and HC.
6. Yes, hypothyroidism, Lupus, Fibromyalgia (FM) and Multiple Chemical Sensitivity via the physical and mental status examinations were identified as potential confounding factors.
7. Yes, these confounding factors were mitigated via exclusion.
8. Yes, clinically validated pathology and cytokine analyses were conducted.
9. Yes, the provided time has been validated and shows an effect.
10. No, normality was assessed and an appropriate test was chosen accordingly but adjustments for multiple comparisons were not made.

**Lindbury et al, 2019**

1. Unclear, Participants age were not significantly different, however, it was unclear whether patients were gender matched.
2. Yes, The study exclusively recruited participants from Donvale (Victoria, Australia)
3. Yes, ME/CFS patients met the CCC criteria. Healthy controls were not related or lived at the same address.
4. Yes there was an exposure exposure. Activin and follistatin were measured following the 20- minute stand test. The 20- minute stand test is a validated protocol.
5. Activin and Follistatin were measured using clinically validated measures.
6. Yes, hypothyroidism, Lupus, Fibromyalgia (FM) and Multiple Chemical Sensitivity via the physical and mental status examinations and comorbidities were identified as potential confounding factors.
7. Yes, these confounding factors were mitigated via exclusion.
8. Yes, clinically validated pathology and cytokine analyses were conducted.
9. Yes, the provided time has been validated and shows an effect.
10. No, normality was assessed and an appropriate test was chosen accordingly but adjustments for multiple comparisons were not made.

**Light et al, 2009**

1. Yes, Participants were age and gender- matched with healthy controls.
2. No, No demographic information was provided.
3. No, ME/CFS patients met the Fukuda criteria. Exclusion criteria also include: active upper respiratory infections, use of corticosteroids, SNS agonists, or prescription analgesics known to affect SNS agonists, or prescription analgesics known to affect SNS, HPA, or cytokine activity, and/ or uncontrolled cardiovascular or pulmonary disease. Healthy control criteria was not supplied.
4. Yes, 70% of age-predicted maximal heart rate was calculated. Target heartrate was measured at the 5 minute mark. Participants refrained from exercise for four days.
5. Yes, blood samples were taken at the same time point and so was heart rate and blood pressure.
6. Yes, exercise prior to task was considered a confounding factor. BMI was also considered a confounding variable.
7. Yes, participants were asked to refrain from exercise four days prior to task. BMI was added as a covariate.
8. Yes, PCR was used as well as clinically validated tools to measure heart rate/ blood pressure.
9. Yes, exposure time was sufficient to show a result.
10. No, distribution was skewed due to ddCT method used for mRNA analysis so expression data was log-transformed. Log-transformation is not sufficient to adjust for skewness of data. Post hoc analyses were conducted for ANOVAS.

**Light et al, 2012**

1. No, ME/CFS patients were well matched with HC but ME/CFS patients + Fibromyalgia were not well matched.
2. No, clear demographic information was not provided.
3. Yes, ME/CFS patients met Fukuda and 96% of patients met CCC criteria. Exclusion criteria for all participants include: active viral or upper respiratory infections, chronic cardiovascular or pulmonary disorders, or other chronic conditions such as anaemia, cancer or multiple sclerosis.
4. Yes, this study investigates gene expression alterations at baseline and following moderate exercise. Exercise activity was conducted at the same time each day.
5. Yes, the following outputs being measured include: mRNA via real time, quantitative PCR – the outputs were consistent across ME/CFS patients and HC.
6. Yes, active viral or upper respiratory infections, chronic cardiovascular or pulmonary disorders, or other chronic conditions such as anaemia, cancer or multiple sclerosis were considered confounding variables. Continuous exercise >5 min of walking, for 2 days before, and 2 days after the scheduled exercise task was also identified as a potential confounding variable.
7. Yes, these confounding variables were mitigated via exclusion and requests to refrain from exercise during these time points.
8. Yes, validated methodologies of quantitative PCR was used.
9. Yes, time was sufficient to provide a response.
10. No, one-way ANOVAS between group comparisons were conducted. There was no indication that the significance was adjusted for multiple comparisons.

**Maes et al, 2005**

1. Unclear, unclear if age- or sex- matched.
2. No, no demographic information was provided.
3. No, ME/CFS patients were diagnosed according to the Fukuda criteria. No other criteria was provided apart from that controls were unrelated to ME/CFS patients.
4. N/A. No exposure. This study investigated hormone changes in ME/CFS patients compared to HC.
5. N/A. No exposure.
6. Yes, Confounding factors include: participants who have been treated with neuroleptics, anticonvulsants or mood stabilizers in the past. Those who have taken antidepressants or benzodiazepines for the past 12 months. Those who ever had taken DHEA, growth hormone or acclydine.
7. Yes, confounding factors were mitigated via exclusion.
8. Yes, clinical standard assays were performed including IRMA method, RIA method and electrophoresis.
9. N/A. No exposure.
10. No, Automatic multiple regression was performed. There was no mention of adjustments for multiple comparisons

**Maes et al, 2011**

1. Yes, there was no significant difference in age- and sex- variables between ME/CFS patients and HC.
2. No, ME/CFS patients were outpatients of the Maes clinic in Belgium but no further demographic information was provided regarding HC.
3. No, ME/CFS patients was diagnosed using the Fukuda criteria. HC were related to either the laboratory staff or the patients. Recruitment of relatives of patients may introduce confounding variables as there is a genetic contribution to ME/CFS.
4. N/A. This study investigated the plasma peroxide levels in ME/CFS patients compared to HC.
5. N/A. No exposure.
6. Yes. Potential confounding variables included age, sex, psychiatric disorders, medical illness, pharmacological agents or infection.
7. Yes. Confounding variables were mitigated via exclusion or being added as a cofactor during analysis
8. Yes, a validated commercial method was used: colorimetric assay Oxysta.
9. N/A. No exposure.
10. No, data was adjusted to be normal where appropriate using box-cox transformation, adjustments for multiple comparisons were not made.

**Maes et al, 2012**

1. Yes, there were no significant difference in age and gender- in ME/CFS patients compared to HC.
2. Yes, all participants were associated with attending or working at the Maes clinic in Antwerp
3. Yes, ME/CFS patients were diagnosed according to the Fukuda criteria. Criteria for HC include participants who did not suffer from ME/CFS CF, irritable bowel syndrome, medical and psychiatric illnesses described above and infectious or allergic reactions in the last 2 months prior to blood sampling.
4. N/A. No exposure. This study investigates immune parameters in ME/CFS patients compared to HC.
5. N/A. No exposure.
6. Yes, confounding variables include: life-time diagnosis of psychiatric disorders, those who were treated with immunoregulatory drugs, those who were previously treated with anti-psychotic drugs, those with alcohol abuse or smoking.
7. Yes, confounding variables were mitigated via exclusion.
8. Yes, ELISA and radioimmunoassay was conducted.
9. N/A. No exposure.
10. No, multiple group mean differences were assessed using the Dunn-Scheffe test. Associations were measured using Chi square test. Consistent decisions on whether a parametric or non-parametric test was used for correlation analysis was not made.

**Maes et al, 2015**

1. Yes, there were no significant differences between age and sex.
2. No, clear demographic information was not provided
3. Yes, ME/CFS patients were diagnosed according to the Fukuda criteria. Both ME/CFS patients and HC were excluded if they had a life-time diagnosis of psychiatric axis-1 disorders. Had a medical illness including diabetes type 1. Those who used antipsychotic or antibiotics or immunosuppressant drugs were also excluded as were those who had an infection or allergic reaction in the last two months prior to experiment start date.
4. N/A. There was no exposure. This study investigates activation antigens on CD8+ lymphocytes.
5. N/A. As above.
6. Yes. Potential confounding variables included psychiatric disorders, medical illness, pharmacological agents or infection.
7. Yes. Confounding variables were mitigated via exclusion.
8. Yes. Validated methodologies were utilised including flow cytometry using a FACScalibur.
9. N/A. No exposure.
10. No, Multivariate general linear model (GLM) analyses were used to assess significant differences between variables. Post- hoc test was conducted using Bonferroni’s adjusted comparisons, however, there is no indication of normality test used.

**Maher et al, 2005**

1. Yes, ME/CFS patients were aged and sex- matched
2. No, No demographic information was provided.
3. Yes, ME/CFS patients met the Fukuda definition. Healthy controls were apparently healthy and sedentary.
4. N/A. No exposure. This study investigated perforin levels in ME/CFS patients compared to healthy controls.
5. N/A. No exposure.
6. No, no confounding factors were identified in the study.
7. No, as above.
8. Yes, the study used verified flow cytometry techniques.
9. N/A. No exposure.
10. No, statistics were conducted using students T test- mention of normality tests or rationale for statistical decisions were not provided.

**Mandarano et al, 2018**

1. Yes, ME/CFS patients and HC were aged and BMI matched.
2. No, No demographic information was provided.
3. Yes, ME/CFS patients were diagnosed according to the Fukuda criteria. Participants with acute illness or chronic infectious disease were excluded.
4. No exposure. This study investigates gut microbiota in ME/CFS patients compared to healthy controls.
5. N/A. No exposure.
6. Yes, acute illness or known chronic infectious disease were considered confounding variables.
7. Yes, confounding variables were mitigated through exclusion.
8. Yes, this study uses 18S rRNA sequencing.
9. N/A. No exposure.
10. Yes, normality was checked using Kolmogorov Smirnov test. Wilcoxon Rank Sum test was used.

**Marshall-Gradisnik et al 2016A**

1. Yes, ME/CFS patients were age- and sex- matched.
2. No, No demographic information was provided.
3. Yes, ME/CFS patients were diagnosed according to the Fukuda criteria. Participants reported no medical history, symptoms of prolonged fatigue, or illness of any kind, and were screened for major disease according to routine pathology tests.
4. N/A. This study investigated ion channel specific Single Nucleotide Polymorphisms in NK cells.
5. N/A. No exposure.
6. Yes, confounding factors include: major disease or illness of any kind
7. Yes, confounding factors were mitigated via exclusion – this was distinguished through routine pathology tests.
8. Yes, SNP analysis was conducted via mass array and PCR.
9. N/A. No exposure.
10. No, A two-column chi square analysis was used. No adjustments for multiple comparisons were made.

**Marshall-Gradisnik et al 2016B**

1. Yes, ME/CFS patients were age- and sex- matched.
2. No, No demographic information was provided. Only that participants attended NCNED to provide sample of blood.
3. Yes, ME/CFS patients were diagnosed according to the Fukuda criteria. HC were non-fatigued with no medical history or symptoms of prolonged fatigue or illness of any kind
4. N/A. This study investigated ion channel specific Single Nucleotide Polymorphisms in NK cells.
5. N/A. No exposure.
6. Yes, confounding factors include: pregnant or breastfeeding participants, or those who had a history of smoking or substance use.
7. Yes, confounding factors were mitigated via exclusion – this was distinguished through routine pathology tests.
8. Yes, SNP analysis was conducted via mass array and PCR.
9. N/A. No exposure.
10. Yes, A two-column chi square analysis was used. Yates correction factor was applied.

**Mathew et al, 2009**

1. Yes, Participants were matched for age, sex, body mass index, handedness and IQ.
2. No, ME/CFS patients attended a Private Practice in New York City but no other demographic information was provided.
3. Yes, ME/CFS patients were diagnosed according to the Fukuda criteria. Healthy controls did not have any medical conditions or axis 1 psychiatric disorders.
4. N/A. No exposure. This study investigates lateral ventricular cerebrospinal fluid lactate in ME/CFS patients compared to Healthy controls.
5. N/A. No exposure.
6. Yes, history of psychotic disorder, neurological illness, substance abuse or a persistent medical condition as well as ventricular volume were considered potential confounding variables.
7. Yes, confounding variables were mitigated either by exclusion or making statistical adjustments e.g. ventricular volume (ventricular volume was not significantly different between ME/CFS patients and HC).
8. Yes, MRSI was used.
9. N/A. No exposure.
10. No, tukey HSD and non- parametric mann-whitney tests were used where appropriate. Associations between MRSI lactate and clinical characteristics were conducted using spearman correlation coefficients. There was no mention of adjustments for multiple comparisons.

**Melvin et al, 2019**

1. Yes, Participants were BMI and sex matched.
2. No, Patients were from a UK ME/CFS biobank but no demographic information on HC.
3. Yes, ME/CFS patients were diagnosed according to the Fukuda and CCC criteria. Participants were excluded if they used medications that alter immune function or took any antivirals in the last three months. Had a history of acute or chronic infectious diseases such as hepatitis B and C, tuberculosis, HIV and those with any fatigue illnesses. Healthy controls with a history of fatiguing illness were excluded.
4. No, blood pressure and handgrip strength were reported – parameters were measured using a validated protocol, however, the time points that the parameters were measures were made in 6-12 months across patients – the parameters were not measured in standardised timepoints.
5. Yes, exposure was measured the same between patients and controls.
6. Yes, the following confounding variables include pharmaceutical treatments, infection, other severe physical and psychological illness.
7. Yes, the confounding variables were mitigated through exclusion. Potential confounding variables were also mitigated at the statistics level when conducting a multiple linear regression analysis.
8. Yes, GDF15 was measured in serum using microtitre plate-based two-site electrochemiluminescence immunoassay and the MesoScale Discovery assay platform.
9. Yes, the time was sufficient to provide a response.
10. No, Multiple linear regression analysis was conducted for intergroup comparisons of GDF15 and indicators of severity. Adjustments for multiple comparisons were not made. No mention of assessment of normality was made.

**Metselaar et al, 2021**

1. Unclear, it was unclear whether datasets were age or sex- matched.
2. No, no demographic information was provided.
3. No, ME/CFS patients were defined according to the Fukuda or Canadian Consensus Criteria criteria. Healthy control criteria was not provided.
4. N/A. No exposure. This study investigates robust mRNA expression signatures using recursive ensemble feature collection.
5. N/A. No exposure.
6. No, No confounding factors were identified.
7. No, As above.
8. Yes, the data was taken from a variety of other datasets that use standardised, valid measures for collecting data.
9. N/A. No exposure.
10. No, no statistical methodology was provided.

**Milivojevic, 2020**

1. Yes, participants were matched sex, race/ ethnicity, geographic/ clinical site, and season of sampling.
2. Yes, Participants were matched both in ethnicity and geographically.
3. Yes, ME/CFS patients met either the Fukuda or Canadian Consensus Criteria. HC were assessed by a physician to confirm healthy status. HC were also excluded if they had a history of substance abuse, psychiatric illness, antibiotics (in the past 3 months), immunomodulatory medications in the past year and clinically significant findings on physical exam or screening laboratory tests.
4. N/A. No exposure this study investigates differences in the plasma proteome profile in ME/CFS patients compared to HC.
5. N/A. No exposure.
6. Yes, One of the confounding factors identified was Irritable bowel syndrome.
7. Yes, Effects of IBS was noted through stratification
8. Yes, SpectronautPulsarX was used against human plasma spectra library.
9. N/A. No exposure.
10. Yes. Logistic regression was used. Corrections were made for multiple comparisons.

**Missailidis et al, 2020**

1. Yes, Age and sex- matched
2. No, No source population information provided.
3. Yes, ME/CFS patients met the Canadian Consensus Criteria. HCs had no family history of ME/CFS or fatiguing illness.
4. N/A. No exposure. This study identifies three different potential biomarkers: lymphocyte death rate, mitochondrial respiratory function and TORC1 activity
5. N/A. No exposure.
6. Yes, Freezing ME/CFS patients was identified as a potential confounding variable resulting in increased cell death in ME/CFS cell cultures.
7. Yes, Confounding factor identified but this was not controlled for.
8. Yes, commercial mitochondrial stress test kits and extracellular flux analyser, Seahorse XFp was used.
9. N/A. No exposure.
10. Yes, Mann-Whitney U test was used. Usefulness of biomarker was statistically assessed using AUC with 95% confidence limits.

**Nacul et al, 2018**

1. No, there were significant differences in age and sex between ME/CFS patients and healthy controls. These were adjusted at the statistics level.
2. No, No ME/CFS patients were recruited from the UK ME/CFS biobank, however, no demographic information was supplied for
3. ME/CFS patients were diagnosed according to the Fukuda or CCC criteria. HC were defined as those who did not have any symptoms of severe fatigue or disease.
4. Yes, this study investigates handgrip strength differences between ME/CFS patients and healthy controls.
5. Yes, exposure was measured in the same way for ME/CFS patients and HC.
6. Yes, confounding factors identified include: recent use of medications that alter immune function, anti-viral medications, and vaccinations. History of acute infections. Those who are pregnant or within 12 months post-partum or currently lactating. Age, gender and BMI were also identified as potential confounding variables.
7. Yes, these confounding factors were mitigated via exclusion. Age, gender and BMI were added as cofactors.
8. Yes, a handheld dynamometer was used as well as clinical pathology results.
9. Yes, the time was sufficient to get a response. With added time the response was more significant.
10. No, no assessment of normality was conducted nor was there an adjustment made for multiple comparisons.

**Nacul et al, 2019**

1. Unclear, it was unclear if age and sex- matched.
2. No, no demographic information was provided.
3. No, ME/CFS patients were diagnosed according to the Fukuda and CCC criteria. There was no criteria provided for HC.
4. N/A, No exposure, this study has investigated clinical pathology abnormalities in ME/CFS patients compared to HC.
5. N/A. No exposure.
6. Yes, Blood tests were conducted to assess for any potential confounding factors in ME/CFS patients that may justify a different diagnosis.
7. Yes, confounding factors were mitigated via exclusion
8. Yes, this study investigates clinical laboratory results.
9. N/A. No exposure.
10. Yes, adequate sample size was selected. Normality was assessed and appropriate tests were selected. Sample size was selected so that only 1% of the outcomes were Type 1 errors. Bonferroni adjustments for multiple comparisons were made.

**Nagy- Szakal et al, 2017**

1. Yes, ME/CFS patients and HC were matched in age, sex, race/ethnicity, geographic/clinical site and season of sampling.
2. Yes. ME/CFS patients were matched in race/ ethnicity and geographic location.
3. Yes, ME/CFS patients were diagnosed according to Fukuda or CCC criteria. HC were free from any conditions similar to ME/CFS or any clinically significant illness as assessed by physician.
4. N/A. This study investigates metabolomic differences in ME/CFS patients compared to HC.
5. N/A. No exposure.
6. Yes, Confounding factors include IBS, substance abuse, antibiotics in prior 3 weeks, immunomodulatory medications, and any significant findings on physical or laboratory- based tests.
7. Yes, Most confounding factors were mitigated by exclusion. Patients with IBS were segregated from those without IBS.
8. Yes, Nuclear magnetic resonance (NMR) spectroscopy was used.
9. N/A. No exposure.
10. Yes, Normalisation was performed. Non-parametric mann- whitney U test was used and adjusted univariate logistic regression.

**Nagy- Szakal et al, 2018**

1. Yes, ME/CFS patients and HC were matched in age, sex, race/ethnicity, geographic/clinical site and season of sampling.
2. Yes. ME/CFS patients were matched in race/ ethnicity and geographic location.
3. ME/CFS patients were diagnosed according to Fukuda or CCC criteria. HC were free from any conditions similar to ME/CFS or any clinically significant illness as assessed by physician.
4. N/A. This study investigates metabolomic differences in ME/CFS patients compared to HC.
5. N/A. No exposure.
6. Yes, Confounding factors include IBS, substance abuse, antibiotics in prior 3 weeks, immunomodulatory medications, and any significant findings on physical or laboratory- based tests.
7. Yes, Most confounding factors were mitigated by exclusion. Patients with IBS were segregated from those without IBS.
8. Yes, Nuclear magnetic resonance (NMR) spectroscopy was used.
9. N/A. No exposure.
10. Normalisation was performed. Non-parametric mann- whitney U test was used and adjusted univariate logistic regression.

**Natelson et al, 2007**

1. Yes, patients were age- and sex- matched.
2. No, no demographic information was provided.
3. Yes, ME/CFS patients were diagnosed according to the Fukuda criteria. Healthy control criteria were that they were sedentary.
4. N/A. No exposure. This study investigates levels of ventricular lactate in ME/CFS patients compared to HC.
5. N/A. No exposure.
6. Yes, brain active medications and BMI were considered potential confounding variables.
7. Yes, patients that took these medications were excluded. BMI was statistically addressed as a co-variate.
8. Yes, proton magnetic resonance spectroscopic imaging (1H MRSI) was used.
9. N/A. No exposure.
10. Yes, Normality was assessed. Regression analysis was conducted andcorrected using Bonferroni adjustment.

**Natelson et al, 2017**

1. Yes, patients were age- and sex- matched.
2. No, no demographic information was provided.
3. Yes, ME/CFS patients were diagnosed according to the Fukuda criteria. Healthy control were defined as sedentary.
4. N/A. No exposure. This study investigates levels of ventricular lactate in ME/CFS patients compared to HC.
5. N/A. No exposure.
6. Yes, brain active medications and BMI were considered potential confounding variables. Sex was also a potential confounding variable.
7. Yes, patients that took these medications were excluded. BMI was statistically addressed as a co-variate. As there were no male controls, male ME/CFS patients were excluded from analysis.
8. Yes, proton magnetic resonance spectroscopic imaging (1H MRSI) was used.
9. N/A. No exposure.
10. Yes, Normality was assessed. Post-hoc comparison was conducted.

**Nelson et al, 2019**

1. Unclear, this sample was taken from a larger sample that was age, sex and BMI matched but the author details that the matching was selected on the basis of convenience. No statistical analysis has been conducted.
2. Unclear, ME/CFS patients were recruited from Adelaide, South Australia Greater Metropolitan area – ,HC were recruited from patient and research centre networks however it isn’t clear what location they are recruited from.
3. Yes, ME/CFS met either Fukuda, CCC or ICC criteria. All participants were sedentary, were not taking any medications, or had any medical conditions that could alter HR (HC).
4. Yes, exposure was measured with a fitted heart monitor.
5. Yes, the same protocol was used for ME/CFS patients and HC.
6. Yes, confounding factors may arise through to familiarisation (practicing protocol prior to assessment) due to symptom exacerbation following exercise.
7. Yes, confounding factors due to familiarisation was mitigated through familiarising with protocol through reading for both groups – no practice of the task was conducted by either of the groups.
8. Yes, a fitted heartrate monitor was used and data was extracted through polar protrainer software.
9. Yes, the time given was sufficient time to see a response.
10. No, Normality was assessed using Shapiro-Wilk. Two-way repeated measures ANOVA was conducted however, no adjustment for multiple comparisons were made.

**Nelson et al, 2021**

1. Unclear, this sample was taken from a larger sample that was age, sex and BMI matched but the author details that the matching was selected on the basis of convenience. No statistical analysis has been conducted.
2. Unclear, ME/CFS patients were recruited from Adelaide, South Australia Greater Metropolitan area – ,however, it was unclear whether HC were recruited from the same area.
3. Yes, ME/CFS met either Fukuda, CCC or ICC criteria. All participants were sedentary, were not taking any medications, or had any medical conditions that could alter HR (HC).
4. Yes, exposure was measured with a fitted heart monitor.
5. Yes, the same protocol was used for ME/CFS patients and HC.
6. Yes, confounding factors may arise through to familiarisation (practicing protocol prior to assessment) due to symptom exacerbation following exercise.
7. Yes, confounding factors due to familiarisation was mitigated through familiarising with protocol through reading for both groups – no practice of the task was conducted by either of the groups.
8. Yes, a fitted heartrate monitor was used and data was extracted through polar protrainer software.
9. Yes, the time given was sufficient time to see a response.
10. No, Normality was assessed using Shapiro-Wilk. Two-way repeated measures ANOVA was conducted however, no adjustment for multiple comparisons were made.

**Nepotchatykh et al, 2020**

1. Yes, participants were age- and sex- matched.
2. No, No demographic information was provided.
3. Yes, ME/CFS participants were diagnosed according to the CCC criteria. HC had no family history of ME/CFS.
4. Yes, changes via the stress test was measured
5. Yes, post-exertional stress challenge was conducted. This was consistent across ME/CFS patients and HC. All participants were mechanically stimulated for 90 min to induce PEM and evaluate changes in the miRNA expression profile in response to this mechanical stimulation
6. No, confounding variables were not identified.
7. No, As above.
8. Yes, QPCR was used with validated protocols.
9. Yes, the time was sufficient to provide a response.
10. No, this study does not provide rationale or information as to why they chose particular statistical tests. ANOVA and Tukey multiple comparison test was conducted, however, normality was not assessed to assess data distribution.

**Nguyen et al, 2017**

1. No, ME/CFS patients were matched in sex but not in age.
2. Yes, Participants resided in South-East QLD region of Australia
3. Yes, ME/CFS patients met Fukuda criteria. All participants had no previous history of smoking or chronic diseases.
4. N/A. No exposure. This study investigates calcium mobilization in NK cells from ME/CFS patients compared to HC.
5. N/A. No exposure.
6. Yes, confounding factors include pregnant, breast-feeding or taking hormone therapy or pharmaceutical immunoregulatory medications.
7. Yes, pharmaceutical treatments were discontinued two weeks prior to blood collection. Justification for half-life of medications that are being stopped two weeks prior to experiment participation should be provided.
8. Yes, FloJo was used.
9. N/A. No exposure.
10. No, Mann- Whitney U test was selected, however, there was no justification of distribution of data. Gaussian Smoothing method was used to determine area under the curve of Ca2+ influx.

**Nijs et al, 2010**

1. Yes, ME/CFS patients were all female and were aged matched.
2. No, no demographic information was provided.
3. Yes, ME/CFS patients were diagnosed according to the Fukuda criteria. Sedentary HC were described as having a seated profession and performing a maximum of 1 hour of sports per week.
4. Yes, exposure was measured using self-reported measures SF-36 with 97% test- retest reliability and reliability and validity in a wide variety of patient populations. CIS is also well validated. Complement, elastase activity and interleukin 1 beta were also measured using validated methodologies.
5. Yes, exposure was measured the same way across participants.
6. Yes, confounding variables include medication such as analgesics and antidepressants
7. Yes, confounding variables were mitigated by stopping medication a week prior and for the duration of the study.
8. Yes,  *in vitro* sandwich enzyme immunoassay and ELISA was used.
9. Unclear, it is unclear whether 24 hours was sufficient to elicit physiological changes in response due to post-exertional malaise.
10. No, Sample size was determined through a post-hoc analysis of a previous study. Justification of test selection based on distribution was provided. Adjustments for multiple comparisons were not made.

**Nkiliza et al, 2021**

1. Yes, Participants were matched for age, sex, and BMI.
2. No, Participants were matched by ethnicity, however, not by geographical location.
3. Yes, ME/CFS patients were diagnosed according to Fukuda and CCC criteria. HC did not meet any of the case definitions of ME/CFS and were sedentary.
4. N/A. No exposure. This study investigates plasma lipid profiles in ME/CFS patients compared to HC.
5. N/A. No exposure.
6. Yes, confounding factors that were identified includes: those who have been diagnosed with a DSM-IV mental disorder, those with a history of substance abuse, chronic or systemic disorders and those who took concomitant medication.
7. Yes, confounding factors were mitigated via exclusion.
8. Yes, nano-flow liquid chromatography (nLC) and high-performance liquid chromatography (HPLC) systems coupled with a high mass accuracy ORBITRAP mass spectrometer was used.
9. N/A. No exposure.
10. No, Normality was assessed. Non-normally distributed data was log-transformed. There is insufficient explanation for log- transformation and not instead using a non-parametric test for non-normal distributed data.

**Okada et al, 2004**

1. No, participants are aged- matched but not sex-matched.
2. Unclear, participants were recruited from the outpatient fatigue clinic in Osaka University, however, it is unclear whether this includes HC.
3. No, ME/CFS patients met Fukuda case definition. No criteria for HC was provided.
4. N/A. No exposure. This study uses MRI to investigate neurological changes in ME/CFS patients and compare with fatigue ratings.
5. N/A. No exposure.
6. Yes, age, sex and whole segment gray or white matter volume differences were identified as confounding variables
7. Yes, these variables were adjusted using statistical adjustments as a covariable.
8. Yes, Voxel- based morphometric MRI was used
9. N/A. No exposure.
10. Yes, a non-parametric test was used as data was not normal. Study was adjusted for multiple comparisons.

**Petty et al, 2016**

1. Yes, participants were aged- and sex- matched.
2. No, no demographic information was provided.
3. Yes, ME/CFS patients met the Fukuda criteria and the CCC criteria. Both ME/CFS and HC were excluded if they smoked in the previous year, took antibiotics, steroids, antidepressants (at least 3 months prior to the study).
4. N/A, No exposure. This study investigates MicroRNA as a potential diagnostic marker for ME/CFS.
5. N/A. No exposure.
6. Yes, Confounding variables included those who took certain classes of medication and smoking or drinking status
7. Yes, confounding variables were mitigated via exclusion.
8. Yes, QRTPCR was used.
9. N/A. No exposure.
10. No, considerations on data distributions were not made. Pearson correlation and ROC analysis was conducted.

**Powell et al, 2003**

1. Unclear, It is unclear whether ME/CFS patients were age or sex matched with HC.
2. No, Participants were all Caucasian but there was no indication whether participants were also matched geographically.
3. No, ME/CFS patients were diagnosed according to the Fukuda criteria. HC criteria was not provided.
4. N/A. No exposure. This study investigated differentially displayed sequences in the leucocytes of ME/CFS
5. N/A no exposure.
6. Yes, confounding factors include those who are on steroid treatment.
7. Yes, the confounding factor was mitigated via exclusion.
8. Yes, RT PCR was used.
9. N/A no exposure.
10. No, T- test was used, however, no information on data distribution was provided

**Provenzano et al, 2020**

1. Yes, Patients were age and BMI matched but not sex matched. There is no evidence differences in performance in the N- back test in males and females. Age and gender were controlled in the data build.
2. No, no demographic information was provided.
3. No, ME/CFS was diagnosed according to the Fukuda criteria. Criteria for HC was not provided.
4. Yes, a validated N-back test was utilised.
5. Yes, The N- back test was conducted using the same methodology for ME/CFS patients and HC.
6. Yes, Collinearity was mentioned as a potential confounding variable
7. Yes, Collinearity was controlled through use of a recursive feature elimination instead of stepwise logistic regression.
8. Yes, fMRI was used
9. Yes, the time points have been clinically validated previously
10. No, There were no details on whether the distribution of the data influenced the choice of statistical tests.

**Rayhan et al, 2021**

1. Unclear if age or sex matched
2. No, no demographic information was provided.
3. Yes, ME/CFS patients were diagnosed according to Fukuda or CCC criteria. HC were sedentary and did not have any serious medical or psychiatric conditions.
4. Yes, exposures were measured using validated protocols.
5. Yes, exposure was measured in the same way in ME/CFS patients as HC.
6. Yes, confounding factors include: non-sedentary controls or serious medical or psychiatric condition
7. Yes, Confounding factors were mitigated through exclusion.
8. Yes, fMRI was used.
9. Yes, Time was sufficient to see a result.
10. Unclear, It is unclear whether they assessed for normality. ANOVA and Student’s T test was used. Bonferroni correction and Tukey Honest Significant Difference were also used.

**Rivas et al, 2018**

1. Yes, ME/CFS patients were matched for age and gender.
2. No, it is unclear whether participants demographic information was provided a part from site locations where data was collected.
3. Yes, ME/CFS patients fulfilled the CCC criteria. ME/CFS patients were not a first or second degree relative of ME/CFS patients.
4. N/A. No exposure. This study investigated T and NK cell phenotypes.
5. N/A. No exposure.
6. No, confounding variables were not identified.
7. No, No confounding variables were identified.
8. Yes, flow cytometry was used.
9. N.A. No exposure
10. Yes, normality was considered in selection of test and multiple corrections were applied to account for false-discovery rate.

**Saiki et al, 2008**

1. Yes, participants were age- and sex- matched.
2. No, no demographic information was supplied.
3. Unclear, ME/CFS patients were diagnosed according to the Fukuda criteria. HC underwent comprehensive medical examination for past and present health problems, however, it is not clear whether the results influenced selection of participants.
4. N/A. No exposure. This study investigates marker genes for ME/CFS.
5. N/A. No exposure.
6. Yes, medications were identified as a potential confounding variable.
7. Yes, all participants stopped taking medication three months prior to participating in the study.
8. Yes, microarray were used.
9. N/A. No exposure.
10. No, multiple comparisons were addressed through adjustment, however, details of test selection including normalisation of data was not described.

**Shan et al, 2018**

- - - 1. No, participants were not matched – differences in gender and age were statistically adjusted.
      2. No, no demographic information was supplied.
      3. No, ME/CFS patients were diagnosed according to the Fukuda criteria. HC criteria was not described.
      4. Yes, Stroop task was administered. Exposure was administered in a consistent manner.
      5. Yes, exposure was measured in the same way for cases and controls.
      6. Yes, Medications especially those targeting the central nervous system were confounding factors.
      7. Yes, confounding factors were mitigated via exclusion.
      8. Yes, functional MRI was used.
      9. Yes, time was sufficient for a response.
      10. No, Pearson correlation was used, however, it was unclear whether normality of data was considered. Adjustments for multiple comparisons were not made.

**Shishioh-Ikejima et al, 2010**

Yes ME/CFS patients were age- and sex- matched. There was no correlation between BMI and A- MSH

No, no demographic information was provided.

Yes, ME/CFS patients fulfilled the Fukuda criteria and healthy controls had no complaints of fatigue or other diagnosis.

N/A. This study investigates hormone concentrations in ME/CFS patients compared to HC.

N/A. No exposure.

No, the only confounding factor that was identified was duration of symptoms above 10 years, however, it isn’t really clear why this is a confounding factor.

No, As above.

Yes, a commercial radioimmunoassay was used.

N/A, no exposure

Yes, appropriate statistical tests were selected. Normality was considered with selection of non-parametric tests.

**Shukla et al, 2015**

1. Yes, ME/CFS patients and HC were matched for age, gender, BMI and general activity patterns.
2. Yes, Participants were recruited from the Madison and Marshfield, Wisconsin area.
3. Yes, ME/CFS patients met the Fukuda criteria. HC did not have any complaints of persistent fatigue.
4. Yes, exercise tests were administered. Resistance was software controlled.
5. Yes, exposure was measured in the same way for cases and controls.
6. Yes, the confounding factors that were identified included current use of immunomodulatory medications, stool softeners, laxatives, anti-diarrheal agents, antibiotics, or probiotics, use of opioids, history of cardiovascular disease or uncontrolled hypertension or current fatigue sufficient to interfere with exercise testing. Additionally, confounding variables that lead to multicollinearity and artificial inflation of variance
7. Yes, confounding factors were mitigated by exclusion. Specific variables were selected to avoid multicollinearity or inflation of variance.
8. Yes, 16S RNA sequencing was conducted.
9. Yes, sufficient time was provided to see a response.
10. Yes, adjustments for multiple comparisons and considerations for normality were made.

**Simonato et al, 2021**

1. Yes, age and sex- matched.
2. No, no demographic information was provided.
3. Yes, ME/CFS patients were diagnosed according to the Canadian Consensus Criteria (CCC). HC criteria included absence of any current or past psychiatric, neurological, or other known medical conditions.
4. N/A. No exposure. This study investigates tryptophan metabolites and cytokines etc in ME/CFS patients compared to HC.
5. N/A. No exposure.
6. Yes, Confounding factors include: family and medical history, menstrual cycle, bowel habits, smoking, alcohol, consumption, use of contraception.
7. No, Although information was collected it was unclear how the authors used this information in stratifying or selecting participants.
8. Yes, this study used ELISA
9. N/A. No exposure.
10. No, Data was assessed using mann-whitney test and chi-square or Fisher’s exact test. Correlations were assessed using Spearman’s rank-sum. Adjustments for multiple comparisons was not made and there justification was not sufficient (due to it being a exploratory study).

**Singh et al, 2016**

1. Unclear if age and sex matched
2. No, Although USA and European patients were selected and they were grouped accordingly – further stratification is required due to vastness of both continents and no further information has been provided.
3. No, ME/CFS patients met CCC and Fukuda criteria. HC criteria was not described.
4. N/A. No exposure. This study investigates whether humoral immunity profiling is able to effectively differentiate between cases and controls.
5. N/A. No exposure.
6. No, confounding variables were not identified.
7. No, as above.
8. Yes, this study used random peptide microarray.
9. N/A. No exposure.
10. No, this study does not provide any information on what statistical tests were utilised.

**Snell et al, 2013**

1. Yes, ME/CFS patients and HC were age, sex and BMI matched.
2. No, No demographic information was provided.
3. Yes, ME/CFS patients were diagnosed according to the Fukuda criteria. All participants were sedentary, but no other criteria were provided for HC.
4. Yes. Breath by breath gas samples were collected by a commercial mask. Electrocardiography and blood pressure were also collected using clinical validated tools.
5. No, although participants were requested to maintain a certain pedalling cadence of 60 to 80 rpm there was no way to maintain the speed between ME/CFS patients and controls.
6. No, No confounding variables were identified.
7. No. As above.
8. Yes, gas exchange data, electrocardiography and blood pressure were collected using clinically validated tools.
9. Yes, the time provided was sufficient to show significant differences in test 2.
10. No, Box’s M test assessed variance- covariance of data and appropriate statistical test was selected on the basis of this result. Corrections for multiple comparisons were made through post-hoc tests.

**Sorenson et al, 2003**

1. Yes ME/CFS patients and HC were matched for age, sex, BMI and activity level.
2. No, Controls included those of Asian descent while ME/CFS patients only had Caucasians.
3. No, ME/CFS patients were diagnosed according to the Fukuda criteria. No criteria was provided for HC apart from those with or without allergies.
4. Yes, bicycle task was conducted using previously described protocols at 70% of maximal workload.
5. Yes, The same cycling, histamine task and allergen were conducted using the same protocol for ME/CFS patients and HC.
6. Yes, one of the potential confounding factors is those with or without allergies.
7. Participants were stratified based on allergic and non-allergic. Those with asthma were excluded.
8. Yes, ELISA was used.
9. Yes, the exposure time was sufficient to show responses.
10. Yes, non-parametric test was used because ME/CFS patients had a greater variance compared to HC. Multivariate ANOVA was used and post-hoc analyses were conducted.

**Sorland et al, 2021**

1. Unclear, Age-matched but unclear if sex-matched.
2. No, No demographic information was supplied.
3. No, ME/CFS patients were diagnosed according to the CCC criteria. No information was provided on criteria used to select HC.
4. N/A. Although this study did have an intervention – the intervention was not investigated in this systematic review (SR).
5. N/A. Not applicable to this SR.
6. Yes, confounding factors include food, smoking and medications. The setting of the experiment may also introduce confounding factors.
7. Yes, Participants were asked to restrict food, fluids and medications 8 hours prior to assessment. The setting of the room was temperature and light controlled.
8. Yes, a clinical blood pressure cuff was used.
9. N/A. No exposure.
10. No, The study had good justification as to why a non-parametric test was used. Comparisons were made using Mann-Whitney U test or Kruskal- Wallis, or paired rank sum test (depending on which variables were being assessed. Correlations were assessed using Spearman nonparametric correlation. No corrections for multiple comparisons were made.

**Stringer et al, 2013**

1. Yes, ME/CFS patients was age, sex and BMI matched with controls
2. No, no participant demographic information was provided.
3. Yes, ME/CFS patients met Fukuda criteria. All participants were excluded if they had the following: autoimmune disease, Lyme disease, elevated viral load, active infection, pregnancy.
4. N/A. No exposure. Study investigates daily cytokine fluctuations.
5. N/A. No exposure.
6. Yes, confounding factors were identified including: autoimmune disease, Lyme disease, elevated viral load, active infection, pregnancy. Leptin increases with age.
7. Yes, Confounding factors were mitigated via exclusion and age-matching.
8. Yes, a commercial cytokine panel was used.
9. N/A. No exposure.
10. Yes, Multivariate tests were conducted. P-value was adjusted for multiple comparisons. Data was validated as a proof-of-concept.

**Sung et al, 2020**

1. Yes, ME/CFS patients and HC were age, sex and race matched.
2. Yes, Blood was collected from Salt Lake City. Participant family members were selected on geographical location and accessibility of blood collection centre. HC were matched by race with ME/CFS patients.
3. Yes, ME/CFS were defined according to the Fukuda criteria. HC were defined as HIV-negative, no overt infections at time of blood donations, without ME/CFS.
4. N/A. No exposure. Study investigates antibody-dependent cell- mediated cytotoxicity in ME/CFS, related family members and non-related controls.
5. N/A. No exposure.
6. No confounding variables were identified.
7. No. As above.
8. Yes, NK cell counts were determined using Flow Jo and Trucount commercially validated product. ADCC was determined using Cr-release.
9. N/A. No exposure.
10. No, Logistic regression was used for comparisons, however, it was unclear whether adjustments for multiple comparisons were made.

**Sweetman et al, 2020**

1. Yes, ME/CFS patients and HC were age and sex- matched.
2. No, No patient demographics were provided.
3. Yes, ME/CFS patients were diagnosed according to the CCC criteria. HC had no history of significant illness or fatigue related disorders.
4. N/A. No exposure. Proteomes of PBMCs from ME/CFS patients and HC was investigated using SWATH-MS analysis.
5. N/A. No exposure.
6. Yes, a potential confounding factor that was identified was subgrouping. Age, gender and BMI differences were also potential confounding factors.
7. No, PCA cluster differences could not be described by age, gender, and BMI differences. Patients were not sub-grouped.
8. Yes, a SWATH-MS analysis was conducted.
9. N/A. No exposure.
10. No, it was unclear why they selected certain statistical tests (ie. Normality).

**Szlarski et al, 2021**

1. No, Participants were not age- and sex- matched. These parameters were adjusted statistically.
2. No, although participants all attended the same clinic and HC were all workers at the clinic it was unclear whether demographic information was considered – no other information was provided.
3. Yes, ME/CFS patients were diagnosed according to the CCC criteria. HC did not have any affecting diagnoses.
4. N/A. No exposure. This study investigated soluble CD26 and autoantibodies in ME/CFS patients, post- COVID-19 patients and HC.
5. N/A. No exposure.
6. Yes, age- and sex- were identified as potential confounding variables.
7. Yes, they were adjusted statistically.
8. Yes, FloJo was used.
9. N/A. No exposure.
10. Yes, Mann- Whitney- U, and rank-sum tests were used to compare quantitative parameters Kruskal-Wallis with Dunn’s post hoc was used. Chi square test was used for categorical variables and Benjamini-Yekuteieli (BY) correction was applied.

**Thambirajah et al, 2008**

1. Yes, age and sex- matched.
2. No, no patient demographics were provided.
3. Yes, ME/CFS patients were diagnosed according to the Fukuda criteria. HC had no known underlying illness and did not undertake strenuous exercise.
4. Yes, the protocol was amended from a previously validated Bruce protocol.
5. Yes, the time and speed of the treadmill exercise was the same for ME/CFS patients. and HC. Blood was collected at the same time points.
6. Yes, prior illness and activity level were considered confounding variables.
7. Yes, Confounding variables were mitigated through exclusion criteria.
8. Yes, Western blotting Epson Expression 1600 scanner was used.
9. Yes, the timepoints were sufficient to provide a result.
10. No, It is unclear whether data distribution has been assessed for appropriateness of tests selected. Adjustments for multiple comparisons were not made for ANOVA.

**Thapaliya et al, 2021**

1. Yes, ME/CFS patients were age- but not sex- matched with HC.
2. Yes, the scanning took place at Gold Coast hospital and participants travelled within the vicinity.
3. Yes, ME/CFS patients were diagnosed according to either the Fukuda or ICC. HC did not have any exclusionary medical disorders and no abnormal physical function.
4. N/A. There were no exposures. This study investigates neuronal microstructure changes using diffusion Tensor Imaging.
5. N/A. No exposure.
6. Yes, ADHD, autoimmune disease, microvascular disease or high BMI were considered confounding variables.
7. Yes, confounding factors were mitigated via exclusion.
8. Yes, Diffusion Tensor Imaging was conducted.
9. N/A no exposure
10. Yes, a two sample T- test was conducted controlling for age and gender was conducted. Voxel clusters was also conducted with false-discovery rate corrected. Correction for multiple regressions conducted.

**Theorell et al, 2017**

1. Unclear if age or sex- matched.
2. Yes, participants were recruited in Stockholm and Oslo – the data from both regions were interpreted separately.
3. No, ME/CFS patients were diagnosed according to the CCC criteria. All that is known about HC is age and sex- no other additional information was provided.
4. N/A. There were no exposures. This study investigated cytotoxic lymphocyte function in ME/CFS patients compared to HC.
5. N/A. No exposure.
6. No, No confounding factors were identified.
7. No, as above.
8. Yes, flow cytometry was used.
9. N/A. No exposure.
10. No, Correction for multiple comparisons was made, however, details on selection of statistical tests were not provided.

**Tiev et al, 2003**

1. Yes, ME/CFS patients and HC were age and sex- matched.
2. No, there was no demographic information supplied.
3. Yes, ME/CFS patients were diagnosed according to Fukuda criteria. Biological tests were conducted for both groups to ensure exclusion of organic diseases.
4. N/A. No exposure. This study investigates the use of 37-Kilodalton/83-Kilodalton Isoform Ratio test as a diagnostic measure for ME/CFS compared to HC.
5. N/A. No exposure.
6. Yes, infectious history or psychiatric illness were considered confounding factors
7. Yes, there was no evidence of infectious history or psychiatric illness in either groups.
8. Yes, 37-Kilodalton/83-Kilodalton Isoform Ratio was conducted.
9. N/A. No exposure.
10. No, Mann Whitney U test was selected, however, there was no description of a normality test being conducted in order to determine whether this was an appropriate test selected.

**Tokunaga et al, 2020**

1. Yes, participants were age- and sex- matched.
2. Yes, All participants were Caucasian and were from Salt Lake City.
3. No, patients were diagnosed according to the Fukuda criteria. The only criteria that was given for HC was that they were unrelated to ME/CFS patients. Only criteria was provided for non-affected family members.
4. N/A. No exposure. This study investigates CD16A-positive anti-inflammatory monocytes in ME/CFS compared to HC.
5. N/A. No exposure.
6. Yes, time of blood withdrawals was considered a confounding variable.
7. Yes, Almost all blood collections were conducted on the same day.
8. Yes, flow cytometry was used.
9. N/A. No exposure.
10. No, Student T- test was used – variances were assumed. Chi Square with Yates correction and Fisher’s exact tests were applied.

**Vernon et al, 2005**

1. Yes, Participants were age, sex and race matched.
2. Yes, participants were from the general Atlanta population.
3. No, ME/CFS patients were diagnosed according to Fukuda criteria. Criteria was not provided for HC.
4. N/A. No exposure. This study investigates autoantibodies and neuronal cell antigens in ME/CFS compared to HC.
5. N/A. No exposure.
6. Yes, Sex, age, age at ill-ness onset, and duration of illness were considered potential confounding variables.
7. Yes, Confounding variables were mitigated through stratification of different groups.
8. Yes, Commercial assay kits were used for ELISA and immunoblot.
9. N/A no exposure.
10. No, Fisher’s exact probability test was used to assess distribution of autoantibodies. A non-parametric test was selected – distribution of data was not described.

**White et al, 2012**

1. Yes, Participants were age and gender- matched with healthy controls.
2. No, No demographic information was provided.
3. No, ME/CFS patients met the Fukuda criteria. Healthy control criteria was not supplied.
4. Yes, 70% of age-predicted maximal heart rate was calculated. Target heartrate was measured at the 5 minute mark. Participants refrained from exercise for four days.
5. Yes, blood samples were taken at the same time point and so was heart rate and blood pressure.
6. Yes, exercise prior to task was considered a confounding factor.
7. Yes, participants were asked to refrain from exercise four days prior to task.
8. Yes, PCR was used as well as clinically validated tools to measure heart rate/ blood pressure.
9. Yes, exposure time was sufficient to show a result.
10. No, distribution was skewed due to ddCT method used for mRNA analysis so expression data was log-transformed. Log-transformation is not sufficient to adjust for skewness of data. Post hoc analyses were conducted for ANOVAS.

**Zeinah et al, 2015**

1. Yes, Participants were age and sex- matched.
2. No, no demographic information was supplied.
3. Yes, ME/CFS patients met the Fukuda criteria. HC criteria includes no history of major depression, ME/CFS or chronic fatigue or substance abuse.
4. N/A. No exposure. This study investigates MRI changes in ME/CFS patients compared to HC.
5. N/A. No exposure.
6. Yes, age, total intercranial volume, disease and handedness were considered potential confounding variables.
7. Yes, these factors were considered independent variables in the regression.
8. Yes, MRI with diffusion tensor imaging/arterial spin labelling was used.
9. N/A. No exposure.
10. No, regression was corrected for multiple comparisons. Pearson correlation was used however it was unclear whether considerations for data distributions were made.
